# Unraveling the Atomistic Mechanism of Electrostatic Lateral Association of Peptide β‐Sheet Structures and Its Role in Nanofiber Growth and Hydrogelation

**DOI:** 10.1002/smll.202408213

**Published:** 2025-01-09

**Authors:** Mohamed A. N. Soliman, Abdulwahhab Khedr, Tarsem Sahota, Rachel Armitage, Raymond Allan, Katie Laird, Natalie Allcock, Fatmah I. Ghuloum, Mahetab H. Amer, Reem Alazragi, Charlotte J. C. Edwards‐Gayle, Jacek K. Wychowaniec, Attilio V. Vargiu, Mohamed A. Elsawy

**Affiliations:** ^1^ Leicester Institute for Pharmaceutical Innovation Leicester School of Pharmacy De Montfort University The Gateway Leicester LE1 9BH UK; ^2^ Department of Pharmaceutics and Industrial Pharmacy Faculty of Pharmacy Cairo University Cairo 11562 Egypt; ^3^ Department of Pharmaceutics and Industrial Pharmacy Faculty of Pharmacy Zagazig University Zagazig Egypt; ^4^ School of Archaeology and Ancient History University of Leicester Leicester LE1 7RH UK; ^5^ Electron Microscopy Facility Core Biotechnology Services College of Life Sciences University of Leicester Leicester LE1 7RH UK; ^6^ Division of Cell Matrix and Regenerative Medicine School of Biological Sciences University of Manchester Oxford Road Manchester M13 9PL UK; ^7^ Department of Biological Science College of Science University of Jeddah Jeddah 21493 Saudi Arabia; ^8^ Diamond Light Source Harwell Science and Innovation Campus Fermi Avenue Didcot OX110DE UK; ^9^ AO Research Institute Davos Clavadelerstrasse 8 Davos 7270 Switzerland; ^10^ Physics Department University of Cagliari s.p. 8 km. 0.700 Monserrato 09042 Italy; ^11^ Division of Pharmacy and Optometry School of Health Sciences University of Manchester Oxford Road Manchester M13 9PL UK

**Keywords:** charge‐zipper, hydrogels, nanofibers, peptides, self‐assembly

## Abstract

Guiding molecular assembly of peptides into rationally engineered nanostructures remains a major hurdle against the development of functional peptide‐based nanomaterials. Various non‐covalent interactions come into play to drive the formation and stabilization of these assemblies, of which electrostatic interactions are key. Here, the atomistic mechanisms by which electrostatic interactions contribute toward controlling self‐assembly and lateral association of ultrashort β‐sheet forming peptides are deciphered. Our results show that this is governed by charge distribution and ionic complementarity, both affecting the interaction patterns between charged residues: terminal, core, and/or terminal‐to‐core attraction/repulsion. Controlling electrostatic interactions enabled fine‐tuning nanofiber morphology for the 16 examined peptides, resulting into versatile nanostructures ranging from extended thin fibrils and thick bundles to twisted helical “braids” and short pseudocrystalline nanosheets. This in turn affected the physical appearance and viscoelasticity of the formed materials, varying from turbid colloidal dispersions and viscous solutions to soft and stiff self‐supportive hydrogels, as revealed from oscillatory rheology. Atomistic mechanisms of electrostatic interaction patterns were confirmed by molecular dynamic simulations, validating molecular and nanoscopic characterization of the developed materials. In essence, detailed mechanisms of electrostatic interactions emphasizing the impact of charge distribution and ionic complementarity on self‐assembly, nanostructure formation, and hydrogelation are reported.

## Graphical abstract

Guiding molecular assembly of peptides remains a major hurdle against the development of rationally designed nanomaterials. This study unveils how electrostatic interactions govern self‐assembly and lateral association of β‐sheet nanofibers. Charge distribution and ionic complementarity are key factors shaping nanofiber morphology and hydrogelation. These insights are essential for fine‐tuning peptide nanomaterials’ properties and understanding the electrostatic mechanisms underlying protein fibrillation.

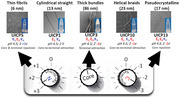

## 1 Introduction

Molecular self‐assembly is a natural phenomenon in which molecules spontaneously organize themselves into higher nanostructures, mainly via non‐covalent interactions.^[^ ^1^ ^]^ Short peptides have recently attracted great attention as highly functional molecules with natural propensity towards self‐assembly; an attribute that has been exploited to design versatile nanostructures for a wide variety of applications such as: tissue engineering and regenerative medicine,^[^ ^2^, ^3^, ^4^, ^5^, ^6^, ^7^, ^8^ ^]^ drug delivery,^[^ ^9^, ^10^, ^11^, ^12^, ^13^, ^14^ ^]^ antimicrobial materials,^[^ ^15^, ^16^, ^17^, ^18^, ^19^, ^20^, ^21^ ^]^ disease modeling,^[^ ^22^, ^23^ ^]^ biosensing,^[^ ^24^, ^25^ ^]^ biocatalyst supports,^[^ ^26^ ^]^ haemostatic agents,^[^ ^27^ ^]^ water treatment,^[^ ^28^, ^29^ ^]^ energy harvesting and electroconductivity,^[^ ^30^, ^31^, ^32^, ^33^ ^]^ and more.

One of the main unresolved challenges for the development of peptide‐based nanostructures is the uncontrolled molecular growth of peptide assemblies, and in particular β‐sheet formation^[^ ^34^ ^]^ There have been many successful endeavors to control and guide self‐assembly of β‐sheet forming peptides via manipulating hydrophobic interactions,^[^ ^35^, ^36^, ^37^, ^38^ ^]^ aromatic stacking,^[^ ^36^, ^39^, ^40^, ^41^, ^42^ ^]^ and chirality.^[^ ^34^, ^43^, ^44^, ^45^, ^46^, ^47^ ^]^ However, there is still much to be done to unravel the role of electrostatic interactions in controlling peptide secondary structure formation and the growth into nanoscale assemblies. The influence of charge type, density, and distribution on peptide self‐assembly cannot be easily predicted and might either stabilize or hamper building the assembled structure, depending on the local electrostatic environment.

**Scheme 1:**
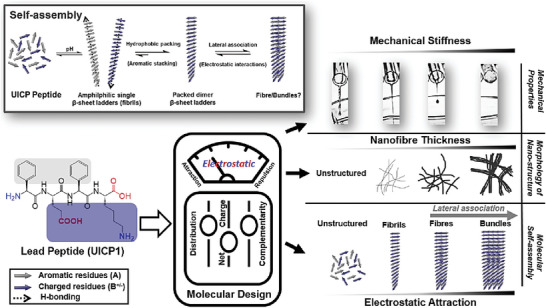
Depiction of UICP self‐assembly into β‐sheets and the effect of charge interaction on lateral association of these structures. Rational design of UICPs through fine‐tuning peptide net charge, charge distribution, and ionic self‐complementarity enables control over molecular self‐assembly, morphology, and size of nanofibrous structures formation, as well as mechanical properties of UICP hydrogels.

Ionic self‐complementary sequence pattern, with alternating hydrophobic (A) and charged (B^+/−^) residues (i.e., (AB^+/−^AB^+/−^)*_n_*, where n is the number of segments) is one of the β‐sheet forming peptide classes that are most affected by electrostatic interactions (**Scheme** **1**). In principle, masking electrostatic charges of B^+/‐^ residues is thought to reduce electrostatic repulsion between peptide chains and drives self‐assembly through hydrophobic interactions, van der Waals, and hydrogen bonding between peptide backbone amides.^[^ ^39^, ^48^, ^49^ ^]^ Indeed, pH plays a crucial role in guiding molecular self‐assembly into different nanostructures, since it controls the charge modulus of peptide chains. For instance, self‐assembly of the ionic self‐complementary peptide FEFEFKFK resulted in the formation of different nanofibre morphologies and hydrogels with different mechanical properties at low (<6) and high (>8) pH values.^[^ ^50^ ^]^ Similarly, different nanofibre sizes were obtained from the self‐assembly of Nap‐FEFK at different pH values due to variation in charge status, where at extreme acidic (pH 2) and basic (pH 12) values the peptide chains are charged leading to the formation of thin fibers due to electrostatic repulsion, while neutralization of charge at pH 7 promoted the lateral association of β‐sheet assemblies forming thicker fibers.^[^ ^48^ ^]^ VER peptides also exhibited more lateral aggregation of nanofibres with decreasing charge modulus.^[^ ^51^ ^]^

Besides charge status, the distribution of B^+/‐^ charged residues within the peptide sequence has been shown to significantly affect both the secondary structure and morphology of nano‐assemblies. Different charge distribution patterns of EAK16 ionic self‐complementary sequences led to the formation of β‐turn structure in the case of EAK‐IV (− − − − + + + +), while self‐assembly into β‐sheet structures was predominant for both EAK16‐I (− +) and ‐II (− −  + +).^[^ ^52^ ^]^ Interestingly, EAK16‐II (− −  + +) and ‐IV (− − − − + + + +) form distinct nanostructures, with the earlier forming fibrillar assemblies while the later assembled into globular morphology.^[^ ^53^ ^]^ Also regardless of pH value, EAK16‐II (− −  + +) always assembles into fibrillar structure, however, EAK16‐IV (− − − − + + + +) is more pH‐dependent as it forms a globular structure at pH 6.5–7.5 with a structural transition to fibrillar morphology outside this range.^[^ ^52^ ^]^ Although ionic self‐complementary peptides are very well studied, being the first reported *de novo* β‐sheet forming peptides,^[^ ^54^, ^55^ ^]^ yet there is a lack in clear understanding of the role played by electrostatic interactions in governing self‐assembly and nanostructure formation at the atomistic level.

We have recently adopted a minimalistic molecular engineering approach to develop ultrashort ionic‐complementary constrained peptide (UICP) using four amino acid residues (Phg‐Glu‐Phg‐Lys; UICP1) (**Table** **1**) and demonstrated the importance of aromatic stacking between the rigid phenylglycine (Phg) residues, alongside hydrogen bonding between backbone amides, for stabilization of β‐sheet nanofibers formation and hydrogelation (Scheme 1).^[^ ^39^ ^]^ We hypothesize that charge interactions are also key for both molecular self‐assembly of UICP chains into β‐sheet ladders and the lateral growth of packed ladder dimers into higher hierarchical structures (Scheme 1). In light of this, herein we explore the atomistic mechanism of lateral association through charge interactions and how this could be manipulated for fine‐tuning molecular self‐assembly, morphology, and size of nanofibrous structures, as well as mechanical properties of UICP hydrogels. We have developed a library of 16 UICPs (4‐5 residues long) (Table 1) to explore the effect of sequence net charge, charge distribution, reversal of charge order, and ionic self‐complementarity on their propensity toward self‐assembly and gelation.

**Table 1:** Showing amino acid sequences, abbreviations, and characterization of UICPs.

Peptide Code	Peptide Sequence	Abbreviation	Net charge	% β‐sheet abundance^a)^,^c)^	CGC [mm]^a)^	pH range for hydrogelation	Storage modulus *G*′ at 30 mm [kPa]^a)^,^c)^	Morphology of fibers^a)^	Diameter [nm]^a)^		
									SAXS		TEM^c)^
									Radius of gyrations (R_σ_)	Diameter (D)	
UICP1	Phg‐Glu‐Phg‐Lys	Phg4	0	47.1 ± 2.4	37	3.5‐6.8	N/A (<CGC)	Extended straight cylindrical fibres	3.4 ± 0.2	9.7 ± 0.4 ^b)^	10.3 ± 2.5 (≈11–13)
UICP2	Phg‐Glu‐Phg‐Lys‐Glu	Phg4E	−1	71.5 ± 2.8	30	3‐4.8	11.3 ± 2.1	Extended straight cylindrical	6.9 ± 0.2 at lower q 3.2 ± 0.2 at higher q	29.2 ± 0.6	40 ± 1.6 (≈38–44)
UICP3	Glu‐Phg‐Glu‐Phg‐Lys	EPhg4	−1	81.6 ± 1.8	15	3‐6	32.9 ± 1.4	Thick branched bundles	3.5 ± 0.2	9.9 ± 0.2	86 ± 22.1 (≈48–146)
UICP4	Phg‐Glu‐Phg‐Lys‐Lys	Phg4K	+1	0	N/A	N/A	N/A	No fibre formation	N/A	N/A	N/A
UICP5	Lys‐Phg‐Glu‐Phg‐Lys	KPhg4	+1	30.6 ± 2	75	2.7‐5.7	N/A	Very thin entangled fibrils	2.0 ± 0.2	5.6 ± 0.2	6 ± 1.7 (≈1–10)
UICP6	Phg‐Lys‐Phg‐ Glu	(Phg4)^Rev^	0	49.1 ± 1.7	37	4‐7.6	N/A (<CGC)	Extended straight cylindrical fibres	3.3 ± 0.2	9.4 ± 0.2	33.7 ± 4.7 (≈23–40)
UICP 7	Phg‐Lys‐Phg‐Glu‐Lys	(Phg4)^Rev^K	+1	0	N/A	N/A	N/A	No fibre formation	N/A	N/A	N/A
UICP 8	Lys‐Phg‐Lys‐Phg‐Glu	K(Phg4)^Rev^	+1	50.4 ± 1.5	30	2.8‐8.4	0.02 ± 0.003	Thin nanofibres	1.2 ± 0.2	3.3 ± 0.2	16.1 ± 2 (≈11–19)
UICP 9	Phg‐Lys‐Phg‐ Glu‐Glu	(Phg4)^Rev^E	−1	46.7 ± 2.3	15	2.4‐5.5	4.9 ± 0.8	Extended straight cylindrical	2.4 ± 0.2	6.7 ± 0.2	13.1 ± 3.4 (≈7–23)
UICP 10	Glu‐Phg‐Lys‐Phg‐ Glu	E(Phg4)^Rev^	−1	54.1 ± 2	7.5	2.2‐7	3.7 ± 0.8	Twisted helical fibre bundles (braid‐like structures)	4.1 ± 0.2	11.6 ± 0.2	23.5 ± 2.9 (≈18–31)
UICP11	Phg‐Glu‐Phg‐Glu	(PhgE)_2_	−2	0	N/A	N/A	N/A	N/A	N/A	N/A	N/A
UICP12	Phg‐Lys‐Phg‐Lys	(PhgK)_2_	+2	0	N/A	N/A	N/A	N/A	N/A	N/A	N/A
UICP13	Phg‐Glu‐Phg‐Glu‐Lys	(PhgE)_2_K	−1	33.5 ± 1.6	N/A	N/A	N/A	Pseudocrystalline nanosheets	Not measured		17.4 ± 2.2 (≈11–22)
UICP14	Lys‐Phg‐Glu‐Phg‐Glu	K(PhgE)_2_	−1	68.7 ± 2.1	7.5	1.4‐8.2	4.9 ± 0.6	Thick extended cylindrical nanofibre bundles	12.5 ± 0.2 at lower q 2.7 ± 0.2 At higher q	Not analysed	36.6 ± 2.5 (≈30–42)
UICP15	Phg‐Lys‐Phg‐Lys‐Glu	(PhgK)_2_E	+1	0	N/A	N/A	N/A	N/A	N/A	N/A	N/A
UICP16	Glu‐Phg‐Lys‐Phg‐Lys	E(PhgK)_2_	+1	36.8 ± 2.8	30	2.3‐8.2	0.06 ± 0.001	Thin nanofibres	1.1 ± 0.2	3.0 ± 0.2	15.8 + 2.6 (≈10–21)

^a)^ Measurement done for UICP samples at pH 4.5;

^b)^ Reproduced from our previous work in reference^[^ ^39^ ^]^ by fitting of rod‐like thin fibre;

^c)^ Data presented as mean ± SD (n = 3). N/A: not applicable.

For these investigations, a variety of biophysical techniques were used to characterize UICP properties over the length scale. For instance, molecular self‐assembly into β‐sheet secondary structures was investigated using Attenuated Total Reflectance–Fourier Transform Infrared spectroscopy (ATR‐FTIR), and the ability of these structures to form stable nanofibres was assessed using Thioflavin T (ThT) fluorescence test. Nanoscopic characterization of nanofibre size and morphology was performed using Transmission Electron Microscopy (TEM) and Small‐Angle X‐ray Scattering (SAXS), while Scanning Electron Microscopy (SEM) was utilized to investigate nanofibrous network structure. Bulk material properties were examined using inverted vial test for macroscopic investigation of gelation properties and oscillatory shear rheology for studying viscoelastic properties. To rationalize the role of electrostatic interaction on the lateral association of β‐sheet ladders, we performed atomistic Molecular Dynamics (MD) simulations of hundreds of peptides in water solution.

This study clearly demonstrates the importance of understanding the atomistic mechanisms of electrostatic interactions involved in self‐assembly of short ionic self‐complementary β‐sheet forming peptides. This led to the disclosure of the associated molecular design rules, which could be manipulated to control nanofibre thickness and mechanical properties of the formed hydrogels, hence giving the flexibility for tailoring these systems to the application needs.

## 2 Results and Discussion

We have previously reported the tetrapeptide UICP1, also known as Phg4, as the shortest ionic self‐complementary peptide that can self‐assemble into stable β‐sheet nanofibres, forming hydrogels (Table 1).^[^ ^39^ ^]^ We have demonstrated that aromatic interactions of the constrained phenylglycine (Phg) rings are crucial for stabilization of β‐sheet formation via intra‐ and inter‐ladder π–π stacking.^[^ ^39^ ^]^ Additionally, ionic complementary electrostatic interactions were shown to support lateral growth of the packed β‐sheet ladder dimers. To further investigate the role played by charge interactions in guiding and stabilizing self‐assembly of UICPs, herein we extended our sequence design by an additional charged residue (i.e., pentapeptide). This provided the flexibility in designing versatile peptide chains to study the effect of charge distribution and ionic self‐complementarity on propensity toward self‐assembly (Table 1). We have followed a rational molecular design approach to cover the main sequence patterning of charge distribution (i.e., N‐to‐C, C‐to‐N, core and terminal distribution of both +ve and −ve charges).

### 2.1 Effect of Charge Distribution

**Figure 1:**
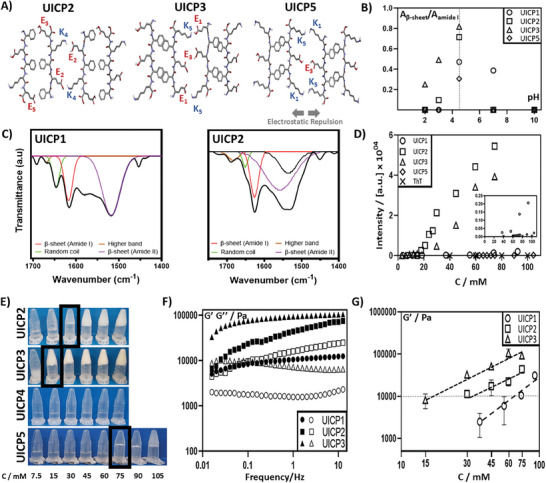
A) Schematic showing a top view for lateral association of two anti‐parallel packed β‐sheet dimers revealing the possible electrostatic interactions for peptides UICP2, 3, and 5. B) Relative β‐sheet peak area for peptides UICP1, 2, 3, and 5 over a range of different pH values (n = 3, mean ± SD). Highest relative area was observed for all peptides at pH 4.5, which is considered the optimal value for self‐assembly. C) ATR‐FTIR spectra peak deconvolution for amide I band of UICP1 and 2 at pH 4.5. D) ThT fluorescence shows significant enhancement of intensity for UICP2 and 3 as a function of concentration, indicating the high population of β‐sheet nanofibres. Enhancement of fluorescence was also observed for UICP1 but to a lower extent. An insignificant increase in fluorescence intensity was observed for UICP5 thanks to the very thin fibrils formation and the lower nanofibre population (n = 3, mean ± SD). E) Inverted vial test at pH 4.5 showing CGC of peptides UICP2, 3, and 5 as 30, 15, and 75 mm, respectively, while UICP4 did not form hydrogel at all the tested concentrations. F) Oscillatory rheology characterization showing frequency sweep measurements at strain (γ) 0.2%, within linear viscoelastic region, for peptides UICP1, 2, and 3 (close symbols: G′; open symbols: G″). G) Log–log plot of shear moduli (G′) versus molar concentrations obtained for peptides UICP1, 2, and 3. G′ values at 6 rad s^−1^ obtained from frequency sweep at 0.2% strain were used (n = 3, mean ± SD). Error bars in B, D and G in some cases are smaller than the data mark sizes.

The position of charged residues affects the overall charge distribution and propensity of the peptide sequence to self‐assembly.^[^ ^52^, ^53^ ^]^ To study this effect, we have first introduced a C‐terminal E residue to create Phg4E or UICP2 (Table 1 and **Figure** **1**A), which formed self‐supportive hydrogels at a pH range of ≈2.8–5 (Figure S1A, Supporting Information). The secondary structure of UICP2 was studied using ATR‐FTIR by investigating the characteristic peaks detected in the amide I (1600–1690 cm^−1^), amide II (1480–1575 cm^−1^), and amide III (1229–1301 cm^−1^) vibrational bands. The relative β‐sheet abundance at different pH values was calculated as the ratio of the deconvoluted β‐sheet peak area (at wavenumber 1611–1630 cm^−1^) relative to the total amide I band area, where the highest β‐sheet content was observed at pH 4.5 (Figure 1B and Figure S1B, Supporting Information). At this pH value, the overall net charge of UICP2 is ≈−1 (Table 1 and Figure S1C, Supporting Information), with both E and K residues charged, implying electrostatic attraction between countercharge sidechains of the core E_2_ and K_4_ residues (Figure 1A). Indeed, a prominent peak at 1628 cm^−1^ (amide I, C═O stretching) and a broad peak at ≈1530 cm^−1^ (amide II, C–N stretching, and N–H bending) were detected at pH 4.5, confirming the formation of an extended β‐sheet structure, with the 1690 cm^−1^ peak implying anti‐parallel arrangement (Figure 1C and Figure S1B, Supporting Information).^[^ ^56^, ^57^ ^]^ In addition, a small peak was detected at 1248 cm^−1^ in the amide III band, which could be assigned to the K residues side chain C–N stretching and N–H bending vibrations (Figure S1B, Characterization Table 1, and Characterization Figure C7, Supporting Information). A small peak of wavenumber 1649 cm^−1^ was also detected in the amide I band indicating the presence of a minor unstructured population (Figure 1C and Figure S1B, Supporting Information). Deconvolution of amide I band peaks revealed a higher relative β‐sheet content for UICP2 (72%) compared to UICP1 (47%), while the relative random population of UICP2 was 13%, which is lower than that estimated for UICP1 (37%) (Figure 1B,C and Table 1). These results corroborate the higher propensity of UICP2 toward β‐sheet formation than that of UICP1. This could be attributed to the additional C‐terminal E_5_, which is positioned in a preferable orientation at the hydrophobic face of the β‐sheet ladder for the anion‐π planar interaction to occur between the anionic ɣ‐carboxylate and the electropositive ring edge of the N‐terminal Phg residue (Figure 1A).^[^ ^58^, ^59^ ^]^ β‐sheet nanofibre content was also investigated by assessing ThT fluorescence, where the selective binding of ThT to β‐sheet fibers leads to enhanced emission at λ ≈485 nm.^[^ ^60^, ^61^ ^]^ The detected emission fluorescence intensity of UICP2 was significantly higher than that of UICP1 at all the tested peptide concentrations, thanks to the substantially higher β‐sheet nanofibre content of the earlier (Figure 1D).

**Figure 2:**
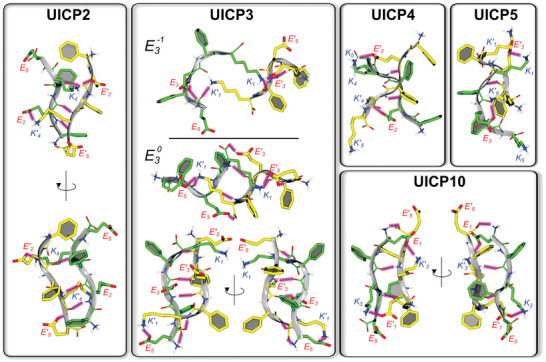
Representative peptide pairs found during 1 µs‐long all‐atom MD simulations of hundreds of UICP2, UICP3, UICP4, and UICP10 molecules in 0.1 m KCl water solution. Sidechain and backbone atoms are shown with thick and thin sticks respectively, colored by atom type (red: oxygen; blue: nitrogen; white: hydrogen; green and yellow: carbon atoms on opposite strands). H‐bonds are shown by black dashed arrows highlighted pink.

MD simulations were employed to assess the early steps of the self‐assembly process, which initially occurs through dimerization, as previously noted.^[^ ^62^, ^63^, ^64^, ^65^ ^]^ µs‐long MD simulations of hundreds of pentapeptides in 0.1 m KCl water solution revealed the presence of several tens of relatively stable anti‐parallel β‐strand pairs stabilized both by salt bridges between K_4_ and E_2_ amino acids on opposite strands, as well as by the formation of Phg stacks (**Figure** **2** and Table S2, Supporting Information). Segregation was observed, and the anion‐π planar interaction was also found sporadically in our MD simulations, although it competed with the formation of H‐bonds between K_4_ and E_5_ on opposite strands (Figure 2). Either way, this additional interaction led to enhancement of self‐assembly as manifested by the significant increase of both β‐sheet structure population (ATR‐FTIR; Figure 1B,C and Figure S1B, Supporting Information) and nanofibre content (ThT fluorescence; Figure 1D) of UICP2 as compared to UICP1.

Bulk material properties were also affected by this structural change, where inverted vial test revealed a CGC of 30 mm for UICP2 (Figure 1C and Table 1), which is slightly lower than that for UICP1 (CGC 37 mm, Table 1). Oscillatory rheology was used to study the viscoelastic properties of the developed hydrogels. Frequency sweeps were employed to assess the mechanical stiffness at 0.2% strain, a strain value at which hydrogels were within their linear viscoelastic behavior (Figure S2, Supporting Information). In essence, the shear moduli (G′) of UICPs showed a gradual linear increase as a function of molar concentration, with an overall significant enhancement of UICP2 hydrogel storage modulus G′ reaching > 10 kPa range for all tested concentrations, compared to UICP1 hydrogels which were generally <≈10 kPa (Figure 1F,G). UICP2 samples of concentrations < 30 mm did not possess mechanical characteristics of viscoelastic hydrogels when examined by oscillatory rheology (i.e., Tan δ >1 with G′ of low magnitude <100 Pa; data not shown), recapitulating the CGC observed from the inverted vial test as 30 mm (Figure 1E).

**Figure 3:**
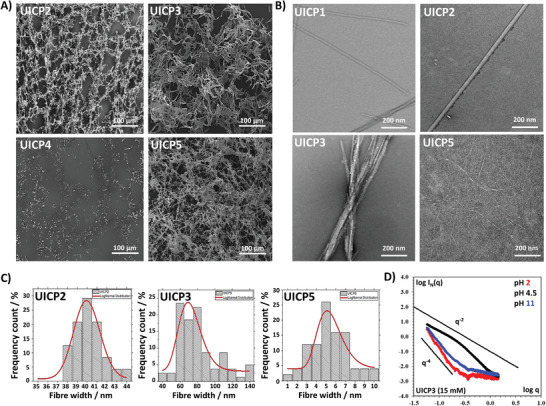
A) SEM micrographs of 45 mm UICP2, 3 and 4, and 75 mm UICP5 peptide samples (all 20× diluted) showing the continuous network of entangled nanofibres, with the exception of UICP4, which failed to self‐assemble into nanofibrous structures (Scale bar = 100 µm). B) TEM micrographs of 45 mm UICP1, 2 and 3 samples (10× diluted) showing peptide nanofibre bundles, while the 75 mm UICP5 sample (10× diluted) showed the formation of very thin fibrils (Scale bar = 200 nm). C) Histograms for size distribution for nanofibers of UICP2, 3, and 5 peptides measured from TEM micrographs, with average sizes of 40 ± 1.6, 86 ± 22.1, and 6 ± 1.7 nm (n = 100 ± SD), respectively. D) SAXS characterization of UICP3 prepared at different pH values in double logarithmic plot of I_N_(q) versus q representation. The straight lines depict type of slope for easier visualization.

Electron microscopy techniques were used to characterize hydrogels at mesoscopic scale. SEM was employed to scan network surface topography^[^ ^66^ ^]^ where micrographs showed the formation of continuous 3D networks of entangled nanofibrous structures for both peptides (**Figure** **3**A). According to Jones and Marques theory, which correlates network topology with the types of elastic deformation of the constituent fibers in hydrogels using Equations (1) and (2), the fractal dimension (D_F_) of the extended straight nanofibres forming the network is expected to be 1, i.e., a power law of *C^2^* for enthalpic elasticity dominated networks and *C^1.5^* for entropic elasticity dominated networks.*^[^* ^67^ ^]^ In good agreement with this theory, UICP1 had a power law of *C* ^2.6±0.4^ and UICP2 *C* ^1.4±0.2^, respectively (Figure 1G). This indicates that most of elasticity is retained in the rigidity of the straight fibers in the case of UICP1, whereas in the case of UICP2 it comes from the conformational rearrangement occurring at the junctions of the network.^[^ ^68^ ^]^

(1) $$G^{\prime }\propto C^{\left(3+DF\right)/\left(3-DF\right)}\;\mathrm{for}\;\mathrm{enthalpic}\;\mathrm{elasticity}\;\mathrm{dominated}\;\mathrm{networks}$$

(2) $$G^{\prime }\propto C^{\left(3\right)/\left(3-DF\right)}\;\mathrm{for}\;\mathrm{entropic}\;\mathrm{elasticity}\;\mathrm{dominated}\;\mathrm{networks}$$

where *C* is the concentration of nanofibres contributing to the network formation and elasticity.

The different elastic behavior of both peptides was also observed in amplitude sweep measurements. The softer UICP1 hydrogel was able to withstand the increasing strains starting from 0.01% and up to 10%, whereas stiffer UICP2 hydrogel showed transitioning towards liquid state around ≈1% strain and reached a crossover point of *G′/ G″* at ≈10% strain (Figure S2A, Supporting Information).

The morphology of the formed nanofibres structure was resolved by the high‐resolution TEM and SAXS.^[^ ^66^ ^]^ TEM micrographs revealed the ability of both peptides to form extended straight β‐sheet nanofibrillar structures, with UICP1 forming structures of 10.3 ± 2.5 nm diameter, while UICP2 formed thicker ≈40 ± 1.6 nm structures with thickness range of 38–44 nm, due to the additional stabilization of lateral growth via the terminal E_5_‐Phg anion‐π interaction (Figures 1A and 3B,C, and Table 1). The UICP1 sizes compare well to those we obtained previously as measured by SAXS with d = 9.7 ± 0.4 nm (Table 1).^[^ ^39^ ^]^ We have also measured SAXS of UICP2 and confirmed the tendency for bundling and thicker structures, with a higher radius of gyration obtained *R*_σ _= 6.9 ± 0.2 for UICP2, as compared to *R*_σ _= 3.4 ± 0.2 for original UICP1 (Table 1). This corresponds by rough estimations of the size in the range 29.2 ± 0.6 nm, confirming visual TEM observations.

The introduction of an additional E residue at the N‐terminus, on the other hand, led to the formation of EPhg4 or UICP3 in which the peptide sequence is flanked by the E_1_ anionic residue and K_5_ cationic one (Table 1 and Figure 1A). These exposed charged terminal residues of UICP3 provided better conformation for anti‐parallel lateral growth via the readily accessible countercharge electrostatic attraction, when compared to the interactions between the core‐charged residues of both UICP1 and 2 (Figure 1A). Moreover, better orientation for π–π interactions between the inner core Phg residues can be envisaged for UICP3 with less steric hinderance at the hydrophobic face of β‐sheet ladder, where no other sidechains exist (Figure 1A).^[^ ^39^, ^58^, ^69^, ^70^ ^]^ Strikingly, this design led to self‐assembly of UICP3 into stable β‐sheet structures, as revealed from the analysis of FTIR amide I (prominent peak at 1626 cm^−1^, C═O stretching), amide II (broad peak at ≈1535 cm^−1^, C─N stretching and N─H bending), and amide III (small peak at 1238 cm^−1^, C–N stretching and N–H bending) bands (Figure S1B, Characterization Table 1, and Characterization Figure C11, Supporting Information). The highest β‐sheet abundance of ≈82% was achieved at pH 4.5 (Figure 1B). Like UICP2, UICP3 showed a significant enhancement in ThT fluorescence compared to UICP1, implying higher β‐sheet fiber content (Figure 1D). TEM showed the formation of thick branched fiber bundles of 86 ± 22.1 nm diameter (size range 48–146 nm), thanks to the lateral associations via countercharge electrostatic attraction along the fibers’ length (Figure 3B,C). We then confirmed the largest abundance of β‐sheet structures and hydrogelation at pH 4.5 by SAXS measurements (Figure 3D). UICP3 at pH 4.5 followed q^−2^ behavior, often associated with flat ribbons, lamella, or disc‐shaped particles.^[^ ^71^, ^72^ ^]^ At pH values 2 or 11, the curves shifted to follow q^−4^ behavior, indicating aggregated globules with a nearly smooth surface.^[^ ^66^, ^73^ ^]^ The same “folded” nature was observed in Kratky representation (Figure S3A, Supporting Information), indicating a propensity for structured self‐assembly at pH 4.5 for UICP3. SAXS analysis at low q indicated R_σ_ = 3.5 ± 0.2, which by fitting rod‐like model gave a diameter of 9.9 ± 0.2 nm (Table 1), roughly fitting basic fibrillar units similar to UICP1. SEM showed that the thick‐branched UICP3 bundles entangled to form dense networks (Figure 3A), with hydrogel formation at a CGC 15 mm, which is half the concentration needed for UICP2 hydrogelation (CGC 30 mm) (Table 1 and Figure 1E). It can also be observed that below CGC, UICP3 peptide formed a cloudy dispersion due to the thick fiber bundles formation (Figure 1E). UICP3 hydrogels were significantly stiffer than both UICP1 and 2 at all examined concentrations, reaching a G′ ≥ ≈100 kPa at 60 and 75 mm, and exhibited entropic elasticity dominated networks behavior with a power law of *C* 1.6 ± 0.3, confirming the network junction and their deformation as key contributors to the hydrogel viscoelasticity (Figure 1F,G).

As for UICP2, the dynamics of the interaction among hundreds UICP3 peptides was investigated by means of MD simulations. K_5_ was found to interact either with E_1_ (as expected by design) or with E_3_ and/or the backbone carboxyl groups (Figure 2). The elongated K_5_ sidechains promoting salt bridges between UICP3 pairs expose the Phg sidechains of different peptides towards opposite sides, in contrast with the situation seen for UICP2; thus, supramolecular structure formation should rely on the onset of hydrophobic interactions with additional peptides along the direction contained in the plane of the dimer and perpendicular to the line joining the N‐ and C‐terminus. Since supramolecular structures are formed at pH 3–6 and the p*K*a of the central E sidechain is ≈4.3, we assessed the role of the protonation state of E_3_ by performing simulations also with protonated sidechain for this amino acid. The results show that, as expected, such protonation enhances the formation of anti‐parallel β‐sheet pairs whereby hydrophobic/hydrophilic segregation is mediated by the stacking of the Phg residues on the same side of the β‐sheet, as seen for UICP2 (Figure 2).

Conversely, the cationic counterparts UICP4 (Phg4K, +1) and UICP5 (KPhg4, +1) behaved differently at pH 4.5 (Table 1, Figure 1A, and Figure S1C, Supporting Information). The UICP4 sequence neither self‐assembled into β‐sheet structures as revealed from FTIR spectrum (absence of β‐sheet peak with the presence of prominent peaks at 1650 cm^−1^ and 1676 cm^−1^, and a small peak at 1258 cm^−1^, indicating unordered structure) (Figure S1B, Characterization Table 1, and Characterization Figure C15, Supporting Information) nor did it form nanofibers as observed from SEM (Figure 3A). Consequently, it failed to form self‐supportive hydrogels at a concentration range of 7.5–75 mm (Figure 1E). This is attributed to the electrostatic repulsion caused by the core anionic E_2_, as well as the exposed C‐terminal K_4_ and K_5_ cationic residues. In addition, the presence of K_5_ residue at the hydrophobic side could have caused steric hindrance between peptide chains, making it difficult for π–π interaction to happen between core Phg residues (Figure S4C, Supporting Information). Again, these hypotheses are confirmed by MD simulations, showing that in contrast to UICP2/3, peptide dimers can assume in UICP4 a cross‐like shape mostly stabilized by the formation of salt bridges between E_2_ on one strand and K_4_ on the other strand (Figure 2). These results point to a structural impairment affecting the self‐assembly of this peptide. Indeed, the addition of a second K residue could induce a significant imbalance in the intermolecular forces between different peptides. This in turn should limit the number of conformations enabling even partial stacking between Phg residues, affecting the propensity for association of large numbers of peptide chains.

While in the case of UICP5, both inner core Phg residues are in a good orientation for π‐π stacking without steric hindrance from the sidechain of any other charged residue (Table 1 and Figure 1A). Thus, efficient packing of β‐sheet ladders was achieved via aromatic interactions, shielding the hydrophobic faces from the surrounding aqueous medium. On the flip side, strong electrostatic repulsion is expected from the similar charge residues (K_1_‐K_5_, E_3_‐E_3_, and K_5_‐K_1_) of the exposed hydrophilic face of β‐sheet ladders, preventing lateral growth (Figure 1A). In our MD simulations, we detected many pairs of UICP5 peptides assuming a shifted anti‐parallel conformation stabilized by K_1_‐E_3_ salt bridges and Phg stacking (Figure 2 and Table S2, Supporting Information). However, the conformational strain imposed by this structure largely impaired the formation of H‐bonds between CO and NH groups on opposite strands, contrary to UICP2 and UICP3 (particularly with neutral E_3_ sidechains in the latter peptide). Consistently with the altered (reduced) interaction between peptide strands, UICP5 formed very thin entangled fibrils with Rσ = 2.0 ± 0.2 (and calculated diameter of 5.6 ± 0.2 nm) from SAXS measurements (Table 1) and diameter 6 ± 1.7 nm with size range of 1–10 nm taken from TEM (Figure 3A–C), with a very low 30% β‐sheet structure population and insignificant nanofibre content, as revealed from FTIR and ThT fluorescence spectra analysis, respectively (Figure 1B,D, and Figures S1B and S2A, Supporting Information). Besides, UICP5 failed to form hydrogels, but rather formed viscous soft materials at very high concentrations (≥75 mm) (Figure 1E), which did not follow a typical linear viscoelastic behavior, as revealed from amplitude sweep measurements (Figure S2A, Supporting Information). These results agree with the findings reported by Saiani and co‐workers, who showed that the addition of two terminal K residues, one at each side of the ionic self‐complementary octapeptide sequence F8 (i.e., **K**FEFKFEFK**K**), significantly reduced tendency for lateral association and formation of large fiber bundles and consequently resulted in the formation of hydrogels with lower storage modulus compared to F8 (FEFKFEFK).^[^ ^68^ ^]^

### 2.2 Reversal of Charge

**Figure 4:**
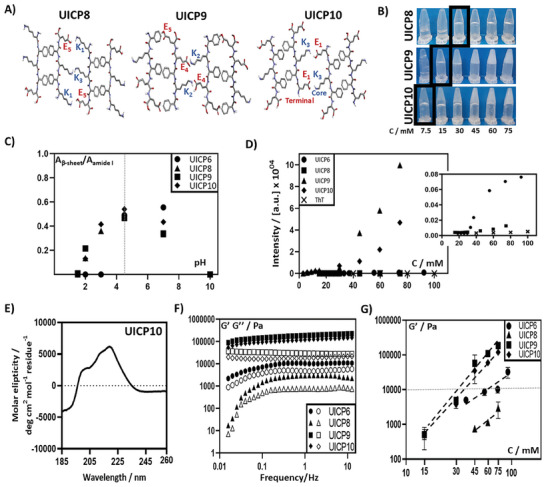
A) Schematic showing a top view for lateral association of two anti‐parallel packed β‐sheet dimers revealing the possible electrostatic interactions for peptides UICP8, 9, and 10. B) Inverted vial test at pH 4.5 showing CGC of peptides UICP8, 9, and 10 as 30, 15, and 7.5 mm, respectively. C) Relative β‐sheet peak area for peptides UICP6, 8, 9, and 10 over a range of different pH values (n = 3, mean ± SD). The highest relative area was observed for all peptides at pH 4.5, which is considered the optimal value for self‐assembly. D) ThT fluorescence shows a significant enhancement of intensity for UICP9 and 10 as a function of concentration, indicating the high population of β‐sheet nanofibres. Enhancement of fluorescence was also observed for UICP6 and 8 but to a lower extent (n = 3, mean ± SD). E) CD spectrum of UICP10 showing positive peaks at 220 nm and 205 nm and a negative peak at 193 nm akin to the CD profile of triple helix collagen‐like peptides. F) Oscillatory rheology characterization showing frequency sweep measurements at strain (γ) 0.2%, within the linear viscoelastic region, for peptides UICP6, 8, 9, and 10 (close symbols: G′; open symbols: G″). G) Log‐log plot of shear moduli (G′) versus molar concentrations obtained for peptides UICP6, 8, 9, and 10. G′ values at 6 rad s^−1^ obtained from frequency sweep at 0.2% strain were used (n = 3, mean ± SD). Error bars in C, D and G in some cases are smaller than the data mark sizes.

**Figure 5:**
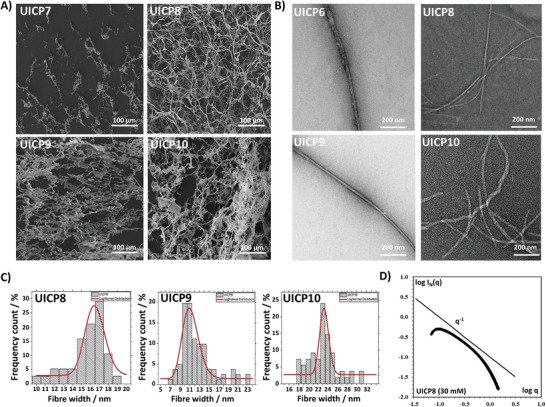
A) SEM micrographs of 45 mM UICP7‐10 peptide samples (all 20× diluted) showing the continuous network of entangled nanofibres, with the exception of UICP7, which showed amorphous peptide aggregates as it failed to self‐assemble into nanofibrous structures (Scale bar = 100 µm). B) TEM micrographs of 45 mm UICP6, 8, 9, and 10 samples (10× diluted), showing peptide nanofibre bundles. The formation of the unique twisted helical/braid‐like structures can be observed for UICP10 (Scale bar = 200 nm). F) Histograms for size distribution for nanofibers of UICP8, 9 and 10 peptides measured from TEM micrographs, with an average sizes of 16.1 ± 2, 13.1 ± 3.4 and 23.5 ± 2.9 nm (n = 100 ± SD), respectively. C) Histograms for size distribution for nanofibers of UICP8, 9 and 10 peptides measured from TEM micrographs, with an average sizes of 16.1 ± 2, 13.1 ± 3.4, and 23.5 ± 2.9 nm (n = 100 ± SD), respectively. D) SAXS characterization of UICP8 prepared at 30 mM concentration in double logarithmic plot of I_N_(q) versus q representation. The straight line depicts type of slope for easier visualization.

We then investigated the effect of the reversal of charged residues order on self‐assembly and gelation. In doing this, we first designed the tetrapeptide UICP6 (Phg‐Lys‐Phg‐Glu), the reversed charge counterpart of UICP1 (Phg4), which we called Phg4^Rev^ (Table 1). With a net neutral charge at pH 4.5 for both peptides, UICP6 exhibited closely similar behavior to UICP1, forming hydrogels with same CGC of 37 mm, similar phase diagram (Figure S4A, Supporting Information), secondary structure profile (49% β‐sheet and 32% random population) (**Figures** **4**C and S4B, Supporting Information), nanofiber content (Figure 4D) and rheological properties (Figure 4F,G).^[^ ^39^ ^]^ Likewise, the UICP6 fiber morphology observed by TEM was similar to UICP1, both adopting extended straight β‐sheet rod‐like structures, with slightly thicker diameter 33.7 ± 4.7 nm of size range ≈23–40 nm for UICP6 (**Figure** **5**B and Table 1). SAXS curve for UICP6 at pH 4.5 followed q^−1^ behavior (Figure S3B, Supporting Information) at low q and yielded similar *R*_σ _= 3.3 ± 0.2 nm/diameter of 9.4 ± 0.2 nm values of constituent fibers, similarly to UICP1 (Table 1). For UICP6 at pH 11, the curves again shifted to follow q^−4^ behavior at low q, indicating aggregated globules with a nearly smooth surface^[^ ^66^, ^73^ ^]^ (Figure S3B, Supporting Information).

In contrast, the cationic UICP7 or (Phg4)^Rev^K showed a dramatic alteration of molecular behavior as compared to the mirroring anionic counterpart UICP2 (Phg4E) (Table 1). While UICP2 showed a good propensity towards self‐assembly and gelation as discussed above, UICP7 totally failed to self‐assemble into β‐sheet structures as evidenced by the absence of β‐sheet peaks in the FTIR spectrum and the presence of unordered structure peaks at 1677 cm^−1^ (C═O stretching, amide I) and 1254 cm^−1^ (C–N stretching and N–H bending, amide III) (Figure S4B, Characterization Table 1, and Characterization Figure C26, Supporting Information). In addition, UICP7 neither formed nanofibres (SEM, Figure 5A) nor hydrogels at all tested concentrations at pH 4.5 (data not shown). Interestingly, UICP7 shared a similar structural feature with UICP4, both flanked with a Phg residue at the N‐terminus and a K_5_ at C‐terminus, and both peptides did not show any tendency to neither self‐assembly nor hydrogelation (Table 1). This is caused by the steric effect of the bulky K_5_ side chain at the hydrophobic face, which hindered occurrence of the possible aromatic interactions between core Phg residues (Figure S4C, Supporting Information).

On the other hand, moving K residue from C‐ to N‐ terminus to create UICP8 (also known as K(Phg4)^Rev^), improved the ability of the peptide chain to self‐assemble into β‐sheet structures with the highest β‐sheet abundance ≈50% achieved at pH 4.5 (Figure 4C and Figure S4B, Supporting Information). Besides the anti‐parallel β‐sheet structure shown in FTIR spectrum (1623 cm^−1^ and 1680 cm^−1^), a minor 17% of random coil population was also detected (1650 cm^−1^) (Figure S4B, Characterization Table 1, and Characterization Figure C30, Supporting Information). At N‐terminus, K_1_ is placed on the hydrophilic face of the β‐sheet ladder and is readily exposed to electrostatic attraction with the countercharge C‐terminus E_5_ permitting lateral growth (Figure 4A). Moreover, on the hydrophobic side, the two pairs of core Phg residues are positioned in better proximity favoring aromatic packing via π–π interactions (Figure 4A).^[^ ^39^ ^]^

Interestingly, UICP8 was able to self‐assemble and form stable hydrogels in slightly basic media, as it showed a wider gelation pH range of 2.8–8.4 compared to other UICPs (Table 1 and Figure S4A, Supporting Information). At basic pH, there is a risk of racemization of the aromatic *L*‐Phg residues (positions P2 and P4), upon the deprotonation of the acidic hydrogen at the benzylic α‐carbon, followed by re‐protonation of the formed carbanion, which could result in the formation of 3 diastereoisomers: *L* ***D_2_*** *L* ***L_4_*** *L*, *L* ***L_2_*** *L* ***D_4_*** *L*, and/or *L* ***D_2_*** *L* ***D_4_*** *L*.^[^ ^74^ ^]^ If this happened, this racemization could significantly impair the aromatic packing, hence the stability of self‐assembly, and could change the morphology of nanostructures and the viscoelasticity of hydrogels.^[^ ^43^ ^]^ Therefore, the purity of UICP8 was checked by RP‐HPLC before hydrogelation at pH 2 and after hydrogelation at pH 4.5, 7, and 8. All chromatograms revealed a single peak at the same retention time (2.12 min.) at all pH values, ruling out the presence of diastereoisomers (Figure S5, Supporting Information). Similarly, no racemization of *L*‐Phg residues was observed in the case of UICP14 and UICP16, for the hydrogels formed in slightly basic pH media (data not shown).

Unlike its anionic counterpart UICP3 (EPhg4) which formed opaque hydrogels with CGC 15 mm (Figure 1E), UICP8 formed translucent hydrogels with a twofold higher CGC of 30 mm concentration at pH 4.5 (Figure 4B). Furthermore, oscillatory rheology showed that UICP8 formed softer hydrogels with storage modulus of lower G′ moduli <10 kPa (Figure 4F,G), compared to UICP3 hydrogels that possessed significantly higher storage moduli G′ >10 kPa (Figure 1F,G), for same molar concentrations of both peptides. The noticeable variations in hydrogel physical appearance and G′ are ascribed to the significant difference in the morphology and size of the nanofiber bundles formed by the two peptides. Indeed, the stiff opaque UICP3 hydrogels are a result of dense entanglement of thick branched fiber bundles of 86 ± 22.1 nm diameter (Figure 3A–C), while the softer translucent UICP8 hydrogels are formed by the entanglement of the much thinner 16.1 ± 2 nm diameter fibers with size range 11–19 nm (Figure 5A–C). SAXS analysis of UICP8 at 30 mm and pH 4.5 showed that it followed q^−1^ behavior with some aggregation noted at very low q values (Figure 5D). By fitting straight rod model, *R*_σ _= 1.1 ± 0.2 nm and similar *d* = 3.0 ± 0.2 nm were obtained, on pair with UICP8, giving thinnest nanofibres obtained across all tested UICPs (Table 1). This correlated well with the obtained TEM (Figure 5B,C) and followed the observed trend in the smallest sizes observed in TEM. Additionally, for UICP8 a power law of *C* ^2.6±0.4^ was obtained (Figure 4G), which is of slightly higher exponent value than UICP3 (*C* ^1.6±03^, Figure 1G) indicating the presence of a high number of fibers that are not connected to the network.^[^ ^38^, ^75^ ^]^

As noted above, this again indicates that for UICP8 most of the elasticity is retained in the rigidity of the straight fibers, whereas for UICP3 it comes from deformation originating from the network junctions. These differences in material viscoelastic properties are directly related to differences in nanofibre sizes and junction‐forming capacity due to the discrete tendencies toward hydrophilic lateral growth for both peptides. Although in both peptides lateral growth is stabilized through electrostatic attraction between terminal countercharge residues, UICP8 showed less propagation of this lateral growth, which could be a result of electrostatic repulsion between the core K_3_ residues (Figure 4A), hence thinner fibers formation (Figure 5A–C), very low nanofiber content (ThT fluorescence, Figure 4D) and the presence of random structures due to the partially non‐assembled/disassembled peptides (Figure S4B, Supporting Information). This happens to a lesser extent in UICP3 with less electrostatic repulsion is expected between the shorter E_3_ residues sidechains, thus favoring lateral growth resulting in significantly thicker fiber bundles (Figures 1A, and 3B,C) and significantly higher nanofibre content (ThT fluorescence, Figure 1D).

The UICP9 or (Phg4)^Rev^E peptide was designed as the anionic countercharge analog of the cationic peptide UICP4 (Phg4K) (Table 1 and Figure 4A). As discussed earlier, UICP4 did not form β‐structure but rather adopted random conformation (Supplementary Figure S1B), while UICP9 conversely, self‐assembled into anti‐parallel β‐sheet structure as revealed from FTIR peaks analysis from amide I (1624 cm^−1^ and 1689 cm^−1^, C═O stretching) and amide III (1243 cm^−1^, C–N stretching, N–H bending) bands (Figure 4C, Figure S4B, Characterization Table 1, and Characterization Figure C34, Supporting Information). Although the higher abundance of the β‐sheet population (≈47%), a lower population of 25% unordered structure was also observed (1650 cm^−1^, amide I) (Table 1 and Figure S4B, Supporting Information). UICP9 formed extended cylindrical β‐sheet nanofibrous structures with R_σ _= 2.4 ± 0.2 nm/diameter of 6.7 ± 0.2 nm from SAXS and of diameter size 13.1 ± 3.4 nm from TEM (Table 1, Figure 5B,C), with very high nanofibre content (ThT fluorescence, Figure 4D). Strikingly, UICP9 fiber morphology and content are akin to UICP2 (Figure 1D and Figure 3B), where both peptides share the same interaction patterns on both the hydrophobic and hydrophilic sides of the β‐sheet ladder. These included hydrophobic packing via aromatic interactions between core Phg residues augmented with terminal E_5_‐Phg anion‐π interaction, alongside lateral growth through the electrostatic attraction between core countercharge residue pairs at the hydrophilic side (Figures 1A and 4A). Despite the molecular and morphological similarity of nanofibrous structures, UICP9 formed slightly stiffer hydrogels than UICP2 (Figure 1G and Figure 4G) with a CGC 15 mm (Figure 4B), which is half that of UICP2 (30 mm) (Figure 1E).

Interestingly, moving the E residue to N‐terminus led UICP10 (EPhg4^Rev^) sequence to form twisted helical fibres (R_σ _= 4.1 ± 0.2 nm/diameter of 11.6 ± 0.2 nm from SAXS and diameter 23.5 ± 2.9 nm from TEM), which are distinct from the UICP9 cylindrical fibre morphology (Table 1, Figure 5B,C). The unique twisted “braid” like morphology arises from the different lateral growth pattern of UICP10, which propagates via the “core‐to‐terminal” countercharge attraction between K_3_ and E_1_, respectively (Figure 4A). MD simulations confirmed this hypothesis and provided interesting hints on the molecular interactions arising between two peptide chains. UICP10 was also able to form tens of relatively stable anti‐parallel β‐sheet pairs (Table S2, Supporting Information), and among the dynamical network of interactions sampled during the simulation, that between K_3_ and E_1_ was detected often (Figure 2 and Table S2, Supporting Information). Importantly, this interaction occurred without the need for a significant shift of the peptides along their main direction (as seen for UICP5), thus enabling further stabilization through a dense network of inter‐backbone H‐bonds (that includes the carboxyl and amino termini).

Indeed, UICP10 β‐sheet secondary structure was confirmed by ATR‐FTIR, where anti‐parallel β‐sheet peaks were detected in amide I (1625 cm^−1^ and 1691 cm^−1^), amide II (broad peak at ∼1525 cm^−1^) and amide III bands (1248 cm^−1^) (Figure 4C, Figure S4B, Characterization Table 1, and Characterization Figure C38, Supporting Information), with an estimate of 54% relative β‐sheet content and absence of random structure population (Table 1). Circular Dichroism (CD) was used to further investigate UICP10 structure, and unexpectedly, ani‐parallel β‐sheet formation was not detected, but rather a strong positive peak at 220 nm and another peak at 205 nm, with a negative peak appearing at 193 nm (Figure 4E). This profile is akin to the CD of the triple helix structure detected for collagen‐like peptides, confirming the twisted helical braid morphology of UICP10 observed from the TEM micrographs (Figure 5B).^[^ ^76^, ^77^ ^]^ It is also notable that UICP10 twisted bundles showed close morphological similarity to the twisted amyloid structures of both L‐ and D‐Aβ(16–22) sequences that were reported by Nilsson and co‐workers to form pleated β‐sheet secondary structures.^[^ ^78^ ^]^

Entanglement of the UICP10 twisted bundles (SEM micrograph, Figure 5A) stabilized the formation of self‐supportive hydrogel of very low CGC ≤ 7.5 mm (Figure 4B). Rheological frequency sweeps data analysis showed that UICP10 hydrogels exhibited a wide range of stiffness with storage moduli G′ values spanning from ≈100 Pa at 7.5 mm concentration and up to ≈120 kPa for 75 mm (Figure 4G). Although both UICP9 and UICP10 possess obvious differences in fiber morphology (Figure 5B), it is notable that they exhibited a power law of similar exponent values, which are *C* ^3.8±0.2^ and *C* ^3.5±0.3^, respectively, suggesting that they are likely to have similar network elastic properties (Figure 4G and Figure S2B, Supporting Information). These are significantly higher than exponent values for other UICPs, indicating the presence of more pendent fibers that are not contributing to the network structure,^[^ ^38^, ^75^ ^]^ thanks to the high polydispersity of fibers formed by both peptides (Figure 5C). These exponents also provide basis for a very different topology of networks and retainment of elasticity as compared to the previously discussed UICPs. Putatively, the elasticity in UICP10 can stem from the possible additional deformations relating to the screw‐like motions in nanofibers during rheological measurements, as evidenced by their unique structural screw‐like/helical arrangement in TEM (Figure 5B). The same value in UICP9 cannot be ascribed to the evidenced data, hence future studies would be required to evaluate these, which are out of the scope of the current manuscript.

### 2.3 Ionic Self‐Complementarity

In addition to charge distribution, we have investigated the importance of ionic self‐complementarity of peptide sequence for β‐sheet structure formation and lateral association. As expected, tetrapeptide sequences formed of either all negative (UICP11; (PhgE)_2_) or all positive (UICP12; (PhgK)_2_) charged residues failed to form β‐sheet structures over a pH range of 1.5–10 (data not shown), including pH 4.5 (Characterization Table 1 and Characterization Figures C42 and C46, Supporting Information), suggesting that aromatic interactions between Phg residues per se are insufficient to overcome electrostatic repulsion and failed to stabilize self‐assembly (Table 1). We have then extended the sequence with an additional countercharge residue to examine whether this structural modification will recover ionic self‐complementarity and tendency to self‐assembly.

**Figure 6:**
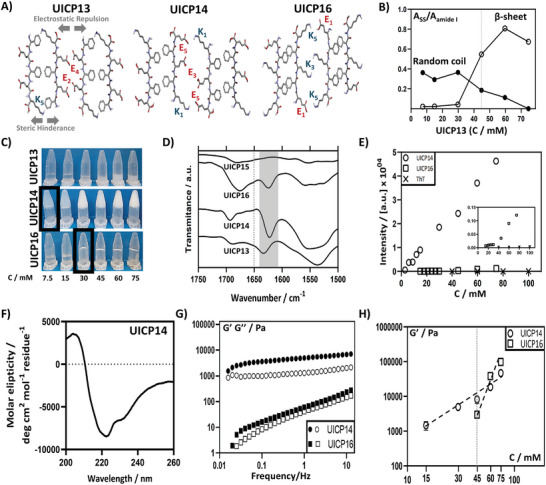
A) Schematic showing a top view for the lateral association of two anti‐parallel packed β‐sheet dimers revealing the possible electrostatic interactions for peptides UICP13, 14, and 16. B) Increase in relative β‐sheet population and reduction of random coils of UICP13 as a function of peptide concentration (n = 3, mean ± SD). C) Inverted vial test at pH 4.5 showing CGC of peptides UICP14 and 16 as 7.5 and 30 mm, respectively, while UICP3 did not form hydrogel at all the tested concentrations with the formation turbid colloidal dispersions at concentrations ≥ 45 mM. D) ATR‐FTIR spectra for UICP13‐16 at 75 mM showing β‐sheet peaks for all peptides (1611 cm^−1^ to 1630 cm^−1^, shaded in grey) except for UICP15, which was unstructured. UICP13 and 14 showed a prominent peak at ≈1690 cm^−1^ indicating anti‐parallel arrangement of β‐sheet structures. E) ThT fluorescence shows a significant enhancement of intensity for UICP14 as a function of concentration, indicating the high population of β‐sheet nanofibres. Enhancement of fluorescence was also observed for UICP16 but to a lower extent (n = 3, mean ± SD). F) CD spectrum of UICP14 showing a strong negative band at 222 nm and a strong positive peak at 204 nm indicating the formation of β‐sheet structure. The negative band at 230 nm is assigned to the aromatic Phg residue. G) Oscillatory rheology characterization showing frequency sweep measurements at strain (γ) 0.2%, within linear viscoelastic region, for peptides UICP14 and 16 (close symbols: G′; open symbols: G″). H) Log–log plot of shear moduli (G′) versus molar concentrations obtained for peptides UICP14 and 16. G′ values at 6 rad s^−1^ obtained from frequency sweep at 0.2% strain were used (n = 3, mean ± SD). Error bars in (B), (E), and (H) in some cases are smaller than the data mark sizes.

Introduction of a cationic K residue at the C‐terminus of the anionic UICP11 sequence, formed UICP13 ((PhgE)_2_K) (**Figure** **6**A and Table 1). UICP13 adopted β‐sheet structure only at pH 4.5 (Figure S6C, Supporting Information), where with increasing molar concentration a gradual increase in β‐sheet abundance was observed, accompanied by a reduction in random coil population (Figure 6B and Figure S7, Supporting Information). Below 45 mm UICP13 formed clear solutions, where it was mainly unstructured (for 30 mM, it was 42% random coil and 5% β‐sheet) (Figure 6B,C, and Figure S7, Supporting Information). While at 45, 60, and 75 mm turbid colloidal dispersions were formed, with high β‐sheet abundance detected indicating the formation of suspended fibers in water (Figure 6B,C, and Figure S7, Supporting Information). Interestingly, a blue shift was observed for C═O stretching peak of UICP13 compared to other UICPs, which appeared at 1634 cm^−1^ in the amide I band, suggesting an overall change in β‐sheet structure formation pattern (Figure 6D).

**Figure 7:**
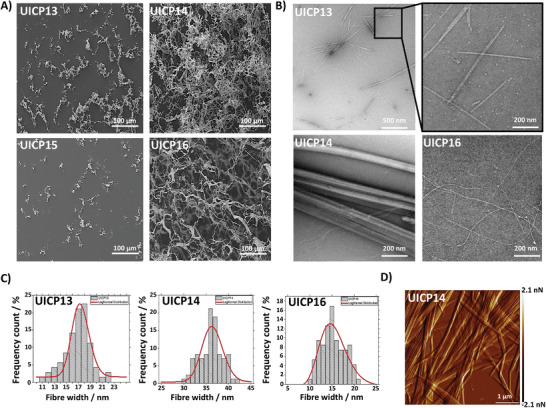
A) SEM micrographs of 45 mm UICP13‐16 peptide samples (all 20× diluted) showing continuous network of entangled nanofibers for UICP14 and 16, while both UICP13 and 15 showed amorphous peptide aggregates as it failed to self‐assemble into extended nanofibrous structures (Scale bar = 100 µm). B) TEM micrographs of 45 mm UICP13, 14, and 16 samples (10× diluted), showing the formation of extended peptide nanofiber bundles for UICP14 and 16, while very thin and short pseudocrystalline nanosheets were observed for UICP13 (Scale bar = 200 nm, also 500 nm is shown for UICP13). C) Histograms for size distribution for nanofibers of UICP13, 14, and 16 peptides measured from TEM micrographs, with average sizes of 17.4 ± 2.2, 36.6 ± 2.5, and 15.8 ± 2.6 nm (n = 100 ± SD), respectively. D) AFM Peak Force Error micrograph of a 50‐fold diluted 45 mm UICP14 hydrogel showing striations along the nanofibre bundle axis (Scale bar = 1 µm).

Despite β‐sheet fiber formations at concentrations ≥45 mm, no self‐supportive hydrogel was developed for the tested concentrations (Figure 6C). This is attributed to the presence of the extended K_5_ sidechain at the hydrophobic side that could hinder facile π–π interaction between core Phg residues, in addition to the electrostatic repulsion between core glutamates (both E_2_ and E_4_) at the hydrophilic side (Figure 6A). Collectively, these interactions have eventually led to the formation of very thin pseudocrystalline nanosheets (average thickness 17.4 ± 2.2 nm) (Figure 7B,C), which are unlike extended nanofibers, incapable of entanglement into continuous networks to form hydrogel within the studied concentration range, as revealed from TEM and SEM micrographs (**Figures** **7**A,B), respectively.

On the contrary, introducing K residue at the N‐terminus, i.e., K(PhgE)_2_ or UICP14 (Figure 6A and Table 1) demonstrated full recovery of propensity for self‐assembly, where FTIR confirmed the formation of β‐sheet structures from the presence of C═O stretching peaks (1624 cm^−1^) with anti‐parallel arrangement (1695 cm^−1^) in the amide I band, as for the C─N stretching and N─H bending peaks observed in amide II (1530 cm^−1^) and amide III (1250 cm^−1^) bands (Figure 6D, Characterization Table 1, and Characterization Figure C54, Supporting Information). The highest β‐sheet content 69% was achieved at pH 4.5 (Figure 6D and Figure S6C, Supporting Information), where the peptide possessed neutral net charge (Figure S6B, Supporting Information). A significant increase in the formation of nanofibres was also observed with increasing peptide concentration (ThT fluorescence; Figure 6E). CD measurement also confirmed the formation of anti‐parallel β‐sheet structure, where a strong negative band was observed at 222 nm with a strong positive peak detected at 204 nm. There is also an additional negative band at 230 nm, which is assigned to the aromatic Phg residue (Figure 6F).^[^ ^38^, ^79^ ^]^

Successful formation of hydrogels was achieved at pH range 1.3–8.5 (Figure S6A, Supporting Information), with low CGC ≤ 7.5 mm at pH 4.5 (Figure 6C) and high storage modulus G′ which exceeded 10 kPa at concentrations ≥45 mm (Figure 6G,H). The higher storage modulus arises from the formation of very thick extended cylindrical nanofibre bundles of diameter 36.6 ± 2.5 nm, as revealed from TEM (Figure 7B,C), which entangle into a dense continuous nanofibrous network forming self‐supportive opaque hydrogels (SEM, Figure 7A). AFM microscopy was performed to further confirm the morphology of the cylindrical bundles, which appeared to resemble “nanotubes” from TEM images (Figure 7C). AFM images clearly revealed longitudinal striations along the cylindrical structure axis, confirming the formation of thick cylindrical bundles through lateral association of thinner nanofibres, but not the formation of nanotubes (Figure 7D). Indeed, the presence of N‐terminus K residue at the hydrophilic side of the sequence favored lateral growth via the K_1_‐E_5_ countercharge electrostatic attraction (Figure 6A), hence the development of thick bundles (Figure 7B). Additionally, the unhindered π–π stacking of core Phg residues stabilized self‐assembly alongside the interchain H‐bonding between backbone amides (Figure 6A), both leading to the progressive growth of the β‐sheet ladder along the fiber axis, thus the formation of extended bundles (Figure 7B).

Likewise, both cationic counterparts, UICP15 ((PhgK)_2_E) and UICP16 (E(PhgK)_2_), showed similar trends (Figure 6A and Table 1). The presence of N‐terminal E_1_ residue in UICP16 recovered the tendency to self‐assembly through the ionic complementarity with C‐terminal K_5_, thus stabilizing lateral growth, as confirmed from FTIR data analysis (Figures 6A and 6D, and Figure S6C, Supporting Information). This led to the formation of viscoelastic self‐supportive hydrogels at pH range 2–8.5 (Figure S6A, Supporting Information), with CGC ≤ 30 mm at pH 4.5 (Figure 6C) due to the formation of very thin entangled nanofibres of diameter 15.8 ± 2.6 nm, as revealed by TEM (Figure 7B,C). According to SAXS data analysis, UICP16 at 30 mm and pH 4.5 also followed q^−1^ behavior with some aggregation noted at very low q values, similarly to UICP8 (Figure S3C, Supporting Information). By fitting the straight rod model, *R*_σ _= 1.2 ± 0.2 nm was obtained leading to fibers with the smallest diameter of 3.3 ± 0.2 nm (Table 1) across all tested UICPs. Again, this correlated very well with the obtained TEM (Figure 7B). Finally, pH shift to pH 11, also resulted in the shift of the curves to follow q^−4^ behavior at low q, indicating aggregated globules with a nearly smooth surface (Figure S3C, Supporting Information).^[^ ^66^, ^73^ ^]^ UICP16 structures are obviously thinner than those observed for the anionic counterpart UICP14 (Figure 7B,C), as electrostatic repulsion between the core protonated K_3_ residues should be expected at pH 4.5 (net charge +1, Figure S6B, Supporting Information), interfering with lateral growth, which happens to lesser extent between the shorter sidechains of the anionic core E_3_ residues in UICP14 (Figure 6A).

These structural features were reflected in the material bulk properties, where UICP16 formed transparent hydrogels of much lower storage modulus at concentrations ≤30 mm as compared to the stiffer UICP14 opaque hydrogels (Figure 6C,G,H). While in the case of UICP15, the presence of two protonated core K_2_ and K_4_ residues led to even more repulsion (Table 1), which therefore neither self‐assembled into β‐sheet nanofibres (Figures 6D and 7A, and Figure S6C, Supporting Information) nor formed hydrogels (data not shown). With regards to network properties, a power law of *C* ^1.7±0.4^ was obtained for UICP14 (Figure 6H), which is typical to extend rigid rod‐like structures contributing to network formation (Figure 6B).^[^ ^67^ ^]^ UIP16, on the other hand, had a power law with a significantly high exponent value *C* ^7±0.2^ (Figure 6H), which is a result of a network formed from highly polydisperse thin fibers, where some fibers are not contributing to the network (Figure 7B,C). Hence the actual effective concentration of fibers participating in the network elasticity is reduced compared to the nominal concentration leading to this high exponent value.^[^ ^38^, ^75^ ^]^ These results imply the very distinct nature of network elasticity for UICP14 and UICP16 (Figure S2C, Supporting Information).

### 2.4 The Design Rules

Collectively, the above results provided supramolecular, mechanical, and nano morphological characterization data to support stipulating structural design rules for UICPs. These rules were focused on electrostatic interactions and stemmed from the specific amino acids used here in this study (i.e., Phg, E, K), and were based on the patterns observed from the experimental data.

In light of this, we have shown that electrostatic attraction between terminal countercharge residues has obviously stabilized self‐assembly and triggered propagation of lateral growth through the hydrophilic side of the β‐sheet ladders (UICP3, UICP8, UICP14, and UICP16) (Tables 1 and 2). In principle, the presence of core E_3_ residue could slightly destabilize lateral growth via electrostatic repulsion, though this effect was not significant for both UICP3 and UICP14 due to the short sidechain of glutamates. However, this effect was not crucial for both peptides probably because of the conformation assumed by peptide dimers, whereby relatively elongated K_5_ residues are able to make strong interactions with both E_3_ and E_5_ while exposing Phg on the opposite side of the peptide. Furthermore, protonation of the E_3_ sidechain, not excluded at pH values of ≈4.5, was shown to significantly increase stabilization and allow for a different arrangement, similar but in principle more stable than that of UICP2. Similar reasoning should be held for UICP14. As a result, lateral growth into very thick fiber bundles was predominant in both peptides with the formation of hydrogels with larger storage moduli at relatively low CGC, thanks to the electrostatic attraction between terminal countercharge residues (Tables 1 and 2).

**Table 2:** Scoring system showing the level of contribution of different residues to stabilization of self‐assembly into β‐sheet ladder and propagation of lateral growth via electrostatic interactions.

Peptide	Sequence Pattern									Total Scores^a)^				β‐sheet fibre formation (Y/N)^b)^	Hydrogelation (Y/N)^b)^
	N‐terminus			Core			C‐terminus								
	Phg	Glu	Lys	Phg	Glu	Lys	Phg	Glu	Lys	Gr	O	Light	D		
UICP1	1			1	1				1	4	0	0	0	Y	Y
UICP2	1			1	1	1		1		3	2	0	0	Y	Y
UICP3		1		2	1				1	4	0	1	0	Y	Y
UICP4	1			1	1	1			1	0	1	2	2	N	N
UICP5			1	2	1				1	2	0	1	2	Y	N
UICP6	1			1		1		1		4	0	0	0	Y	Y
UICP7	1			1	1	1			1	2	1	2	0	N	N
UICP8			1	2		1		1		4	0	0	1	Y	Y
UICP9	1			1	1	1		1		3	2	0	0	Y	Y
UICP10		1		2		1		1		5	0	0	0	Y	Y
UICP11	1	1		1	1					0	2	0	2	N	N
UICP12	1		1	1		1				0	2	0	2	N	N
UICP13	1			1	2				1	0	3	2	0	Y	N
UICP14			1	2	1			1		4	0	1	0	Y	Y
UICP15	1			1		2		1		1	2	0	2	N	N
UICP16		1		2		1			1	4	0	0	1	Y	Y

^a)^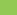 Stabilise; 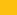 Slightly stabilise; 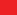 Slightly destabilise; 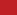 Destabilise;

^b)^ Y: Yes and N: No.

On the other hand, the electrostatic repulsion between the core K_3_ residues of longer sidechains significantly destabilized lateral growth leading to the formation of much thinner nanofibers in the case of UICP8 and UICP16 (Tables 1 and 2). Consequently, the resulting hydrogels from both peptides were generally softer than those formed by UICP3 and UICP14 and at higher CGC (30 mm). Strikingly, electrostatic repulsion between pairs of terminal K residues (K_1_‐K_5_) combined with possible repulsion between core E_3_ in UICP5 totally prevented the propagation of lateral growth, resulting in very thin entangled fibrils which formed viscous “gel‐like” materials at very high concentrations (≥75 mm), albeit lacking viscoelasticity (Tables 1 and 2). Similarly, strong electrostatic repulsion between the two pairs of core K residues (K_2_‐K_4_) in UICP15 both destabilized self‐assembly and interfered with lateral growth (Tables 1 and 2). A similar pattern of lateral growth stabilization through core‐to‐core electrostatic attraction between countercharge residue pairs in UICP2 (E_2_‐K_4_) and UICP9 (K_2_‐E_4_) resulted in similar morphology of nanofibres, both forming extended straight cylindrical structures (Tables 1 and 2).

Interestingly, core‐to‐terminal countercharge attraction in UICP10 (E_1_‐K_3_), on the other hand, led to the formation of a unique twisted braid‐like morphology with high stabilization of lateral growth and self‐assembly, which was evident from the very low CGC (≤7.5 mm) (Tables 1 and 2). The presence of a C‐terminal charged residue at the hydrophobic side of the peptide sequence was shown to influence hydrophobic packing, hence stability of self‐assembly. For instance, C‐terminal E residue slightly stabilized self‐assembly via the planar anion‐π attraction with the N‐terminal Phg residue or the formation of H‐bonds with the amino group of the first residue on the opposite strand (UICP2 and UICP9), though did not overcome the strong destabilization effect of repulsion between the two pairs of core K residues in UICP15 (Tables 1 and 2). While with a C‐terminal K residue, stabilization through cation‐π attraction with the N‐terminal Phg residue would be expected. This was not the case though, because of the bulky K sidechain which slightly destabilize self‐assembly via interfering with hydrophobic packing of β‐sheet ladders, leading either to failure in self‐assembly (UICP4 and UICP7) or impaired assembly forming non‐extended very short and thin fibres (UICP13), with no hydrogelation in both cases (Tables 1 and 2). In essence, the absence of charged residues on the hydrophobic side allowed for efficient π‐stacking between the two pairs of Phg residues, stabilizing self‐assembly (UICP3, UICP8, UICP10, UICP14, and UICP16) (Tables 1 and 2). Our results showed that the overall net charge (Z) of UICP chains was not a determinant factor for self‐assembly and lateral growth of nanofibres when comparing sequences with different charged residue distribution while having the same net charge status (e.g., UICP3 versus UICP13 (Z = ‐1e^−^); UICP8 versus UICP7 and 15 (Z = +1e^−^)). We also demonstrated how the differences in the formed nanostructures translate to the variability in elastic properties of network topologies, including resulting from preventing fiber−fiber association/aggregation showing to have a significant effect on the viscoelastic behaviour.

Based on the above, we have clear design rules for the effect of charge distribution on the molecular self‐assembly of short ionic self‐complementary sequences and the propagation of lateral growth for the formed β‐sheet fibers. These can be used to design similar and possibly longer ionic self‐complementary peptides with predictable molecular, nanoscopic, and mechanical properties.

## 3 Conclusion

In this work, we investigated and unravelled the effect of charge interaction on stability of self‐assembly into extended β‐sheet ladders and propagation of lateral growth via the hydrophilic sides of these assemblies. Ionic self‐complementarity of charged residues is a crucial design aspect to ensure self‐assembly for short ionic peptides. We have also demonstrated that distribution of charged residues is of a paramount importance for both self‐assembly and lateral growth to happen, consequently affecting fibre formation and morphology, as well as bulk material properties (hydrogelation and viscoelastic properties).

To this end, this study provided a better understanding of the role played by electrostatic interactions in governing the self‐assembly of ultra‐short ionic complementary peptides and nanostructure formation at the atomistic level. This understanding is crucial for the rational design of peptide nanomaterials and fine‐tuning its properties over the length scale to meet the application needs. The impact of this work could also extend to providing the bases for better understanding of the effect of charge interactions in protein aggregation and misfolding, where charge modifications, were linked to pathogenesis.^[^ ^80^ ^]^ Finally, we believe that data from empirical mechanistic studies should be twined with AI and machine learning to further support accurate prediction of peptide self‐assembly structures and aggregation propensity of protein molecules.^[^ ^81^ ^]^

## 4 Experimental Section

### Materials

All peptides were purchased from Biomatik Corporation (Wilmington, DE, Canada). Chemical identity and purity (>95%) of all peptides were confirmed by electrospray ionization mass spectroscopy (ESI‐MS), reverse‐phase high‐performance liquid chromatography (RP‐HPLC), ATR‐FTIR (4000–400 cm^−1^), and ^1^H nuclear magnetic resonance (^1^H NMR) (peptides characterisation data are provided in a separate supplementary document). Thioflavin T was purchased from Alfa Aesar (Heysham, UK), while all other reagents and solvents were purchased from Sigma‐Aldrich (Gillingham, UK) and used as received.

### Preparation of Hydrogels

Peptide powder was dissolved in HPLC water by mixing at 2500 rpm for 1 min using vortex mixer. The pH of peptide solution was then adjusted to 4.5, to trigger self‐assembly and gelation, by the stepwise addition of 0.5 m NaOH solution, while vortex mixing at 2500 rpm for 30–60 s in between additions. Final volume was adjusted using HPLC water to obtain the required peptide concentration, followed by storing the prepared hydrogels in fridge at 4 °C for 24 h to equilibrate, which were evaluated in the following day. Inversion of vials were conducted to determine critical gelation concentration (CGC), which was defined as the minimal peptide molar concentration at which the sample did not flow upon vial inversion.

### pH Titration for Phase Diagrams and Net Charge Calculation

pH titrations of peptide solution to create phase diagrams were conducted by preparing peptide solutions at a range of different molar concentrations (7.5–75 mm), which were then titrated by the stepwise addition of 0.5 m NaOH solution as described above. Both, pH values and physical appearance of the sample, were recorded after each addition for a wide pH range of ≈1.5–10. Equation (3) was used to calculate the theoretical net charge of different peptide sequences at each pH value:^[^ ^82^ ^]^

(3) $$Z=\sum_{i}N_{i}\frac{10^{pKa_{i}}}{10^{pH}+10^{pKa_{i}}}-\sum_{j}N_{j}\frac{10^{pH}}{10^{pH}+10^{pKa_{j}}}$$

where N_i/j_ are the numbers and pKa_i/j_ are the pKa values of the basic (i – p*K*a > 7) and acidic (j – p*K*a < 7) groups present on the peptide.

### Attenuated Total Reflectance–Fourier Transform Infrared Spectroscopy (ATR‐FTIR)

Evaluation of peptide secondary structure formation was carried out using Bruker Alpha Fourier transform infrared (FTIR) spectrometer equipped with a diamond multibounce attenuated total reflectance (ATR) plate. Peptide sample was applied onto the crystal and transmittance was recorded between 4000 and 400 cm^−1^ at 128 scans, with a resolution of 2 cm^−1^. HPLC water was used as background, which was automatically subtracted from the sample spectrum by OPUS version 8.1 software of the instrument. The amide I bands (1600–1700 cm^−1^) deconvolution was done using OriginPro 2016 software for peak separation and accurate calculation of peak areas. β‐sheet abundance was evaluated in each sample by calculating the ratio of the deconvoluted peak area for the prominent peak present at wavenumber 1611 cm^−1^ to 1630 cm^−1^, relative to the total area of the amide I region. All FTIR measurements were repeated in triplicates, using three different batches per sample.

### Circular Dichroism Spectroscopy (CD)

Samples were prepared by diluting hydrogels (10‐fold) in HPLC water, which were placed in 0.1 mm quartz cuvette (Hellma). Samples were then scanned using a ChiraScan, Applied Photophysics, where spectra were recorded at wavelength range 185–260 nm with 1 nm step size and 0.5 s response time. The ellipticity data were acquired in mdeg and corrected to molar ellipticity *ε* (deg cm^2^ dmol^−1^ residue^−1^) using the following formula:

(4) $$\varepsilon =\frac{\theta }{10CNL}$$

where *θ* is the ellipticity in mdeg, *C* is the sample molar concentration, *N* is the number of backbone amide bonds, and *L* is the cuvette optical path length in cm.

### Thioflavin T (ThT) Fluorescence Assay

ThT fluorescence assay was performed using SpectraMax M5 spectrofluorometer plate reader for the detection of amyloid‐like β‐sheet fibrils formation. ThT was dissolved in peptide solution with a final concentration of 100 µm, then the sample was titrated to pH 4.5 using 0.5 m NaOH as described above. Excitation wavelength used was λ_ex_ 440 nm and the fluorescence emission scan range was set to 460–600 nm. All fluorescence measurements were repeated in triplicates, using three different batches per sample.

### Oscillatory Rheology

The dynamic rheological properties of the developed materials were investigated using a stress‐controlled Anton Paar MCR‐501 Rheometer with a temperature‐controlled Peltier plate and a 25 mm parallel plate geometry equipped with a solvent trap to reduce evaporation. A 500 µL sample was loaded onto the stage with the gap set to 250 µm from the upper plate. Any excess material was removed with a spatula and the material was allowed to equilibrate at 37 °C for 2 min. The linear viscoelastic range was determined by conducting an amplitude sweep experiment (strains of 0.01−10%, 1 Hz and 37 °C), which was used to fix the strain at 0.2% for the following measurements. In order to examine the mechanical stiffness of the samples, radial frequency sweeps were carried out between 0.1 and 100 rad s^−1^ at 0.2% strain, with all measurements repeated in triplicates, using three different batches per sample.

### Scanning Electron Microscopy (SEM)

A Carl Zeiss EVO LS 15 SEM was used to characterize the nanofibrous network morphology. The peptide hydrogel was diluted 20‐fold using HPLC water followed by mounting onto an aluminium stub with a carbon tab, which was freeze dried overnight and gold sputter‐coated (10 nm) in a Quorum QR150S coater before imaging. Samples were analyzed with an accelerating voltage of 10 kV and a probe current of 100 pA.

### Transmission Electron Microscopy (TEM)

A JEOL JEM‐1400 TEM equipped with an Emsis Xarosa digital camera with Radius software was used to characterise nanofibre morphology at accelerating voltage of 120 keV. Grids were glow discharged in a Quorum Gluqube plus at 20 mA for 30 seconds, to prevent sample aggregation. Hydrogel was tenfold diluted using HPLC water and a 10 µL sample was applied onto the carbon‐coated face of the copper grid (400 mesh) for 10 s. The sample was blotted away using filter papers, washed with 10 µL of pure water and finally negatively stained with 10 µL of 2% (w/v) uranyl acetate solution. Nanofiber diameters were calculated using ImageJ 1.54d software, where the thickness of each nanofibre was measured at three different locations along the fibre length in all TEM micrographs obtained from imaging three different batches per sample (total ≈80–100 diameter measurement per sample). Histograms for size distribution were fitted using OriginPro 2016 software to calculate the average diameter.

### Atomic Force Microscopy (AFM)

UICP14 sample was 50‐fold diluted from a 45 mm hydrogel in ddH2O. A total of 25 µL was dropped onto a freshly cleaved mica surface, where excess solution was removed after 2 min followed by washing once with 1 mL of HPLC grade H_2_O. Excess water was removed by wicking using Whatman No. 1 filter paper and the sample was air‐dried overnight prior to imaging. AFM imaging was performed in PeakForce Tapping (PFT) mode in air using a Bruker Resolve BioAFM equipped with a Nanoscope V controller operating under Nanoscope v9.7 software. Imaging was performed using ScanAsyst Air tips. These silicon nitride probes with Al coating have a nominal radius of curvature of ≈2–5 nm and a nominal spring constant of 0.4 N m^−1^ (Bruker AFM Probes, Camarillo, CA, USA). Height and PFT images with scan sizes of 5 µm were captured at a scan rate of 1.98 Hz. The instrument is periodically calibrated using a grating with 180 nm deep and 10 mm^2^ depressions. Data was second‐order flattened using the Nanoscope Analysis (v3.0) software prior to image export.

### Small Angle X‐ray Scattering (SAXS)

SAXS experiments were performed on beamline B21 and I22 at the Diamond Light Source (DLS) facility in Harwell Science and Innovation Campus, Didcot, UK.

For I22 the following setup was used. The energy of the beam was 12.4 keV corresponding to the X‐ray wavelength of 1 Å. Quartz capillaries (1.5 mm outer diameter, 0.01 mm wall thickness) were supplied from the Capillary Tube Supplies Ltd., UK. All samples were introduced to capillaries via syringe. Samples were collected at 21 ± 1 °C for 99 consecutive frames at the exposure time of 1 s each to check for radiation damage. Calibration of the SAXS detector (Pilatus P3−2M, Dectris, Switzerland) was performed using silver behenate powder. The distance between samples and the detector was fixed to 4.2 m to cover a momentum transfer vector range of to an accessible momentum transfer vector range of 0.049 nm^−1^ < *q*  =  (4π/λ) sin(θ/2) < 4.7 nm^−1^, where θ is the scattering angle and λ is the wavelength of incident photons.

Measurements performed on beamline B21 followed the setup previously described.^[^ ^83^, ^84^, ^85^ ^]^ The energy of the beam was 13.05 keV, corresponding to an X‐ray wavelength of 0.95 Å. Arinax sample change robot was used to automatically load the samples into Quartz capillaries (1.5 mm outer diameter, 0.01 mm wall thickness, Capillary Tube Supplies Ltd. UK). The sample‐to‐detector distance was fixed to 3.7 m corresponding to an accessible momentum transfer vector range of 0.03 nm^−1^ < *q*  =  (4π/λ) sin(*θ*/2) < 3.4 nm^−1^, where *θ* is the scattering angle and λ the wavelength of the incident photons. Calibration of the SAXS detector (Eiger 4M, Dectris, Switzerland) was performed using silver behenate powder. Samples were collected at 20 ± 1 °C for 20 consecutive frames at the exposure time of 1 s each to check for radiation damage.

Empty capillaries were used as background and subtracted from all measurements, while the subtraction mask was created using glassy carbon. Data were reduced using the processing tools at DawnDiamond software suite. The 2D scattering photon patterns were integrated using azimuthal integration tool to obtain a 1D scattering patterns.

### Molecular Dynamics (MD) Simulations

For each pentapeptide UICP2, UICP3, UICP4, UICP5, and UICP10, a µs‐long MD simulation of the self‐assembly process of 432 molecules in KCl water solution (as many ions as to reach electrical neutrality were added to the solution) using the AMBER20 simulation package^[^ ^86^ ^]^ as in previous work.^[^ ^34^, ^43^, ^87^ ^]^

Parameters of N‐terminal and standard Phg residues were estimated following the guidelines reported on the AMBER website (http://ambermd.org/tutorials/basic/tutorial5/) using the ff19SB AMBER force field,^[^ ^88^ ^]^ together with an OPC model for water.^[^ ^89^ ^]^ Next, models of elongated zwitterionic pentapeptides were generated using the *sequence* command of AmberTools20 package. The initial conformation of the assembling peptides was generated by placing their centers of mass on a 6×6×12 grid of points spaced 20, 18, and 12 Å along the *x*, *y*, and *z* directions, respectively. The peptides were oriented parallel to each other with the N‐to‐C axis lying on the *x* coordinate and centered in a rectangular box so that the minimum distance of the molecule from any face was larger than 6 Å.

All the simulations were performed as follows. First, three consecutive restrained structural optimizations (up to 25 000 steps) were performed in the presence of harmonic restraints (k = 1 kcal mol^−1^ Å^−1^) applied to: a) all non‐hydrogenous atoms of the system; b) backbone atoms; c) C_α_ atoms. Reference structures at steps b) and c) were the final ones from the previous step. Next, up to 50 000 cycles of unrestrained optimization were performed. Each system was then heated to 310 K in 10 ns via constant‐pressure‐temperature (NTP) MD simulations, followed by an equilibration phase of 100 ns. Starting from the equilibrated structure, production MD simulations were performed for each system. Pressure and temperature were set to 1 atm and 298 K (after the equilibration phase) using the isotropic Berendsen barostat and the Langevin thermostat, respectively. A time step of 2 fs was used for all the simulation steps. Periodic boundary conditions were employed, and electrostatic interactions were estimated using the Particle Mesh Ewald scheme with a cutoff of 9.0 Å for the short‐range evaluation in direct space and for Lennard‐Jones interactions (with a continuum model correction for energy and pressure).

The analysis of the simulations was performed using the *cpptraj* suite of the AMBER20 package, the *saltbr* function of VMD1.9.4, and *in house* scripts. In particular, peptide pairs were detected by imposing the concomitant occurrence of salt bridges between selected amino acids (Supplementary Table S1).

### Statistical Reproducibility and Data Analysis

To ensure reproducibility of data, material characterization experiments were performed using at least three different batches of hydrogel preparations per peptide and per experimental condition tested. Deconvolution of ATR‐FTIR amide I band peaks was performed using OriginProTM 2016 software, where spectra measurements of three different samples were used to calculate the mean ± standard deviation (n = 3, mean ± SD) of the relative secondary structure population. Likewise, frequency sweep measurements were also performed using sample size n = 3 to calculate the mean ± SD of storage modulus G′ values at 6 rad s^−1^. Mean ± SD were calculated using Microsoft Excel for Microsoft 365 MSO, version 2303. TEM measurements of nanofibre diameters were calculated as the average of nanofibre thickness at three different locations along the fiber length in all TEM micrographs obtained from imaging three different batches per sample (total ≈80–100 diameter measurement per sample). Nanofibers size distribution was estimated from histograms by LogNormal fitting to calculate the average diameter and size range using OriginProTM 2016 software.

## Conflict of Interest

The authors declare no conflict of interest.

## Author Contributions

Conceptualization, M.A.N.S., A.K., J.K.W., A.V.V., and M.A.E.; methodology, M.A.N.S., A.K., T.S., R.Ar., N.A., F.I.G., M.H.A., J.K.W., A.V.V., and M.A.E.; formal analysis, M.A.N.S., A.K., C.J.C.E.G., J.K.W., A.V.V., and M.A.E.; data curation, M.A.N.S., A.K., R. Ar., N.A., F.I.G., R.Ala., C.J.C.E.G., J.K.W., A.V.V., and M.A.E.; writing—original draft preparation, M.A.N.S., A.K., J.K.W., A.V.V., and M.A.E.; writing—review and editing, M.A.N.S., A.K., T.S., R.Ar., N.A., F.I.G., M.H.A., J.K.W., A.V.V., and M.A.E.; supervision, R.All., K.L. and M.A.E. All authors have read and agreed to the published version of the manuscript.

## Supporting information



Supporting Information

Supporting Information

## Data Availability

The data that support the findings of this study are available from the corresponding author upon reasonable request.
