# Supplementary material for: Unraveling the Atomistic Mechanism of Electrostatic Lateral Association of Peptide β‐Sheet Structures and Its Role in Nanofiber Growth and Hydrogelation
Source: Small. 2025 Jan 9;21(6):2408213. doi: 10.1002/smll.202408213 (PMC11817957; doi:10.1002/smll.202408213)
Supplement: Supplementary file 1 — Supporting Information [file SMLL-21-2408213-s002.docx]

**Supplementary Information**

**Unravelling the atomistic mechanism of electrostatic lateral association of peptide β-sheet structures and its role in nanofibre growth and hydrogelation**

Mohamed A. N. Soliman^1,2^, Abdulwahhab Khedr^1,3^, Tarsem Sahota^1^, Rachel Armitage^1,4^, Raymond Allan^1^, Katie Laird^1^, Natalie Allcock^5^, Fatmah I. Ghuloum^6^, Mahetab H. Amer^6^, Reem Alazragi^1,7^, Charlotte J.C. Edwards-Gayle^8^, Jacek K. Wychowaniec^9^, Attilio V. Vargiu^10^, Mohamed A. Elsawy^1,11*^

^1^ Leicester Institute for Pharmaceutical Innovation, Leicester School of Pharmacy, De Montfort University, The Gateway, Leicester LE1 9BH, United Kingdom

^2^ Department of Pharmaceutics and Industrial Pharmacy, Faculty of Pharmacy, Cairo University, Cairo 11562, Egypt

^3^ Department of Pharmaceutics and Industrial Pharmacy, Faculty of Pharmacy, Zagazig University, Zagazig, Egypt

^4^ School of Archaeology and Ancient History, University of Leicester, Leicester, LE1 7RH, United Kingdom

^5^ Electron Microscopy Facility Core Biotechnology Services, College of Life Sciences, University of Leicester, Leicester LE1 7RH, United Kingdom

^6^ Division of Cell Matrix and Regenerative Medicine, School of Biological Sciences, University of Manchester, Oxford Road, Manchester M13 9PL, United Kingdom

^7^ Department of Biological Science, College of Science, University of Jeddah, Jeddah 21493, Saudi Arabia

^8^ Diamond Light Source, Harwell Science and Innovation Campus, Fermi Avenue, Didcot, OX110DE, United Kingdom

^9^ AO Research Institute Davos, Clavadelerstrasse 8, Davos, 7270, Switzerland

^10^ Physics Department, University of Cagliari, s.p. 8, km. 0.700, 09042 Monserrato, Italy

^11^ Division of Pharmacy and Optometry, School of Health Sciences, University of Manchester, Oxford Road, Manchester M13 9PL, United Kingdom

* Address correspondence to: [mohamed.elsawy@manchester.ac.uk](mailto:mohamed.elsawy@manchester.ac.uk)

**Table S1.** Salt bridges that could possibly be formed between two strands of selected peptides.

| **Peptide** | **Salt bridges (strand1-strand2)** |
| --- | --- |
| UIPC2 | E_2_-K_4_ AND K_4_-E_2_ |
| UIPC3 | E_1_-K_5_ AND K_5_-E_1_ |
| UIPC4 | (E_2_-K_4_ OR E_2_-K_5_) AND (K_4_-E_2_ OR K_5_-E_2_) |
| UIPC5 | (E_3_-K_5_ AND K_5_-E_3_) OR (E_3_-K_1_ AND K_1_-E_3_) |
| UIPC10 | (E_1_-K_3_ AND K_3_-E_1_) OR (K_3_-E_5_ AND E_5_-K_3_) |

**Table S2.** Number of peptide dimers interacting for more than 1% of the simulation time. Numbers in parentheses in the second column indicate the longest lifetime detected for a single peptide dimer across the MD simulation. During the remaining time, peptides form occasional interactions either with other partners and/or with the solvent.

| **Peptide** | **# salt bridges detected** |
| --- | --- |
| UIPC2 | 25 (6%) |
| UIPC3 | 5 (2%) |
| UIPC3 (neutral E_3_) | 10 (3%) |
| UIPC4 | 27 (10%) |
| UIPC5 | 50 (8%) |
| UIPC10 | 30 (22%) |

**
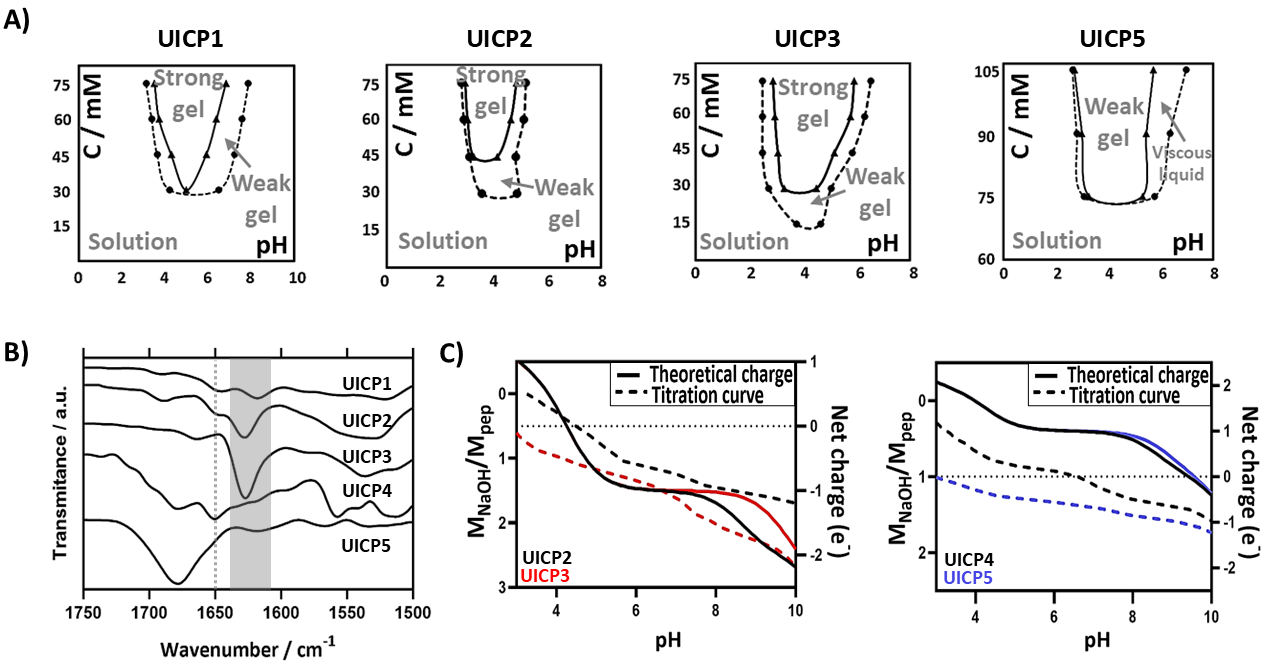
**

**Figure S1. A)** Phase diagrams of UICP1, 2, 3 and 5 as a function of both pH and concentration. Three different phases were observed for UICP1, 2 and 3: strong gel, weak gel, and solution, while week gel, viscous liquid and solution were observed for UICP5. **B)** ATR-FTIR spectra for UICP1-5 at 75 mM showing β-sheet peaks for all peptides (1611 cm^−1^ to 1630 cm^−1^, shaded in grey) except for UICP4, which failed to self-assemble into β-sheet structure and showed only a random coil peak (1650 cm^−1^, dotted line). **C)** Theoretical net charge state of UICP2, 3, 4 and 5 as a function of pH and molar ratio of added NaOH solution to the UICP peptide solution (M_NaOH_/M_pep_). Theoretical net charge was calculated using equation 3 (reported in methods). The dashed line indicates zero net charge.

**
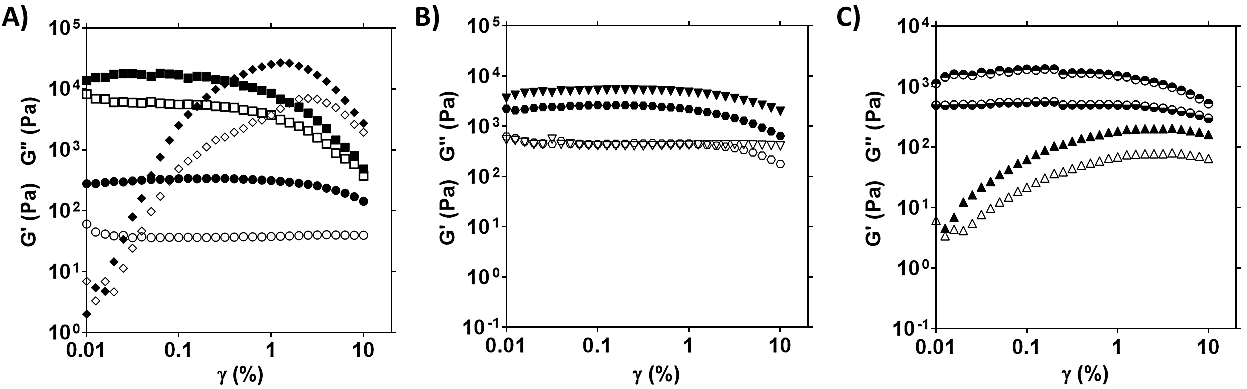
**

**Figure S2.** Oscillatory rheology characterisation of elasticity showing strain (ɣ) sweep for peptides **(A)** UICP1 (●, ○), UICP2 (■, □), UICP5 (⧫, ◊), **(B)** UICP9 (▼, ▽), UICP10 (
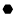
,
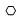
), **(C)** UICP14 (
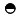
,
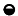
), and UICP16 (▲, Δ), (close symbols and
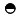
: G′; open symbols and
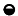
: G″). All UIPs were measured at 45 mM concentration with exception of UICP5, which was measured at 75 mM where it formed viscous solution.

**
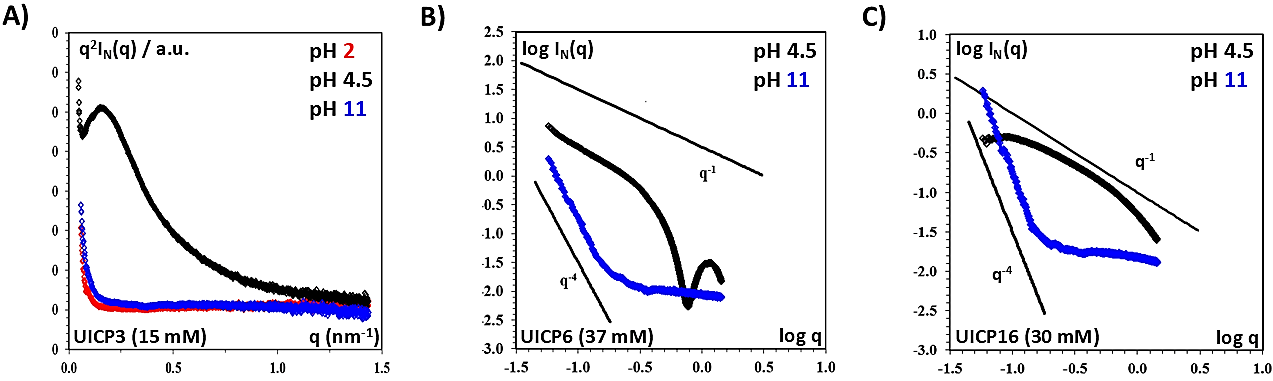
**

**Figure S3. (A)** Kratky plot (q^2^ I_N_(q) vs q representation) of UICP3 prepared at different pH values at 15 mM concentration. **(B)** SAXS characterization of UICP6 prepared at different pH values and at 37 mM concentration in double logarithmic plot of I_N_(q) vs q representation. The straight lines depict type of slope for easier visualisation. **C)** SAXS characterization of UICP16 prepared at different pH values in double logarithmic plot of I_N_(q) vs q representation. The straight lines depict type of slope for easier visualisation.

**
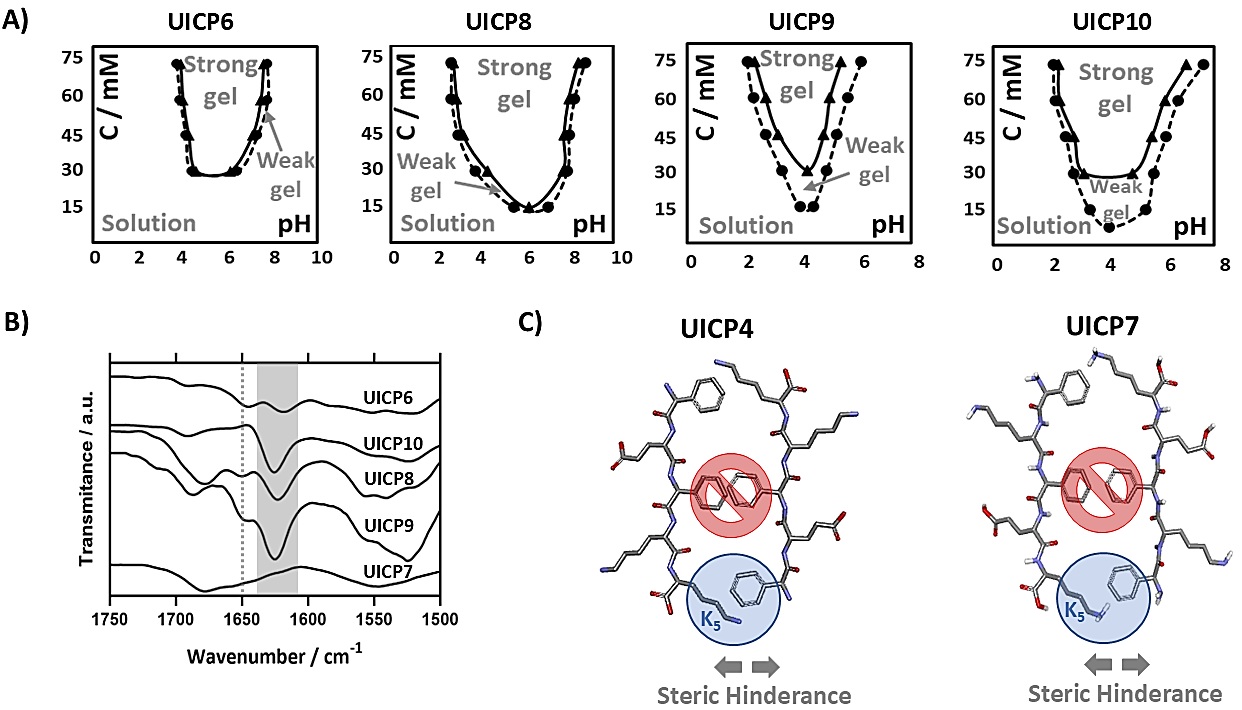
**

**Figure S4. A)** Phase diagrams of UICP6, 8, 9 and 10 as a function of both pH and concentration. Three different phases were observed: strong gel, weak gel, and solution. **B)** ATR-FTIR spectra for UICP6-10 at 75 mM showing β-sheet peaks for all peptides (1611 cm^−1^ to 1630 cm^−1^, shaded in grey) except for UICP7, which was unstructured. Apart from UICP10, all peptides showed a relatively very low random coil peak area (1650 cm^−1^, dotted line). **C)** Schematic representation of UICP4 and 7 showing the possible steric hinderance of the bulky K_5_ side chain at the hydrophobic face, which could interfere with occurrence of the aromatic interactions between core Phg residues at position 2.

**
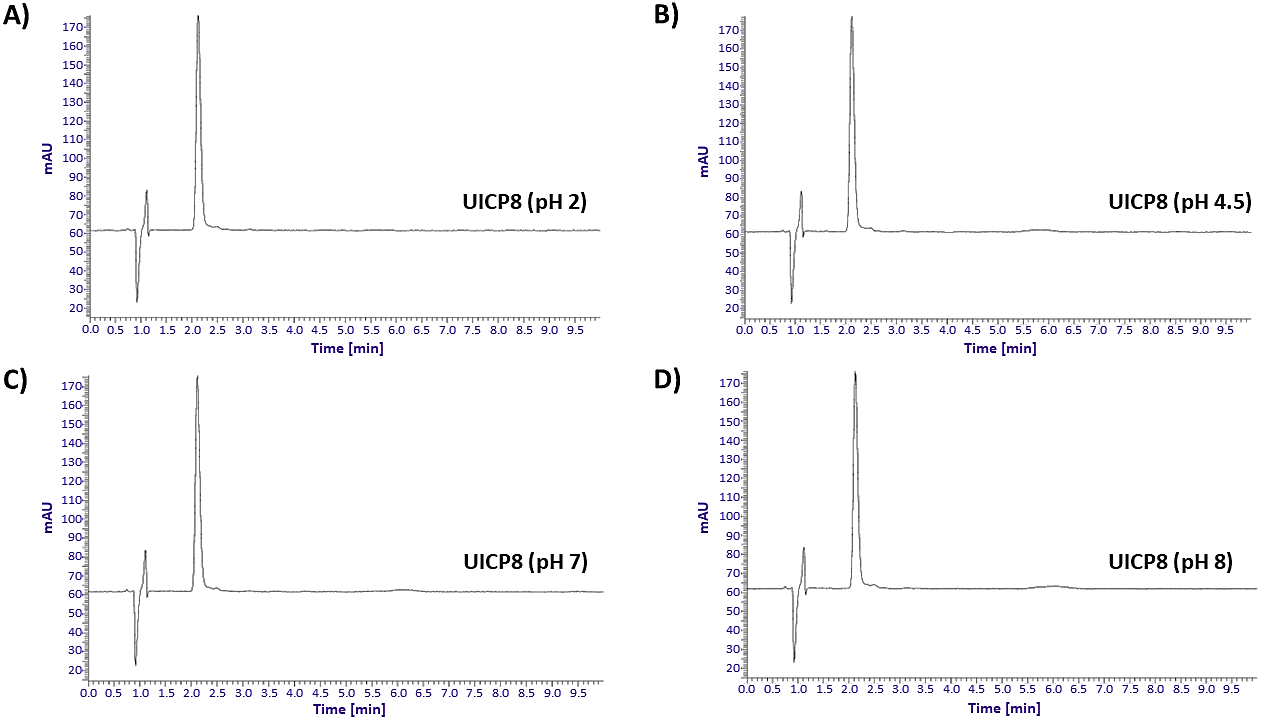
**

**Figure S5.** RP-HPLC traces for UICP8 at pH values **A)** 2, **B)** 4.5, **C)** 7 and **D)** 8, all showing a single sharp symmetrical peak at retention time 2.12 min., ruling out the presence of diastereoisomers that might result from the racemisation of *L*-Phg residues at positions P2 and P4. The peptide aqueous solutions (100 μg/mL) were injected on the RP-HPLC analytical scale Phenomenex Jupiter 4µ Proteo column 90A° (150 x 4.6 mm), at a flow rate of 2 mL/min. Isocratic separation was performed using 90% water (H_2_O) / 10% acetonitrile (CH_3_CN) (all solvents contained 0.05 % of trifluoroacetic acid) over 10 min, and peaks were detected by a UV detector at a wavelength λ 220 nm.

**
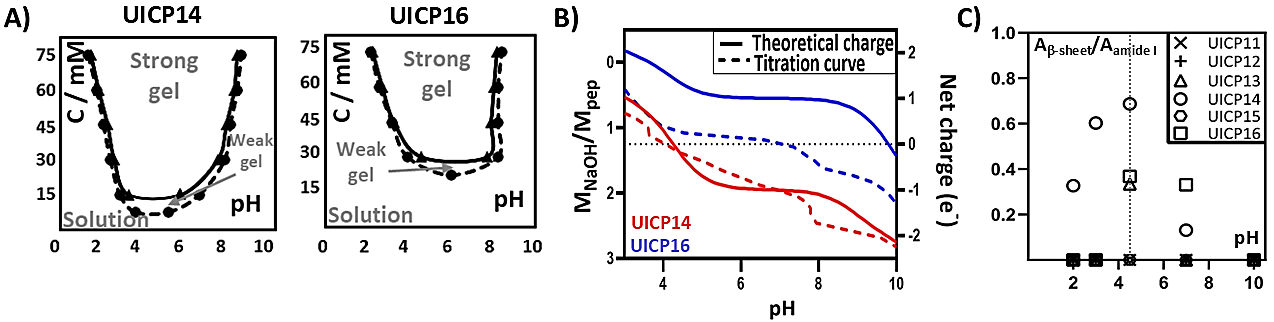
**

**Figure S6. A)** Phase diagrams of UICP14 and 16 as a function of both pH and concentration. Three different phases were observed: strong gel, weak gel, and solution. **B)** Theoretical net charge state of UICP14 and 16 as a function of pH and molar ratio of added NaOH solution to the UICP peptide solution (M_NaOH_/M_pep_). Theoretical net charge was calculated using equation 3. The dashed line indicates zero net charge. **C)** Relative β-sheet peak area for peptides UICP11-16 over a range of different pH values (n=3, mean ± SD). Highest relative area was observed for UICP13, 14 and 16 at pH 4.5, which is considered the optimal value for self-assembly, with the highest β-sheet content observed for UICP14. Error bars in some cases are smaller than the data mark sizes. Error bars are smaller than the data mark sizes.

**
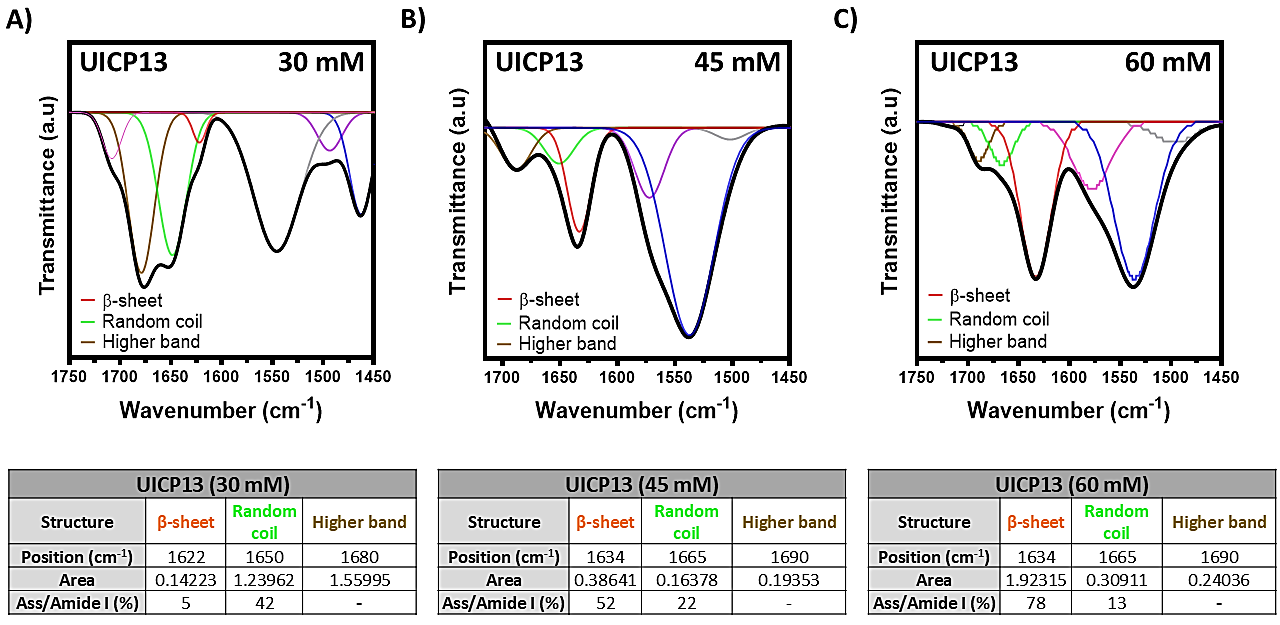
**

**Figure S7.** ATR-FTIR spectra peak deconvolution for amide I band of UICP13 at concentrations **A)** 30 mM, **B)** 45 mM and **C)** 60 mM. The amide I bands (1600–1700 cm^–1^) deconvolution was done using OriginPro^TM^ 2016 software for peak separation and accurate calculation of peak areas, as depicted in the summary Table below. Relative population of secondary structures (ss) have been calculated as the ratio of the deconvoluted ss peak area relative to the total amide I band area. The above figures show one example for amide I band peaks deconvolution per concentration. The relative ss population was calculated from the deconvolution of at least three spectra measurements for three different preparation per concentration to calculate the mean + SD, which were reported in Figure 6B in the main paper.
