# Supplementary material for: Unraveling the Atomistic Mechanism of Electrostatic Lateral Association of Peptide β‐Sheet Structures and Its Role in Nanofiber Growth and Hydrogelation
Source: Small. 2025 Jan 9;21(6):2408213. doi: 10.1002/smll.202408213 (PMC11817957; doi:10.1002/smll.202408213)
Supplement: Supplementary file 2 — Supporting Information [file SMLL-21-2408213-s001.docx]

**Peptides Characterisation Data**

**Unravelling the atomistic mechanism of electrostatic lateral association of peptide β-sheet structures and its role in nanofibre growth and hydrogelation**

Mohamed A. N. Soliman^1,2^, Abdulwahhab Khedr^1,3^, Tarsem Sahota^1^, Rachel Armitage^1,4^, Raymond Allan^1^, Katie Laird^1^, Natalie Allcock^5^, Fatmah I. Ghuloum^6^, Mahetab H. Amer^6^, Reem Alazragi^1,7^, Charlotte J.C. Edwards-Gayle^8^, Jacek K. Wychowaniec^9^, Attilio V. Vargiu^10^, Mohamed A. Elsawy^1,11*^

^1^ Leicester Institute for Pharmaceutical Innovation, Leicester School of Pharmacy, De Montfort University, The Gateway, Leicester LE1 9BH, United Kingdom

^2^ Department of Pharmaceutics and Industrial Pharmacy, Faculty of Pharmacy, Cairo University, Cairo 11562, Egypt

^3^ Department of Pharmaceutics and Industrial Pharmacy, Faculty of Pharmacy, Zagazig University, Zagazig, Egypt

^4^ School of Archaeology and Ancient History, University of Leicester, Leicester, LE1 7RH, United Kingdom

^5^ Electron Microscopy Facility Core Biotechnology Services, College of Life Sciences, University of Leicester, Leicester LE1 7RH, United Kingdom

^6^ Division of Cell Matrix and Regenerative Medicine, School of Biological Sciences, University of Manchester, Oxford Road, Manchester M13 9PL, United Kingdom

^7^ Department of Biological Science, College of Science, University of Jeddah, Jeddah 21493, Saudi Arabia

^8^ Diamond Light Source, Harwell Science and Innovation Campus, Fermi Avenue, Didcot, OX110DE, United Kingdom

^9^ AO Research Institute Davos, Clavadelerstrasse 8, Davos, 7270, Switzerland

^10^ Physics Department, University of Cagliari, s.p. 8, km. 0.700, 09042 Monserrato, Italy

^11^ Division of Pharmacy and Optometry, School of Health Sciences, University of Manchester, Oxford Road, Manchester M13 9PL, United Kingdom

* Address correspondence to: [mohamed.elsawy@manchester.ac.uk](mailto:mohamed.elsawy@manchester.ac.uk)

**Characterisation Table 1.** summary of the characterisation data for the chemical purity (RP-HPLC) and identity (ESI-MS, ^1^H NMR and ATR-FTIR) of the reported UICPs

| **Peptide** | **RP-HPLC*** | | **ESI-MS**** | | **^1^H NMR (D_2_O, 600 MHz)^¶^**  **δ ppm** | **ATR-FTIR^#^**  **(ν 4000-400 cm^-1^)** |
| --- | --- | --- | --- | --- | --- | --- |
|  | **Purity (%)** | **RT (min)** | **Calculated Mw** | **Found mass (m/z)** |  |  |
| **UICP1** | 96 | 3.78 | 541.6 | 542.25 [M+H]^+^  1083.30 [2M+H]^+^ | 1.28 (2H, t), 1.55 (2H, t), 1.62-1.98 (4H, q), 2.37 (2H, t), 2.85 (2H, t), 4.22 (1H, t), 4.41 (1H, t), 5.05 (1H), 5.25 (1H), 7.32-7.42 (10H, d & t) | 3440 cm^-1^, O-H & N-H stretching  3050 cm^-1^, C-H aromatic stretching  2946 cm^-1^, C-H aromatic stretching  1616 cm^-1^ & 1689 cm^-1^, C=O stretching (anti-parallel β-sheet, amide I)  1645 cm^-1^, C=O stretching (random coil, amide I)  1521 cm^-1^, C-N stretching & N-H bending  1247 cm^-1^, C-N stretching & N-H bending (β-sheet, amide III) |
| **UICP2** | 97 | 5.99 | 670.71 | 671.35 [M+H]^+^ | 1.28 (2H, t), 1.36 (2H, t), 1.52 (2H, t), 1.70-2.17 (6H, q), 2.33 (4H, t), 2.88 (2H, t), 4.17-4.34 (3H, t), 5.04 (1H), 5.22 (1H), 7.21-7.42 (10H, d & t) | 3350-3634 cm^-1^, O-H stretching  3280 cm^-1^, N-H stretching  3046 cm^-1^, C-H aromatic stretching  2935 cm^-1^, C-H aliphatic stretching  1628 cm^-1^ & 1689 cm^-1^, C=O stretching (anti-parallel β-sheet)  1649 cm^-1^, C=O stretching (random coil, amide I)  1530 cm^-1^, C-N stretching & -NH bending (β-sheet, amide II)  1248 cm^-1^, C-N stretching & N-H bending (β-sheet, amide III) |
| **UICP3** | 94 | 6.47 | 669.77 | 671.30 [M+H]^+^ | 1.30 (4H, t), 1.56 (4H, t), 1.76-2.10 (8H, q), 2.38 (4H, t), 2.52 (4H, t), 2.86 (1H, t), 4.02 (1H, t), 4.136-4.43 (3H, t), 5.24 (1H), 5.34 (1H), 7.17-7.36 (10H, d & t) | 3308 cm^-1^, O-H & N-H stretching  3046 cm^-1^, C-H aromatic stretching  2952 cm^-1^, C-H aliphatic stretching  1626 cm^-1^, C=O stretching (anti-parallel β-sheet, amide I)  1670 cm^-1^, C=O stretching (unordered/turn, amide I)  1535 cm^-1^, C-N stretching & N-H bending  1238 cm^-1^, C-N stretching & N-H bending (β-sheet, amide III) |
| **UICP4** | 96 | 2.02 | 669 | 670.35 [M+H]^+^ | 1.37 (4H, t), 1.52 (4H, t), 1.68-2.17 (6H, q), 2.43 (2H, t), 2.80 (4H, t), 4.21-4.45 (3H, t),5.07 (1H), 5.25 (1H), 7.23-7.43 (10H, d & t) | 3316-4000 cm^-1^, O-H & N-H stretching  3055 cm^-1^, C-H aromatic stretching  2976 cm^-1^, C-H aliphatic stretching  1650 cm^-1^, C=O stretching (random coil, amide I)  1676 cm^-1^, C=O stretching (unordered/turn, amide I)  1550 cm^-1^, C-N stretching & N-H bending  1258 cm^-1^, C-N stretching & N-H bending (unordered, amide III) |
| **UICP5** | 97 | 3.7 | 669.67 | 670.35 [M+H]^+^ | N/A | 3360-3628 cm^-1^, O-H stretching  3261 cm^-1^, N-H stretching  3034 cm^-1^, C-H aromatic stretching  2928 cm^-1^, C-H aliphatic stretching  1628 cm^-1^, C=O stretching (anti-parallel β-sheet, amide I)  1675 cm^-1^, C=O stretching (unordered/turn, amide I)  1554 cm^-1^, 1527 cm^-1^, C-N stretching & N-H bending  1266 cm^-1^, C-N stretching & N-H bending (unordered, amide III) |
| **UICP6** | 98 | 4.97 | 541.6 | 542.35 [M+H]^+^  1083.35 [2M+H]^+^ | 1.31 (2H, t), 1.57 (2H, t), 2.31-2.24 (4H, q), 2.86-2.88 (4H, t), 4.25-4.36 (2H, t), 5.04 (1H), 5.27 (1H), 7.20-7.43 (10H, d & t) | 3340-3634 cm^-1^, O-H stretching  3286 cm^-1^, N-H stretching  3055 cm^-1^, C-H aromatic stretching  2976 cm^-1^, C-H aliphatic stretching  1618 cm^-1^ & 1689 cm^-1^, C=O stretching (anti-parallel β-sheet, amide I)  1647 cm^-1^, C=O stretching (random coil, amide I)  1523 cm^-1^, C-N stretching & N-H bending  1257 cm^-1^, C-N stretching & N-H bending |
| **UICP7** | 95 | 2.37 | 669.76 | 670.35 [M+H]^+^ | 1.49 (4H, t), 1.58 (4H, t), 1.88-2.13 (4H, q), 2.34 (2H, q), 2.79 (2H, t), 2.88 (4H, t), 4.28 (1H, t), 4.36 (2H, t), 5.04 (1H), 5.25 (1H), 7.21-7.41 (10H, d & t) | 3668 cm^-1^, O-H & N-H stretching  3067 cm^-1^, C-H aromatic stretching  2989 cm^-1^, C-H aliphatic stretching  1677 cm^-1^, C=O stretching (unordered/turn, amide I)  1547 cm^-1^, C-N stretching & N-H bending  1254 cm^-1^, C-N stretching & N-H bending (unordered, amide III) |
| **UICP8** | 98 | 2.12 | 669.76 | 668.30 [M-H]^-^ | 1.56-1.65 (8H, t), 1.85-1.86 (6H, q), 2.231 (2H, t), 2.88 (4H, t), 3.94 (1H, t), 4.22 (2H, t), 5.25 (1H), 5.33 (1H), 7.17-7.35 (10H, d & t) | 3386-3637 cm^-1^, O-H stretching  3270 cm^-1^, N-H stretching  3043 cm^-1^, C-H aromatic stretching  2973 cm^-1^, C-H aliphatic stretching  1623 cm^-1^ & 1680 cm^-1^, C=O stretching (anti-parallel β-sheet, amide I)  1650 cm^-1^, C=O stretching (random coil, amide I)  1557cm^-1^, C-N stretching & N-H bending  1248 cm^-1^, CN stretching & NH bending |
| **UICP9** | 98 | 2.79 | 670.00 | 671.25 [M+H]^+^ | 1.36 (2H, t), 1.58 (2H, t), 1.70-1.72 (4H, q), 1.90 (4H, q), 2. 17 (2H, q), 2.33 (2H, t), 2.88 (2H, t), 4.29-4.34 (3H, t), 5.05 (1H), 5.22 (1H), 7.21-7.42 (10H, d & t) | 3350-4000 cm^-1^, O-H stretching  3286 cm^-1^, N-H stretching  3050 cm^-1^, C-H aromatic stretching  2934 cm^-1^, C-H aliphatic stretching  1624 cm^-1^ & 1689 cm^-1^, C=O stretching (anti-parallel β-sheet, amide I)  1650 cm^-1^, C=O stretching (random coil, amide I)  1523 cm^-1^, C-N stretching & N-H bending  1243 cm^-1^, C-N stretching & N-H bending (β-sheet, amide III) |
| **UICP10** | 96 | 8.28 | 670.71 | 671.35 [M+H]^+^  693.35 [M+Na]^+^  1341.65 [2M+H]^+^ | 1.37 (2H, t), 1.58 (2H, t), 1.96 (4H, q), 2.11 (2H, t), 2.23 (4H, t), 2.49 (2H, t), 2.87 (1H, t), 4.30 (2H, t), 5.27 (1H), 5.35 (1H), 7.18-7.37 (10H, d & t) | 3350-4000 cm^-1^, O-H stretching  3277 cm^-1^, N-H stretching  3050 cm^-1^, C-H aromatic stretching  2943 cm^-1^, C-H aliphatic stretching  1625 cm^-1^ & 1691 cm^-1^, C=O stretching (anti-parallel β-sheet, amide I)  1525 cm^-1^, C-N stretching & N-H bending  1248 cm^-1^, C-N stretching & N-H bending (β-sheet, amide III) |
| **UICP11** | 95 | 8.55 | 542.25 | 543.25 [M+H]^+^  565.25 [M+Na]^+^  1085.45 [2M+H]^+^ | 1.86 (2H, q), 1.95 (2H, q), 2.07 (2H, t), 2.16 (2H, t), 4.02 (1H, t), 4.32 (1H, t), 5.29 (2H), 7.19-7.34 (10H, d & t) | 3028-3600 cm^-1^, O-H & N-H stretching  2980 cm^-1^, C-H aromatic stretching  2889 cm^-1^, C-H aliphatic stretching  1645 cm^-1^, C=O stretching (random coil, amide I)  1672 cm^-1^, C=O stretching (unordered/turn, amide I)  1554 cm^-1^, C-N stretching & N-H bending  1251 cm^-1^, C-N stretching & NH bending |
| **UICP12** | 97 | 2.05 | 540.00 | 541.35 [M+H]^+^  563.35 [M+Na]^+^  1085.45 [2M+H]^+^ | 1.26 (4H, t), 1.54 (4H, t), 1.63 (4H, t), 2.86 (4H, t), 4.13 (1H, t), 4.34 (1H, t), 5.04 (1H), 5.25 (1H), 7.22-7.42 (10H, d & t) | 3346 cm^-1^, O-H stretching  3250 cm^-1^, N-H stretching  2873 cm^-1^, C-H aliphatic stretching  1646 cm^-1^, C=O stretching (random coil, amide I)  1520 & 1539 cm^-1^, C-N stretching & N-H bending |
| **UICP13** | 98 | 2.79 | 670.71 | 671.35 [M+H]^+^ | 1.34 (4H, t), 1.53 (4H, t), 1.88 (2H, q), 2.01 (4H, t), 2.36 (4H, t), 2.80 (2H, t), 4.12 (1H, t), 4.29 (1H, t), 4.33 (1H, t), 5.05 (1H), 5.23 (1H), 7.20-7.41 (10H, d & t) | 3307 cm^-1^, O-H stretching  3275 cm^-1^, N-H stretching  3050 cm^-1^, C-H aromatic stretching  2976 cm^-1^, C-H aliphatic stretching  1634 cm^-1^ & 1690 cm^-1^, C=O stretching (anti-parallel β-sheet, amide I)  1538 cm^-1^, C-N stretching & N-H bending  1251 cm^-1^, C-N stretching & N-H bending (β-sheet, amide III) |
| **UICP14** | 96 | 8.82 | 670.71 | 669.25 [M-H]^-^ | 1.39-1.49 (4H, t), 1.86 (4H, q), 2.18 (2H, q), 2.32 (4H, t), 2.93 (2H, t), 3.96 (1H, t), 4.21 (1H, t), 4.39 (1H, t), 5.25 (1H), 5.37 (1H), 7.15-7.38 (10H, d & t) | 3350-3650 cm^-1^, O-H stretching  3260 cm^-1^, N-H stretching  3055 cm^-1^, C-H aromatic stretching  2937 cm^-1^, C-H aliphatic stretching  1624 cm^-1^ & 1695 cm^-1^, C=O stretching (anti-parallel β-sheet, amide I)  1530 cm^-1^, C-N stretching & N-H bending (β-sheet, amide II)  1250 cm^-1^, C-N stretching & N-H bending (β-sheet, amide III) |
| **UICP15** | 97 | 2.80 | 669.76 | 668.30 [M-H]^-^ | N/A | 3660 cm^-1^, O-H & N-H stretching  3080 cm^-1^, C-H aromatic stretching  2980 cm^-1^, C-H aliphatic stretching  1678 cm^-1^, C=O stretching (unordered, amide I)  1541 cm^-1^, C-N stretching & NH bending  1251 cm^-1^, C-N stretching & NH bending |
| **UICP16** | 96 | 3.70 | 669.77 | 668.30 [M-H]^-^ | 1.28 (4H, t), 1.52 (4H, t), 1.78-2.20 (6H, q), 2.18 (2H, q), 2.43-2.82 (6H, t), 4.07 (1H, t), 4.22 (1H, t), 4.43 (1H, t), 5.22 (1H), 5.35 (1H), 7.18-7.37 (10H, d & t) | 3322-4000 cm^-1^, O-H stretching  3274 cm^-1^, N-H stretching  3060 cm^-1^, C-H aromatic stretching  2943 cm^-1^, C-H aliphatic stretching  1624 cm^-1^ cm^-1^, C=O stretching (anti-parallel β-sheet, amide I)  1678 cm^-1^, C=O stretching (unordered/turn, amide I)  1559 cm^-1^, C-N stretching & N-H bending  1248 cm^-1^, C-N stretching & N-H bending (β-sheet, amide III) |

*Stock solution of each peptide was prepared by dissolving the peptide powder in HPLC water at a concentration of 1 mg/mL, which was then diluted 10X to a final concentration of 100 µg/mL. A Flexar autosampler system was used for the chromatographic separation by injecting 20 µL of the diluted peptide solutions on the RP-HPLC analytical scale Phenomenex Jupiter 4µ Proteo column 90A° (150 × 4.6 mm), at a flow rate of 2 mL/min. Isocratic separation was performed using 90% water (H_2_O) / 10% acetonitrile (CH_3_CN) (all solvents contained 0.05 % of trifluoroacetic acid) over 10 min. A Flexar UV detector was used for detection of peptides at a wavelength λ 220 nm. **Peptide samples were prepared by dissolving peptide powder in 50% H_2_O/50% CH_3_CN at a concentration of 1mg/mL, which was then analysed by ESI-MS Shimazu 2020. **^¶^** Peptide samples were prepared by dissolving peptide powder in D_2_O at a concentration of 1mg/mL, which was then analysed by a JEOL ECZR 600 MHz (equipped with a ROYALprobe). ^#^All peptide samples were prepared at 45 mM concentration and measured at pH 4.5, with exception of UICP11 which is water insoluble at this pH and was solubilised at pH 7 before measurements. Samples were scanned using a Bruker Alpha ATR-FTIR transmittance was recorded between 4000 and 400 cm^-1^. N/A: not analysed.


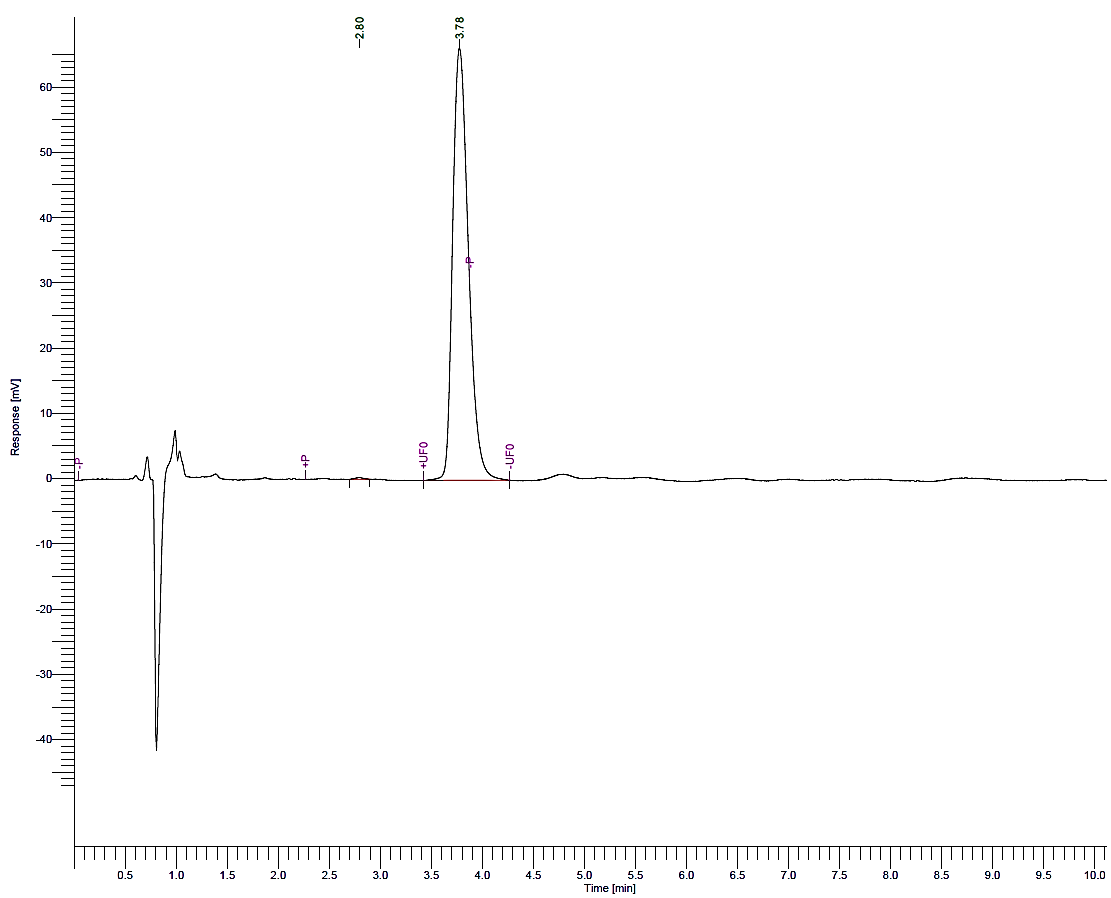


**Characterisation Figure C1.** RP-HPLC trace for UICP1 showing a main peak at retention time 3.78 min.


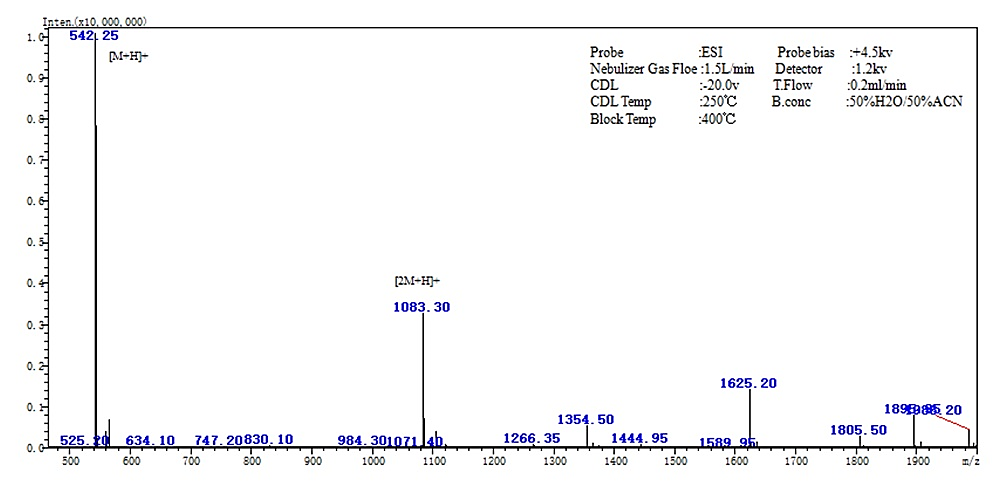


**Characterisation Figure C2.** ESI-MS spectrum for UICP1 showing a main peak of m/z 542.25 [M+H]^+^.

**1521 cm^-1^**

**C-N stretching & N-H bending**

**1689 cm^-1^ C=O stretching**

**2946 cm^-1^ C-H aliphatic stretching**

**3050 cm^-1^ C-H aromatic stretching**

**3440 cm^-1^ O-H & N-H stretching**

**1645 cm^-1^ C=O stretching**

**1247 cm^-1^**

**C-N stretching & N-H bending**

**1616 cm^-1^ C=O stretching**

**Characterisation Figure C3.** ATR-FTIR spectrum for UICP1 showing the characteristic functional group peaks.


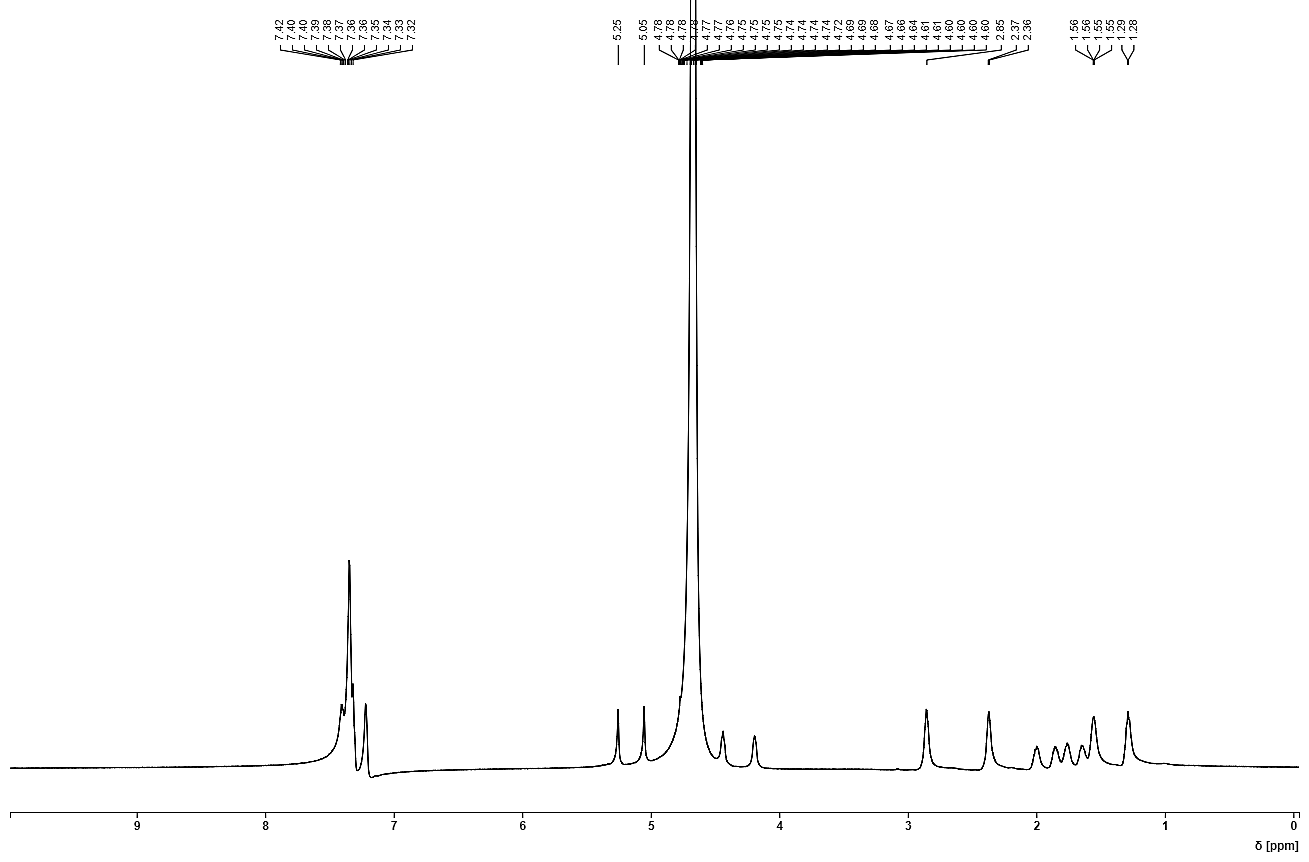


**Characterisation Figure C4.** ^1^H NMR spectrum for UICP1 in D_2_O showing the different proton peaks.


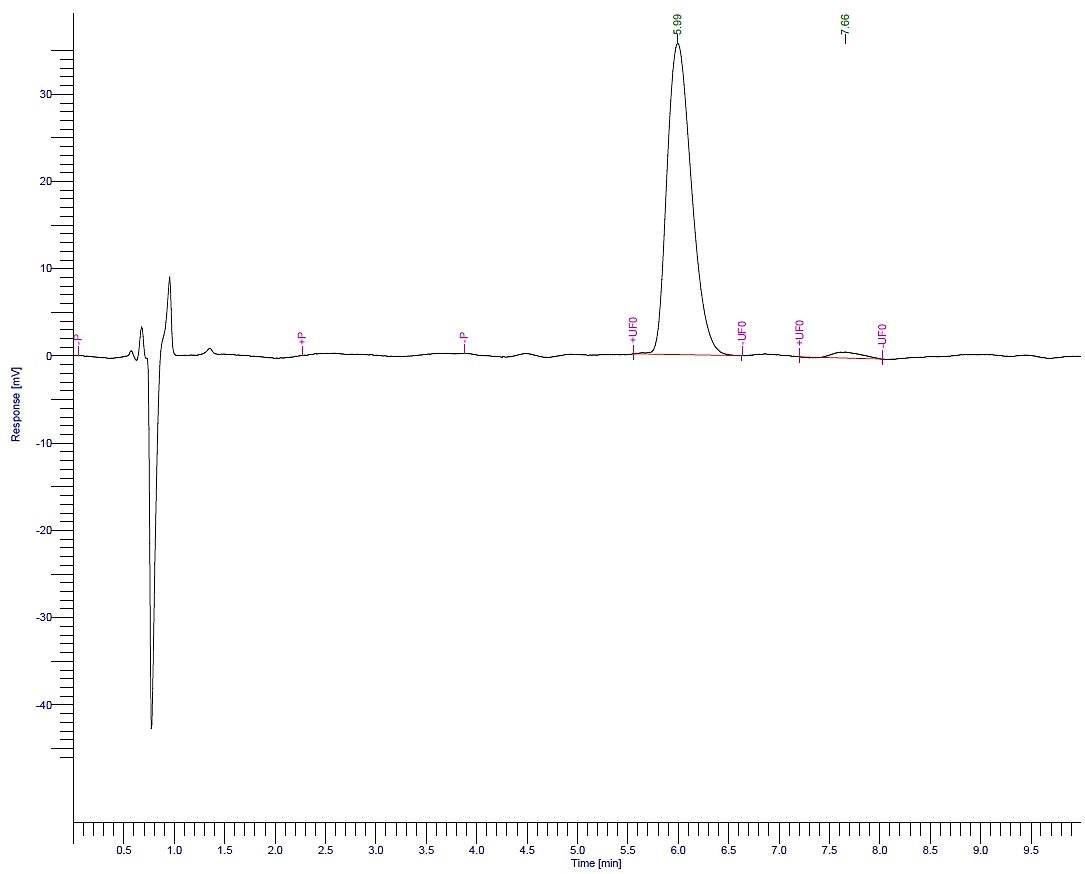


**Characterisation Figure C5.** RP-HPLC trace for UICP2 showing a main peak at retention time 5.99 min.


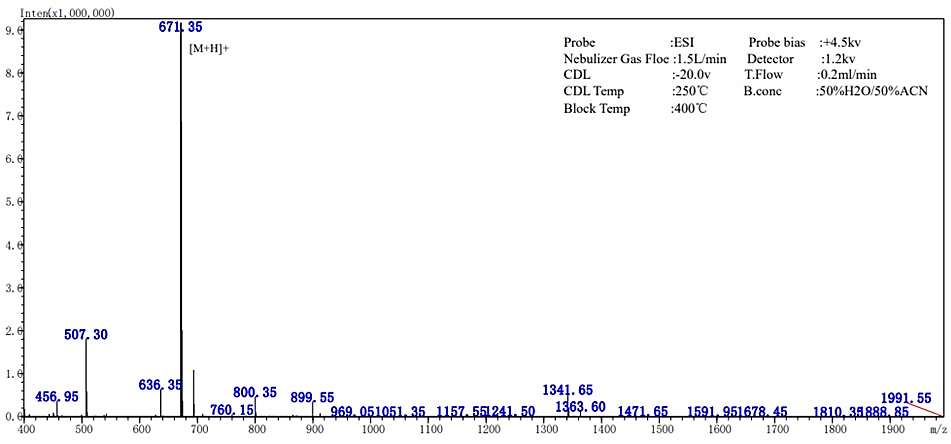


**Characterisation Figure C6.** ESI-MS spectrum for UICP2 showing a main peak of m/z 671.35 [M+H]^+^.

**1248 cm^-1^**

**C-N stretching & N-H bending**

**1628 cm^-1^ C=O stretching**

**1530 cm^-1^**

**C-N stretching & N-H bending**

**1649 cm^-1^ C=O stretching**

**1689 cm^-1^ C=O stretching**

**2935 cm^-1^ C-H aliphatic stretching**

**3280 cm^-1^**

**N-H stretching**

**3350-3634 cm^-1^**

**O-H stretching**

**3046 cm^-1^ CH aromatic stretching**

**Characterisation Figure C7.** ATR-FTIR spectrum for UICP2 showing the characteristic functional group peaks.


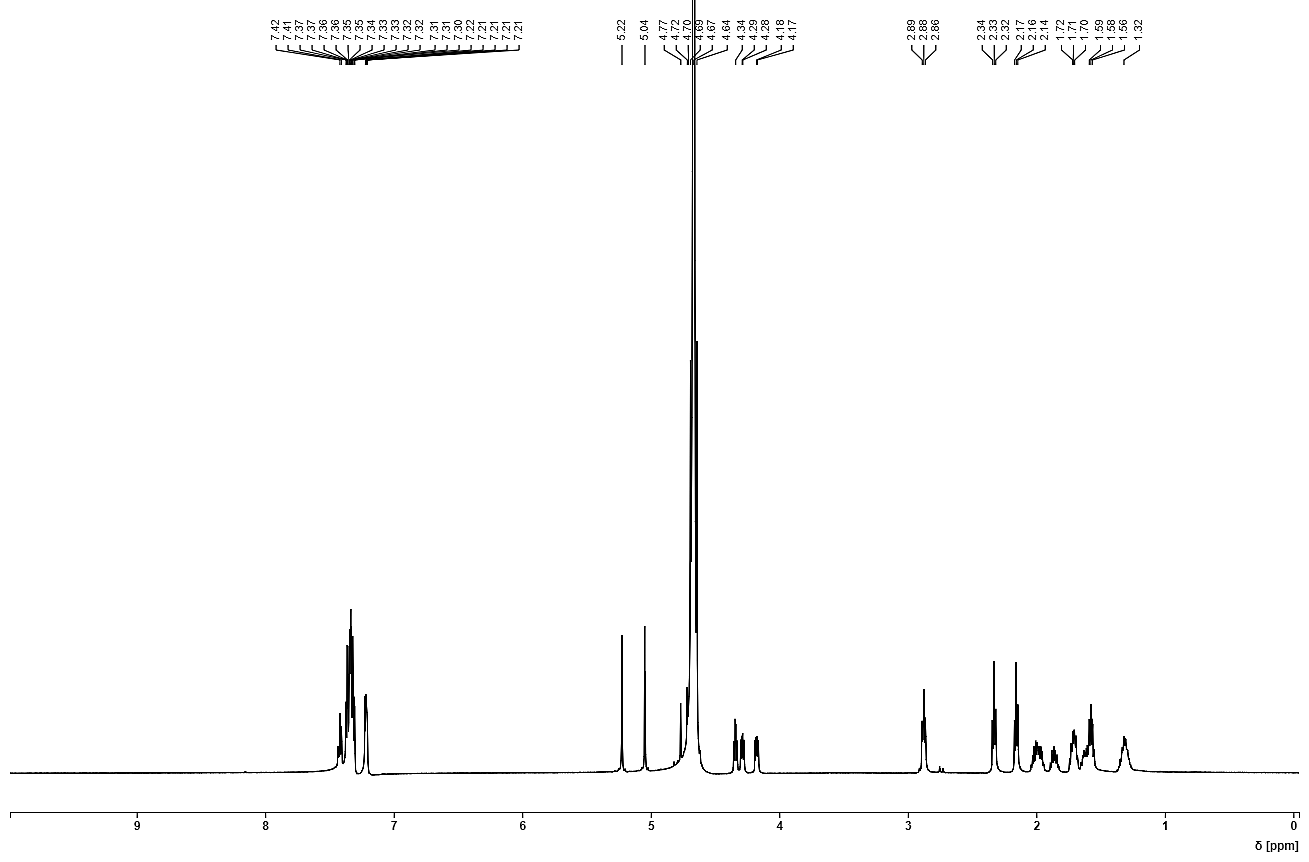


**Characterisation Figure C8.** ^1^H NMR spectrum for UICP2 in D_2_O showing the different proton peaks.


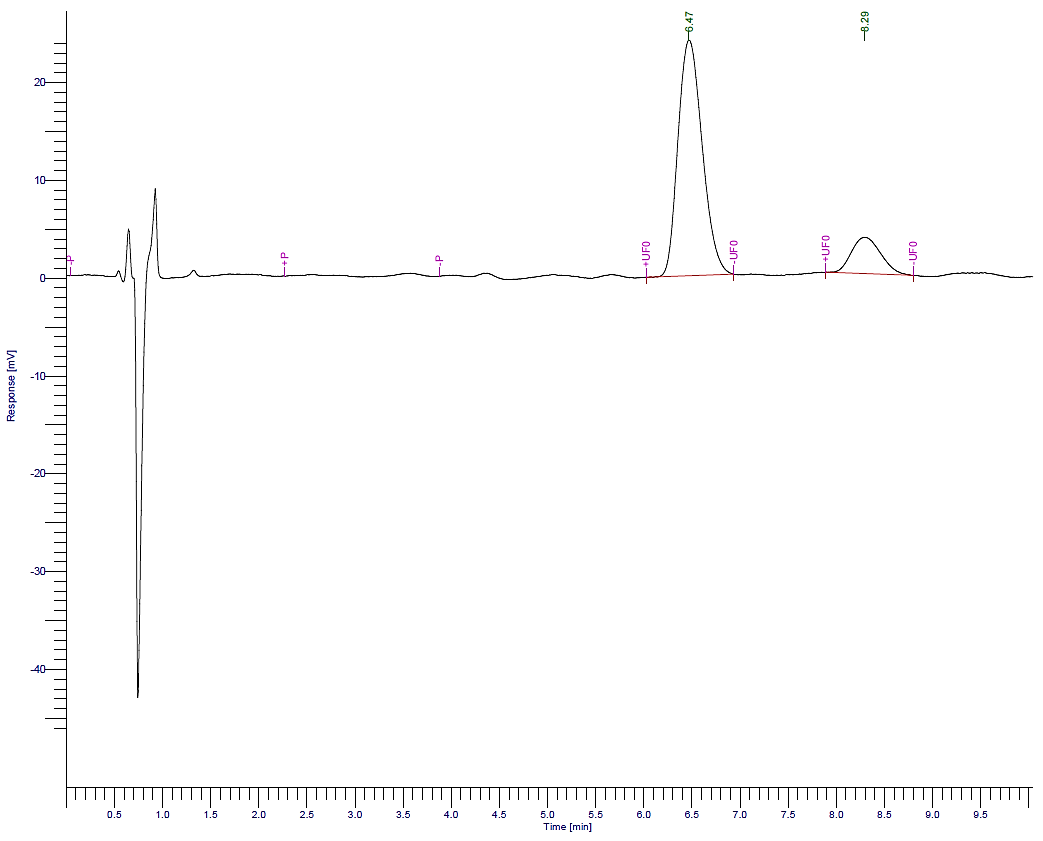


**Characterisation Figure C9.** RP-HPLC trace for UICP3 showing a main peak at retention time 6.47 min.


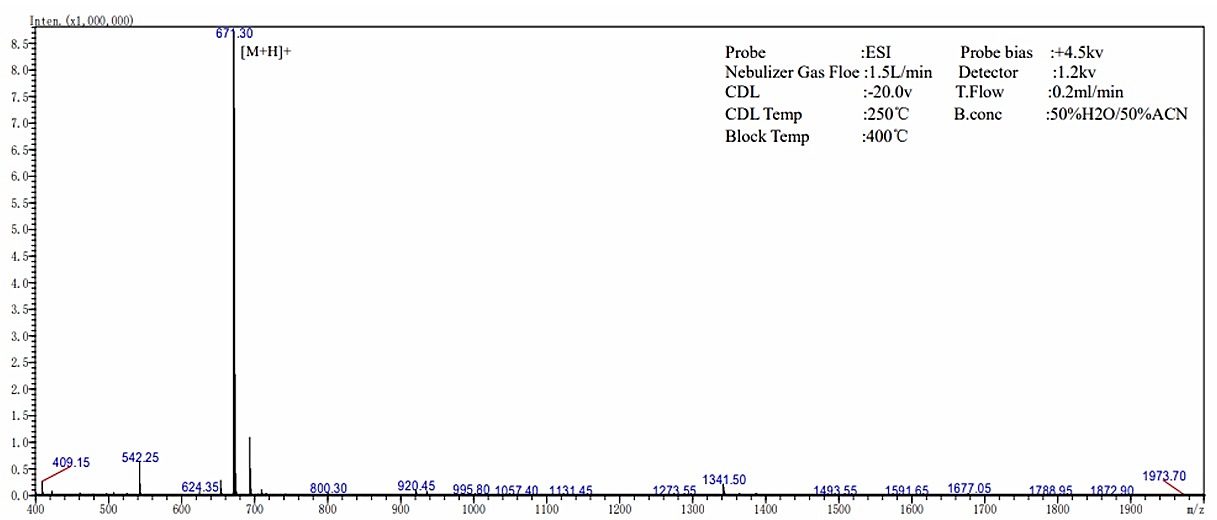


**Characterisation Figure C10.** ESI-MS spectrum for UICP3 showing a main peak of m/z 671.35 [M+H]^+^.

**1535 cm^-1^**

**C-N stretching & N-H bending**

**1626 cm^-1^ C=O stretching**

**1670 cm^-1^ C=O stretching**

**2952 cm^-1^ C-H aliphatic stretching**

**3046 cm^-1^ C-H aromatic stretching**

**3308 cm^-1^ O-H & N-H stretching**

**1238 cm^-1^**

**C-N stretching & N-H bending**

**Characterisation Figure C11.** ATR-FTIR spectrum for UICP3 showing the characteristic functional group peaks.


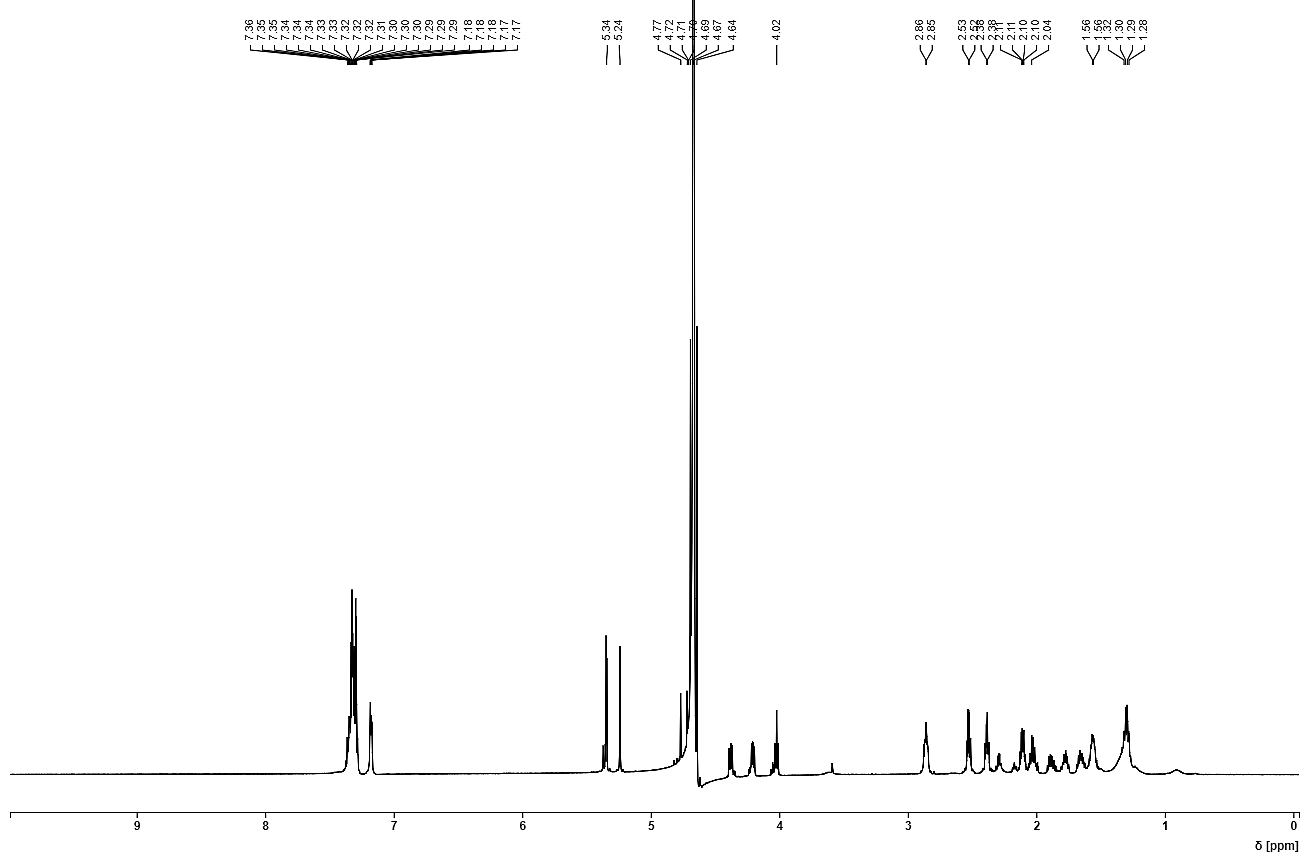


**Characterisation Figure C12.** ^1^H NMR spectrum for UICP3 in D_2_O showing the different proton peaks.


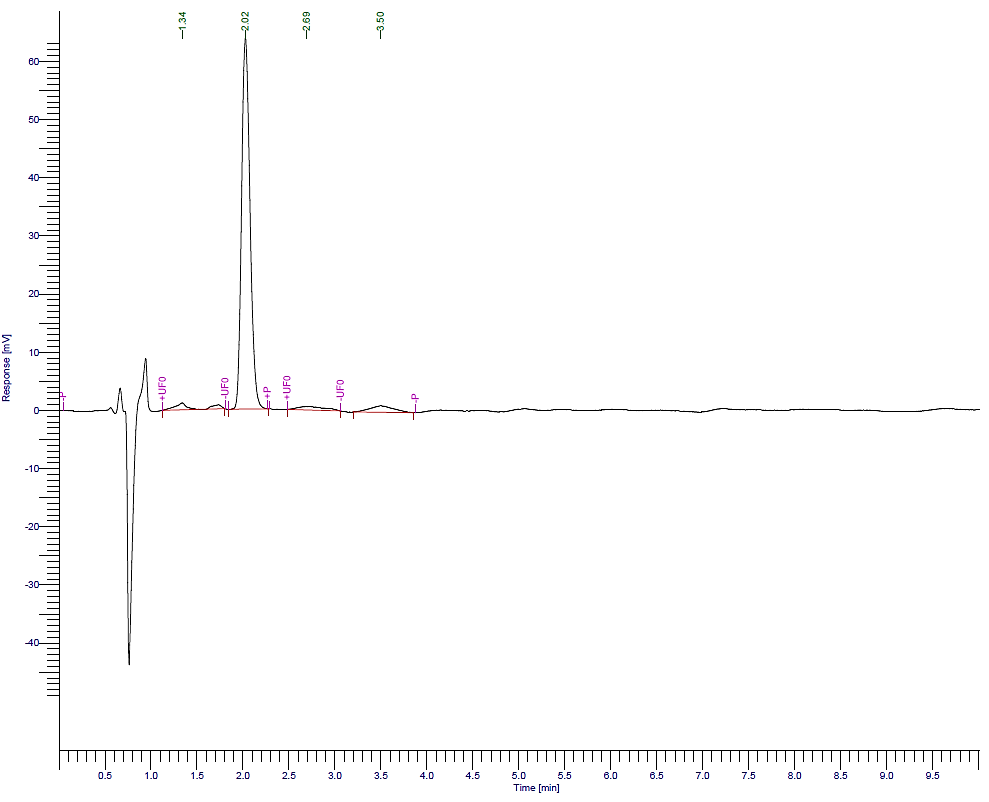


**Characterisation Figure C13.** RP-HPLC trace for UICP4 showing a main peak at retention time 2.02 min.


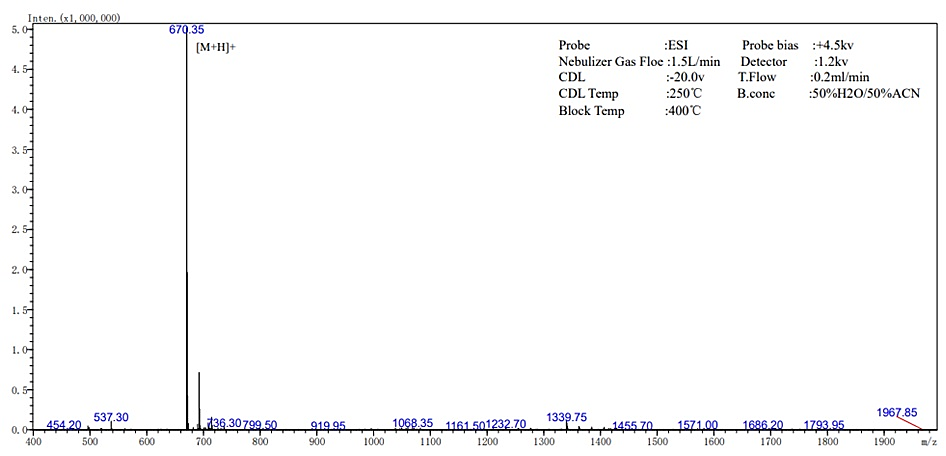


**Characterisation Figure C14.** ESI-MS spectrum for UICP4 showing a main peak of m/z 670.35 [M+H]^+^.

**1650 cm^-1^ C=O stretching**

**2976 cm^-1^ C-H aliphatic stretching**

**3316-4000 cm^-1^**

**O-H & N-H stretching**

**1258 cm^-1^**

**C-N stretching & N-H bending**

**1676 cm^-1^ C=O stretching**

**3055 cm^-1^ C-H aromatic stretching**

**1550 cm^-1^**

**C-N stretching & N-H bending**

**Characterisation Figure C15.** ATR-FTIR spectrum for UICP4 showing the characteristic functional group peaks.


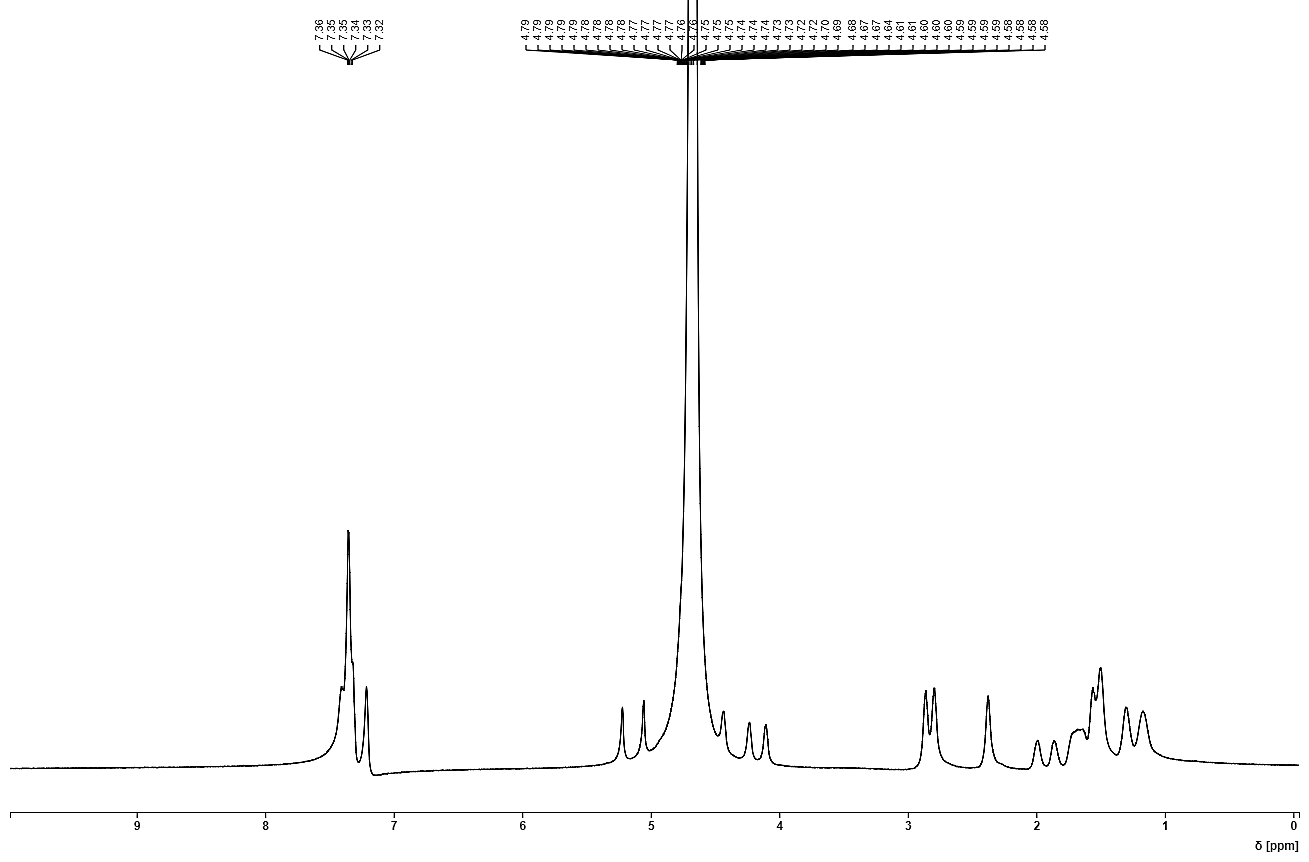


**Characterisation Figure C16.** ^1^H NMR spectrum for UICP4 in D_2_O showing the different proton peaks.


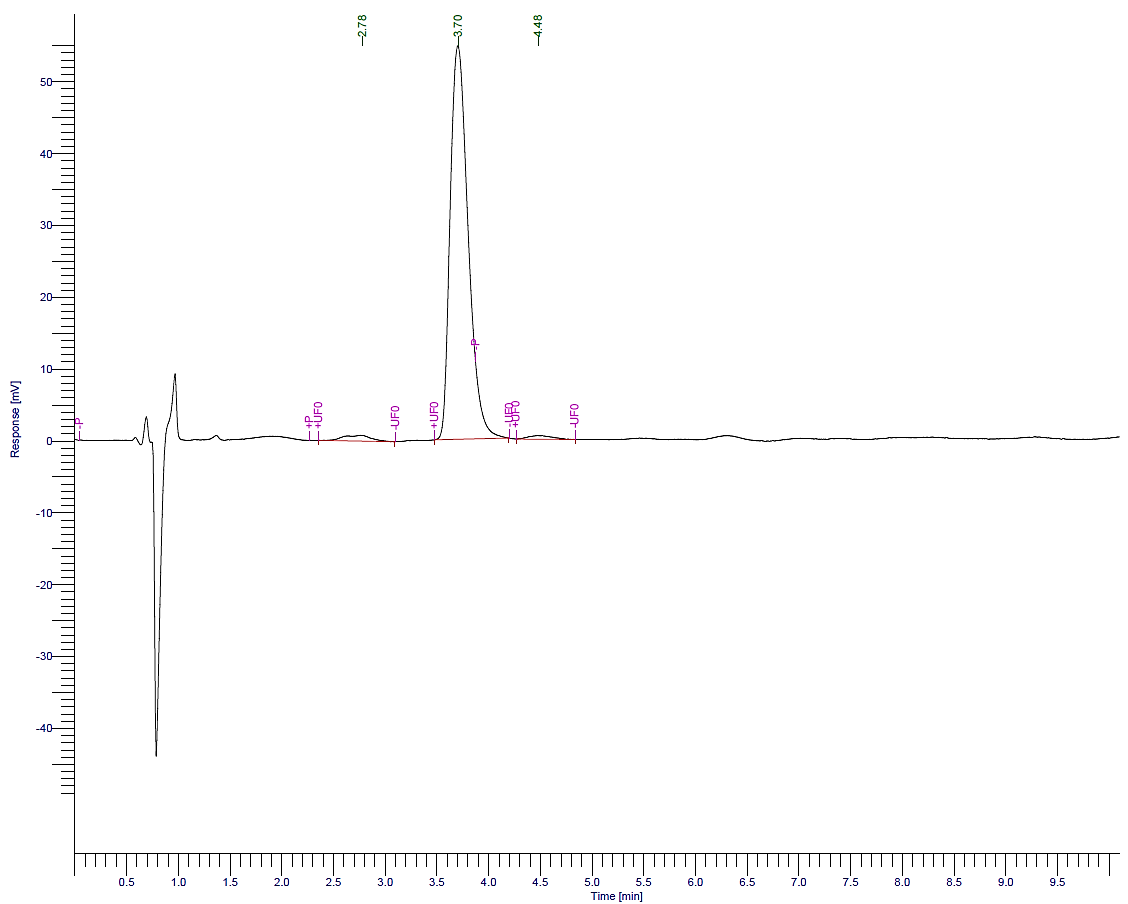


**Characterisation Figure C17.** RP-HPLC trace for UICP5 showing a main peak at retention time 3.7 min.


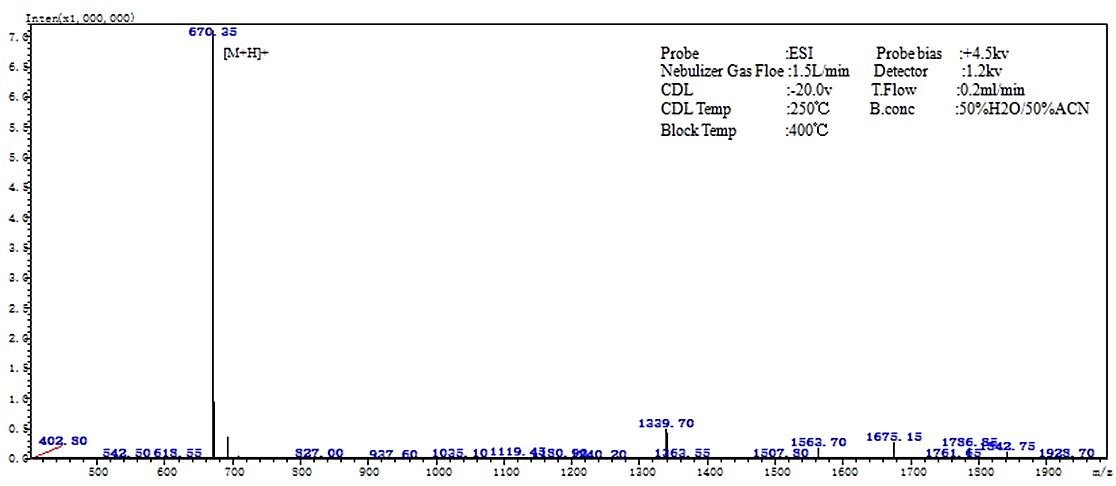


**Characterisation Figure C18.** ESI-MS spectrum for UICP5 showing a main peak of m/z 670.35 [M+H]^+^.

**2928 cm^-1^ C-H aliphatic stretching**

**3034 cm^-1^ C-H aromatic stretching**

**3360-3628**

**cm^-1^**

**O-H**

**stretching**

**1628 cm^-1^ C=O stretching**

**1554, 1527 cm^-1^**

**C-N stretching & N-H bending**

**1266 cm^-1^**

**C-N stretching & N-H bending**

**1675 cm^-1^ C=O stretching**

**3261 cm^-1^**

**N-H**

**stretching**

**Characterisation Figure C19.** ATR-FTIR spectrum for UICP5 showing the characteristic functional group peaks.


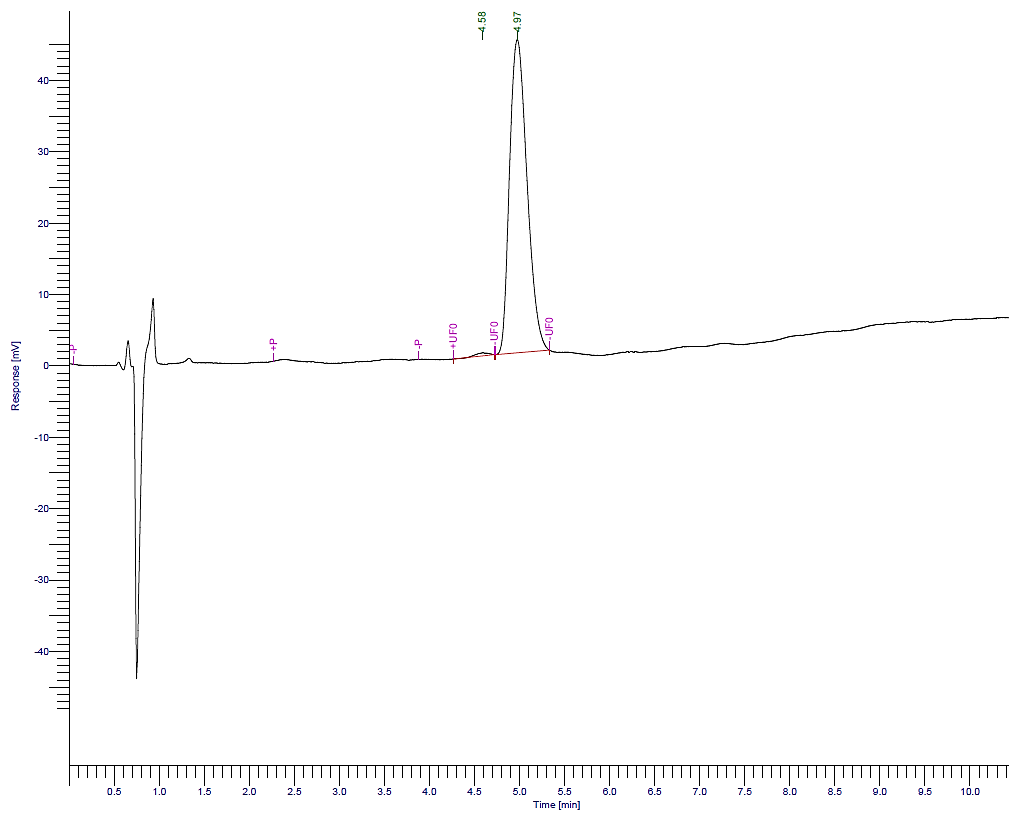


**Characterisation Figure C20.** RP-HPLC trace for UICP6 showing a main peak at retention time 4.97 min.


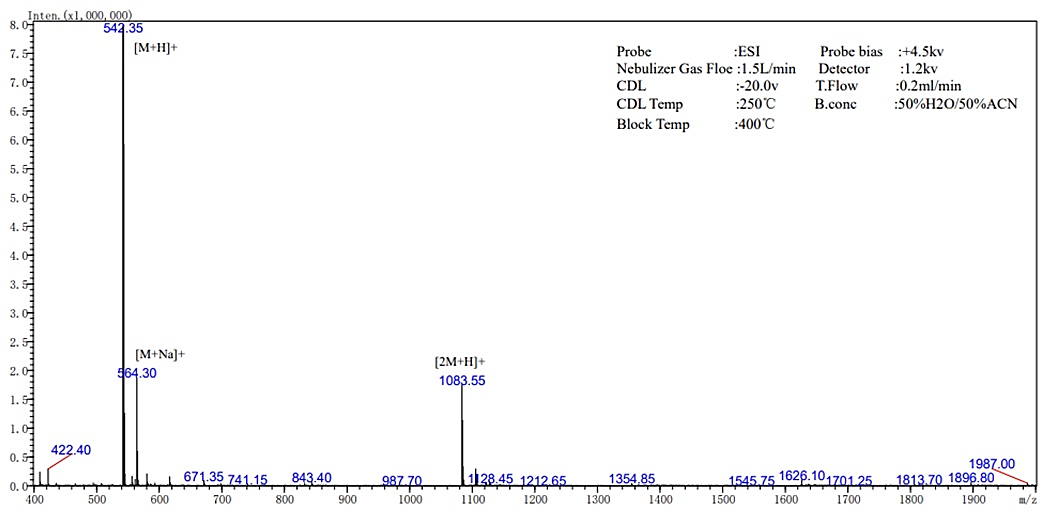


**Characterisation Figure C21.** ESI-MS spectrum for UICP6 showing a main peak of m/z 542.35 [M+H]^+^.

**1257 cm^-1^**

**C-N stretching & N-H bending**

**1523 cm^-1^**

**C-N stretching & N-H bending**

**1618 cm^-1^ C=O stretching**

**1647 cm^-1^ C=O stretching**

**1689 cm^-1^ C=O stretching**

**2976 cm^-1^ C-H aliphatic stretching**

**3055 cm^-1^ C-H aromatic stretching**

**3286 cm^-1^**

**N-H stretching**

**3340-3634 cm^-1^**

**O-H stretching**

**Characterisation Figure C22.** ATR-FTIR spectrum for UICP6 showing the characteristic functional group peaks.


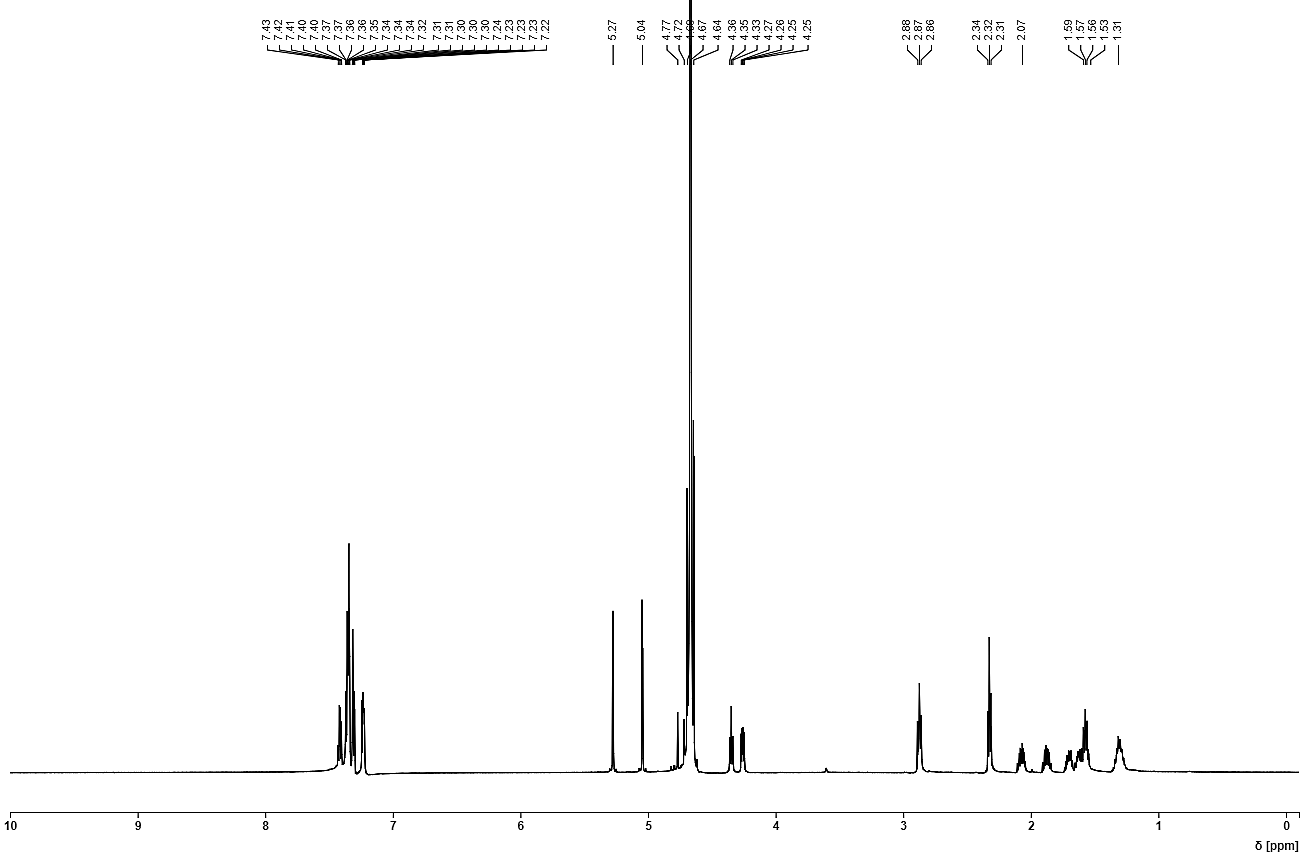


**Characterisation Figure C23.** ^1^H NMR spectrum for UICP6 in D_2_O showing the different proton peaks.


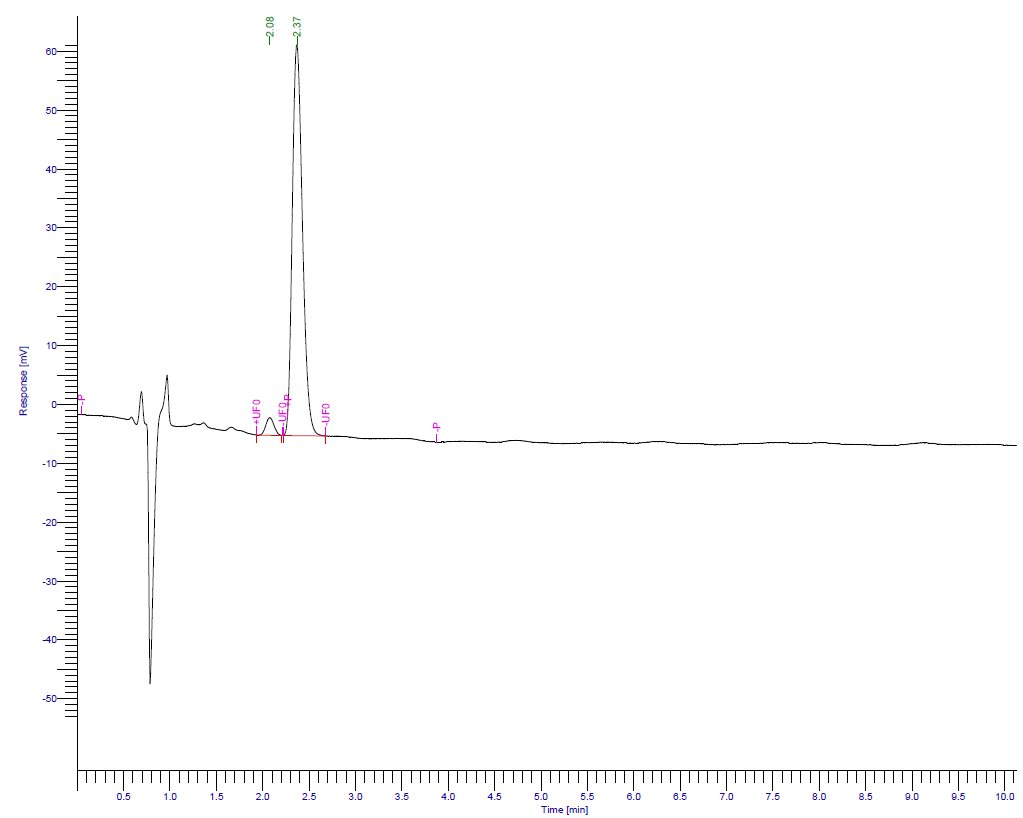


**Characterisation Figure C24.** RP-HPLC trace for UICP7 showing a main peak at retention time 2.37 min.


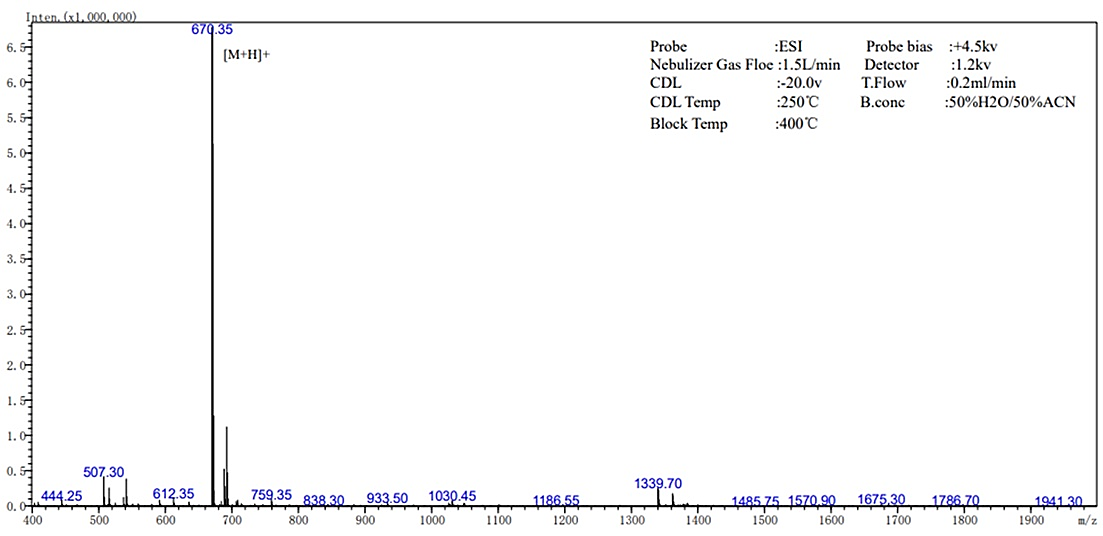


**Characterisation Figure C25.** ESI-MS spectrum for UICP7 showing a main peak of m/z 670.35 [M+H]^+^.

**1254 cm^-1^**

**C-N stretching & N-H bending**

**1547 cm^-1^**

**C-N stretching & N-H bending**

**1677 cm^-1^ C=O stretching**

**2989 cm^-1^ C-H aliphatic stretching**

**3067 cm^-1^ C-H aromatic stretching**

**3668 cm^-1^ O-H & N-H stretching**

**Characterisation Figure C26.** ATR-FTIR spectrum for UICP7 showing the characteristic functional group peaks.


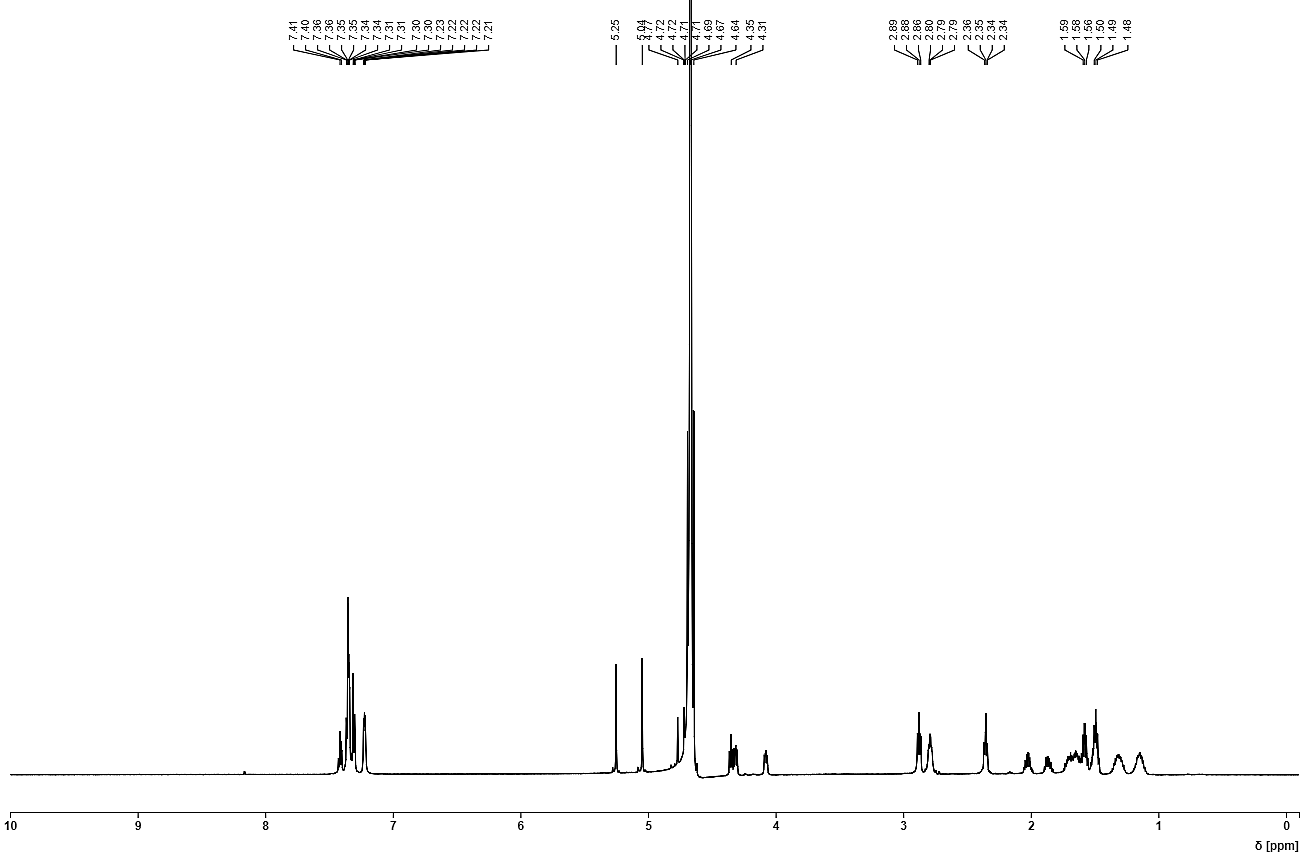


**Characterisation Figure C27.** ^1^H NMR spectrum for UICP7 in D_2_O showing the different proton peaks.


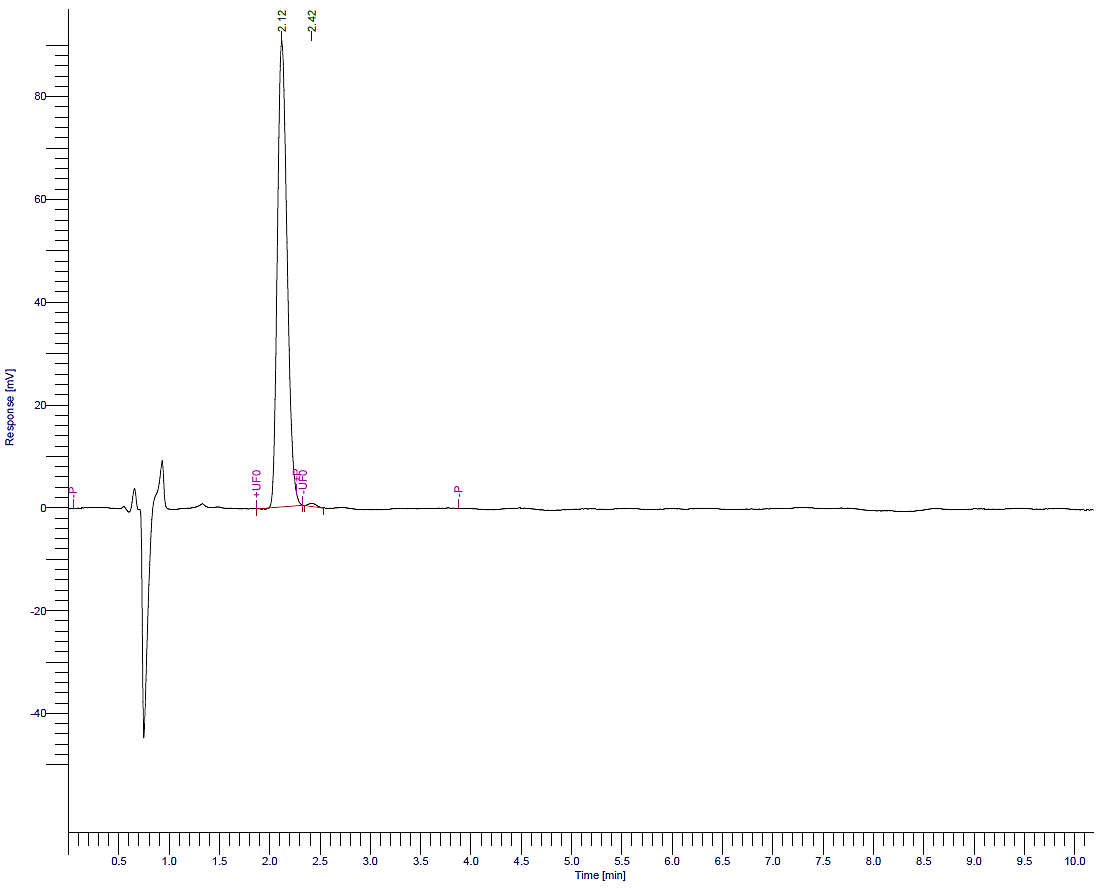


**Characterisation Figure C28.** RP-HPLC trace for UICP8 showing a main peak at retention time 2.12 min.


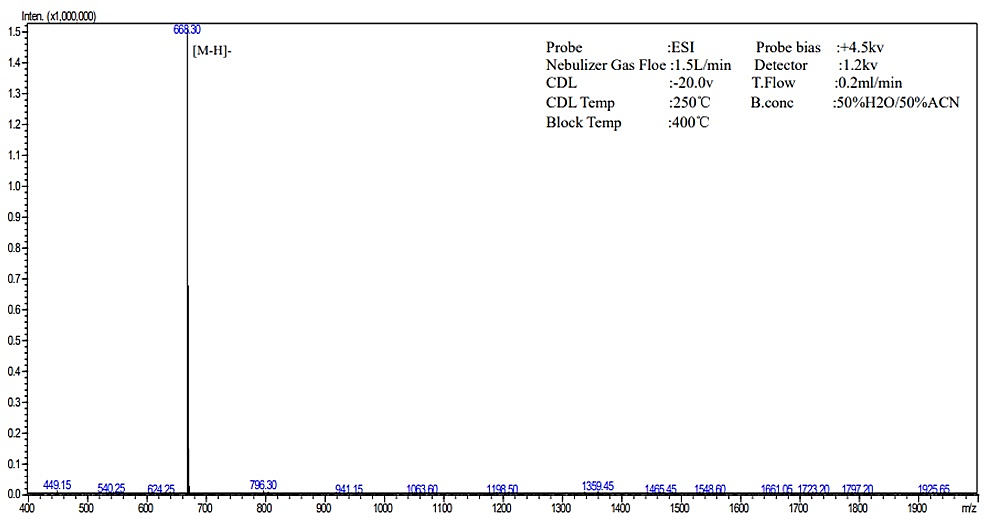


**Characterisation Figure C29.** ESI-MS spectrum for UICP8 showing a main peak of m/z 668.30 [M-H]^-^.

**1650 cm^-1^**

**C=O stretching**

**1623 cm^-1^**

**C=O stretching**

**1680 cm^-1^**

**C=O stretching**

**1557 cm^-1^**

**C-N stretching & N-H bending**

**2973 cm^-1^ C-H aliphatic stretching**

**3270 cm^-1^**

**N-H stretching**

**3043 cm^-1^ C-H aromatic stretching**

**3386-3637 cm^-1^**

**O-H stretching**

**Characterisation Figure C30.** ATR-FTIR spectrum for UICP8 showing the characteristic functional group peaks.


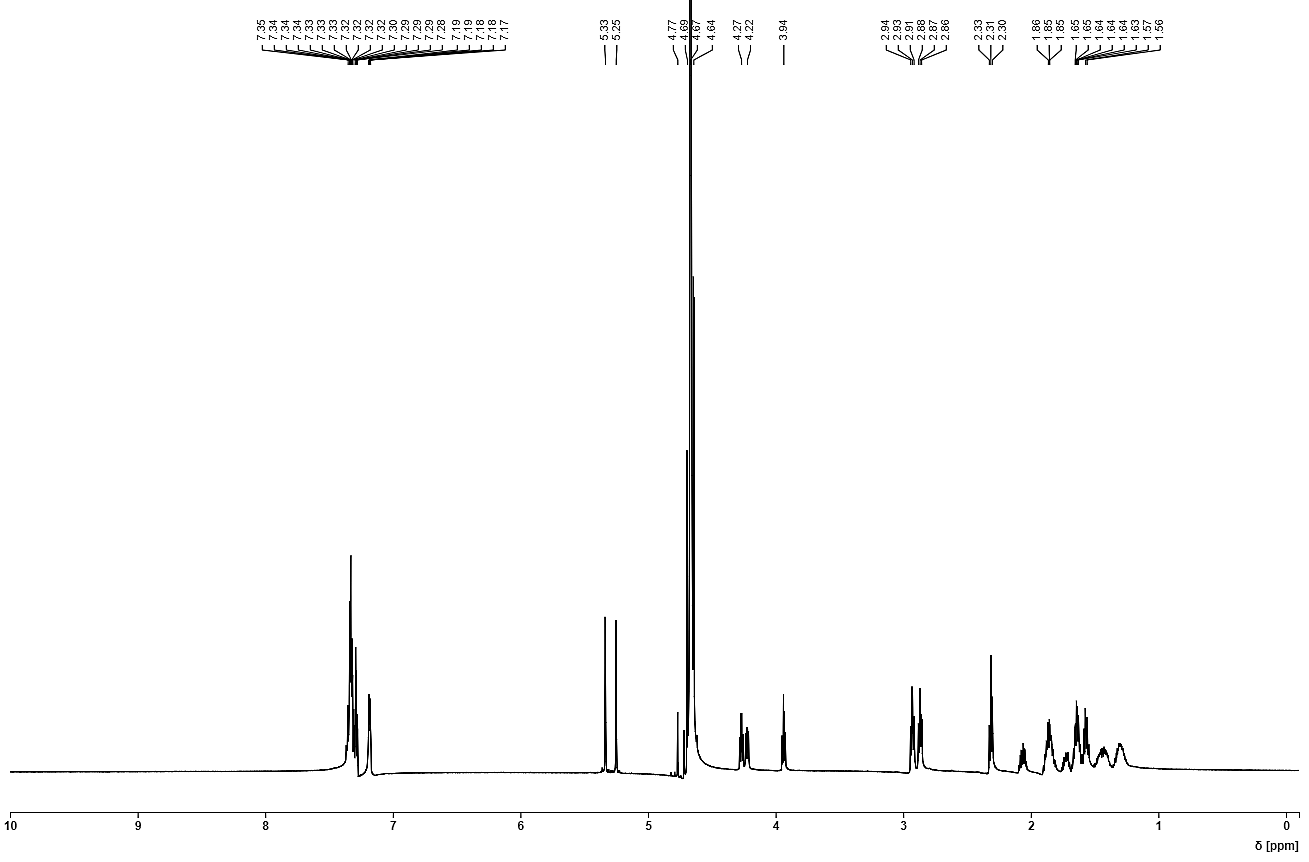


**Characterisation Figure C31.** ^1^H NMR spectrum for UICP8 in D_2_O showing the different proton peaks.


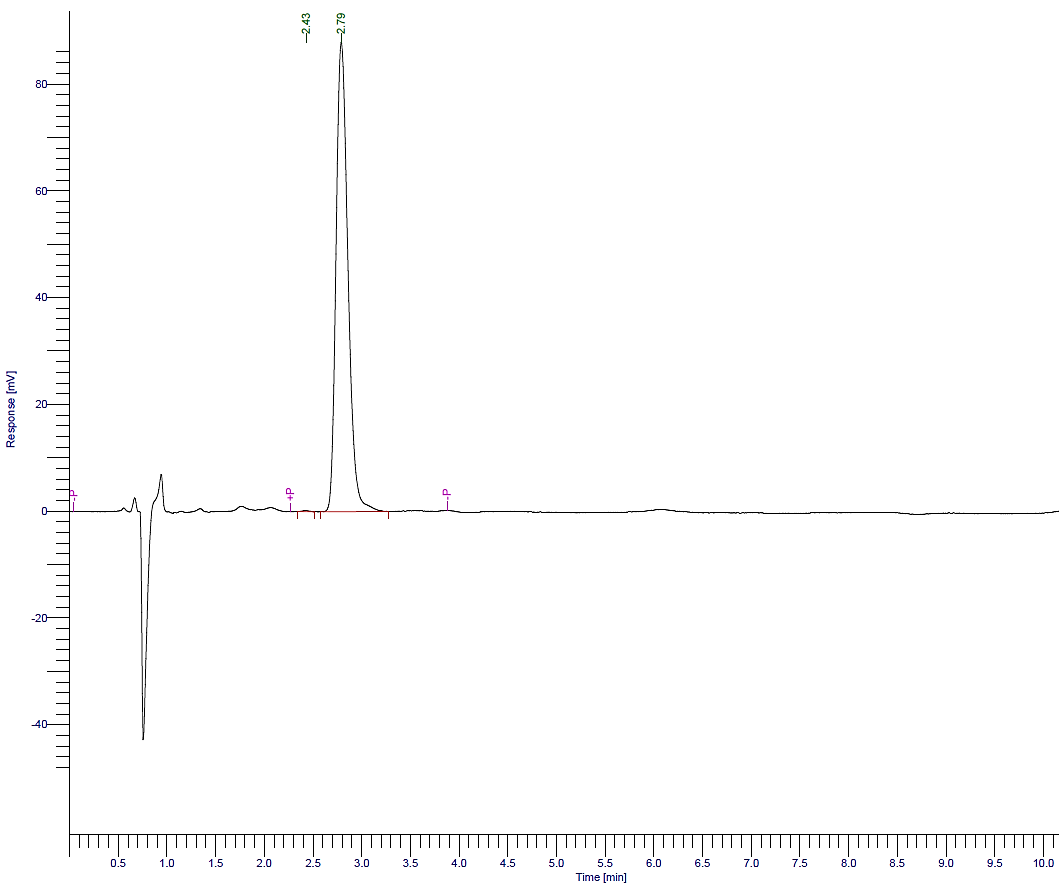


**Characterisation Figure C32.** RP-HPLC trace for UICP9 showing a main peak at retention time 2.79 min.


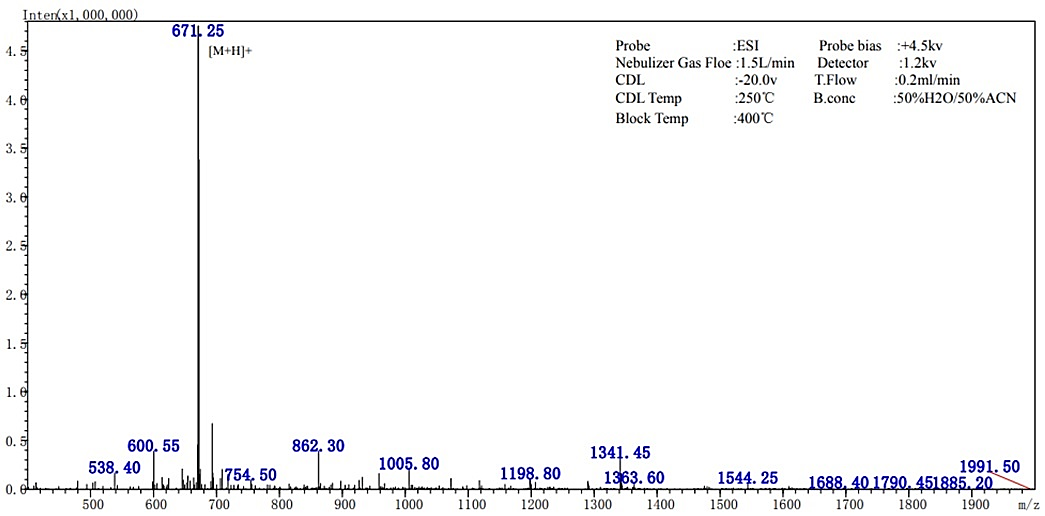


**Characterisation Figure C33.** ESI-MS spectrum for UICP9 showing a main peak of m/z 671.25 [M+H]^+^.

**1650 cm^-1^ C=O stretching**

**2934 cm^-1^ C-H aliphatic stretching**

**3050 cm^-1^ C-H aromatic stretching**

**3286 cm^-1^ N-H stretching**

**3350-4000 cm^-1^**

**O-H stretching**

**1523 cm^-1^**

**C-N stretching & N-H bending**

**1624 cm^-1^ C=O stretching**

**1689 cm^-1^ C=O stretching**

**1243 cm^-1^**

**C-N stretching & N-H bending**

**Characterisation Figure C34.** ATR-FTIR spectrum for UICP9 showing the characteristic functional group peaks.


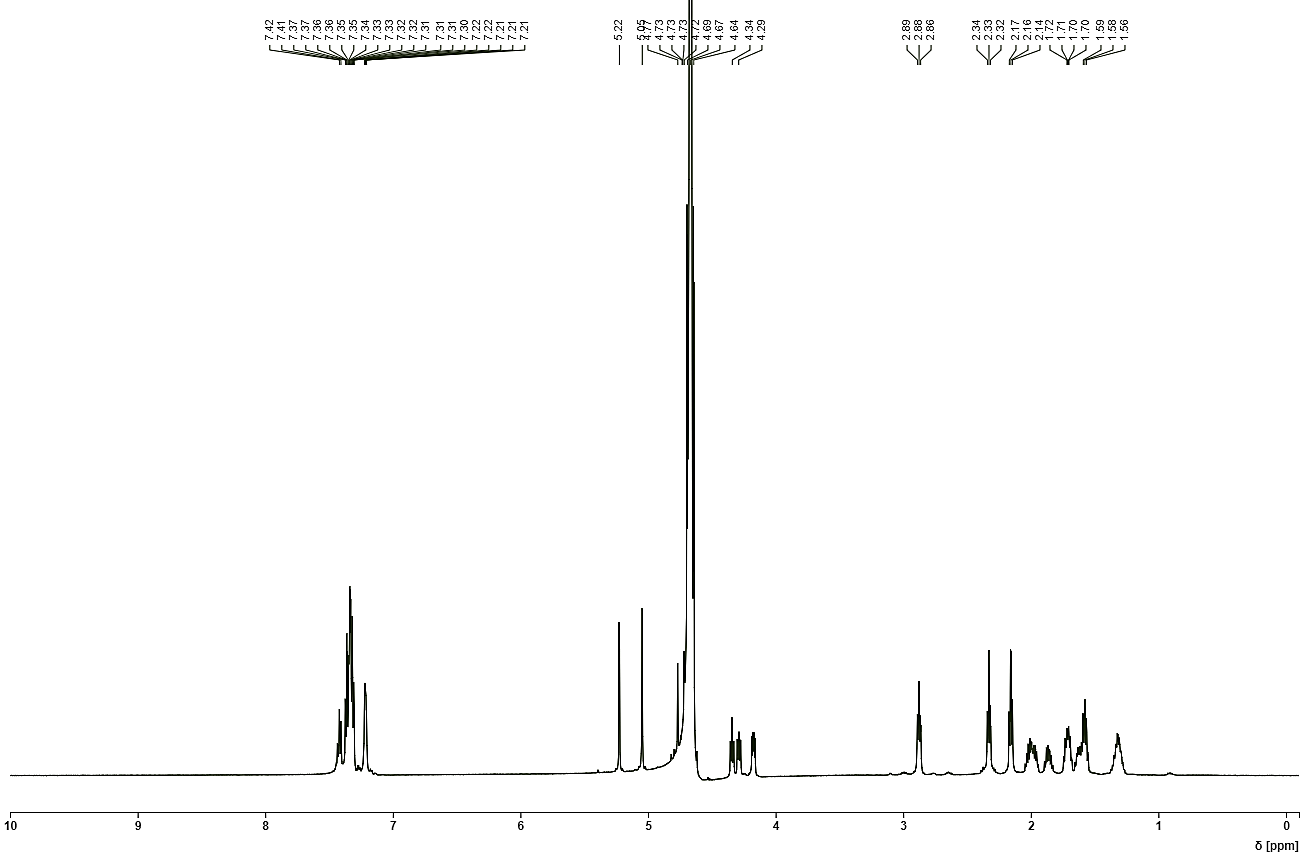


**Characterisation Figure C35.** ^1^H NMR spectrum for UICP9 in D_2_O showing the different proton peaks.


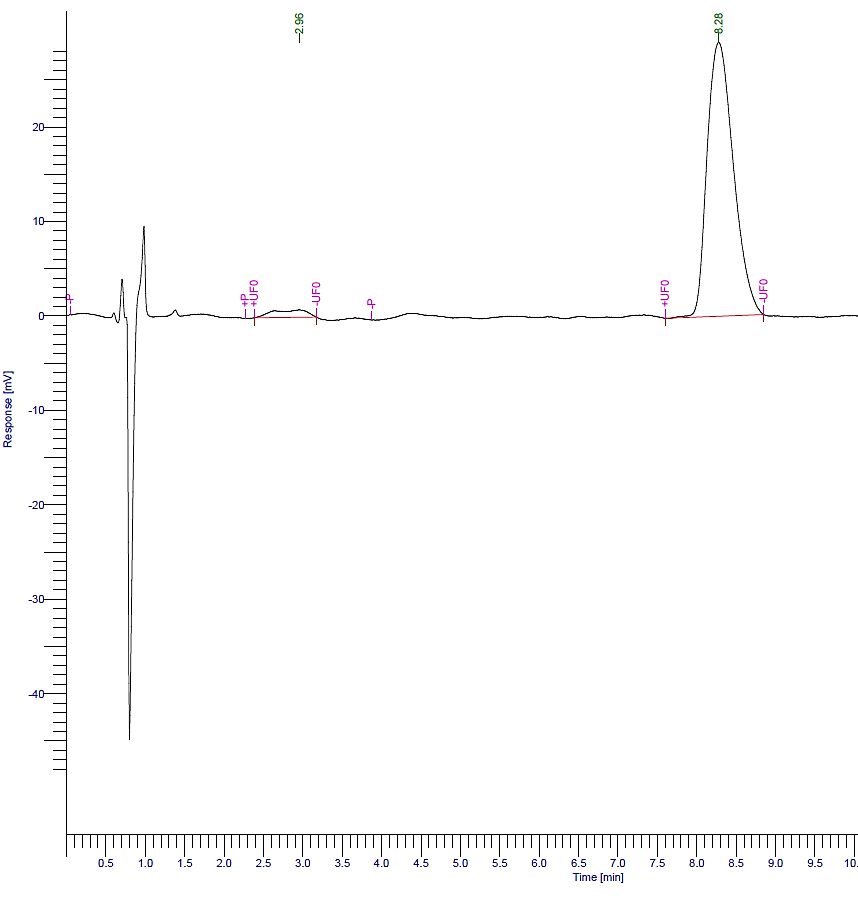


**Characterisation Figure C36.** RP-HPLC trace for UICP10 showing a main peak at retention time 8.28 min.


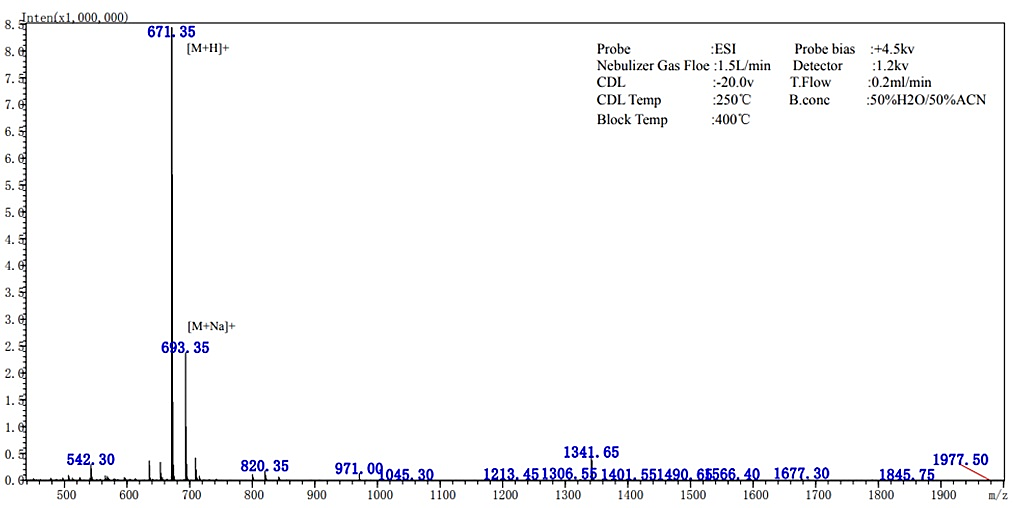


**Characterisation Figure C37.** ESI-MS spectrum for UICP10 showing a main peak of m/z 671.35 [M+H]^+^.

**3440 cm^-1^ O-H stretching**

**3277 cm^-1^ N-H stretching**

**3050 cm^-1^ C-H aromatic stretching**

**2943 cm^-1^ C-H aliphatic stretching**

**1691 cm^-1^ C=O stretching**

**1248 cm^-1^**

**C-N stretching & N-H bending**

**1525 cm^-1^**

**C-N stretching & N-H bending**

**1625 cm^-1^ C=O stretching**

**Characterisation Figure C38.** ATR-FTIR spectrum for UICP10 showing the characteristic functional group peaks.


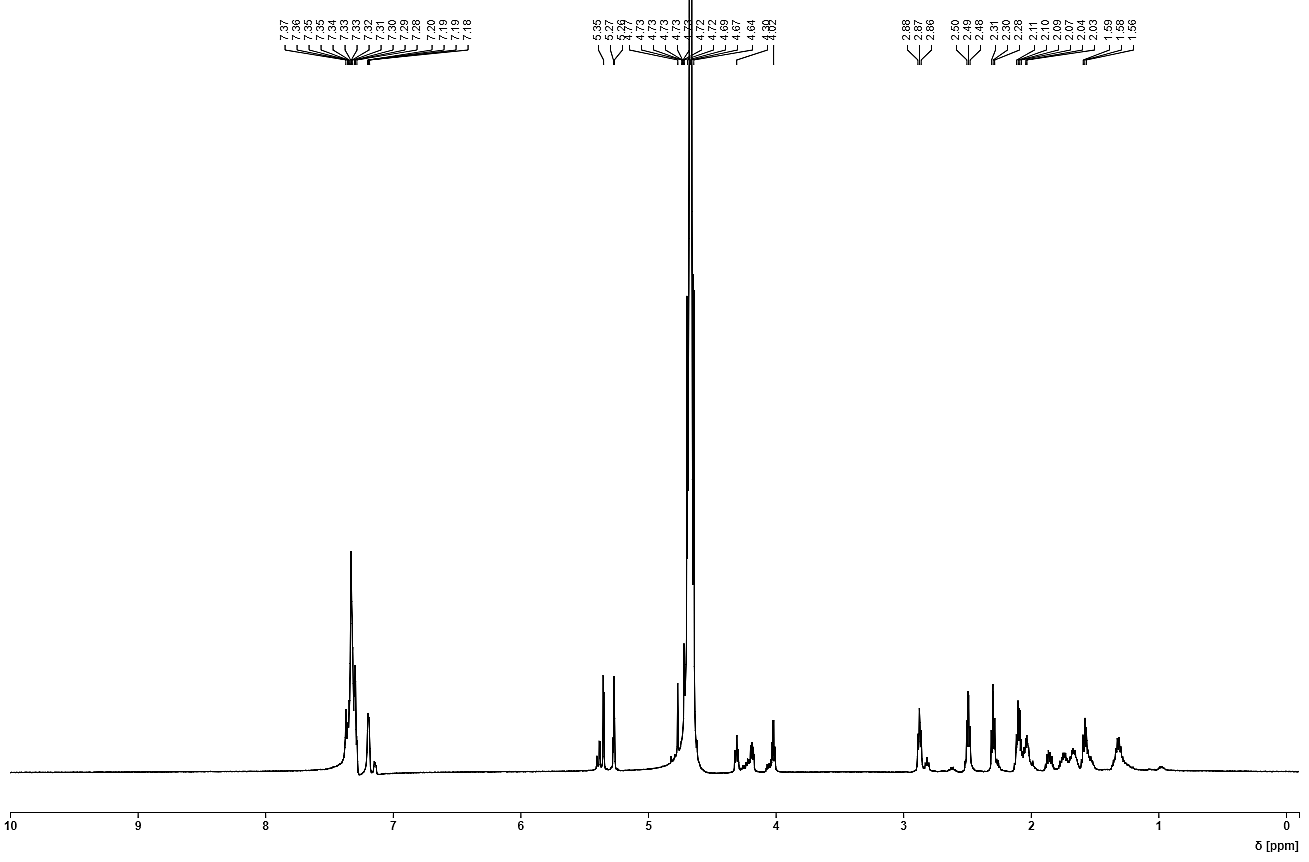


**Characterisation Figure C39.** ^1^H NMR spectrum for UICP10 in D_2_O showing the different proton peaks.


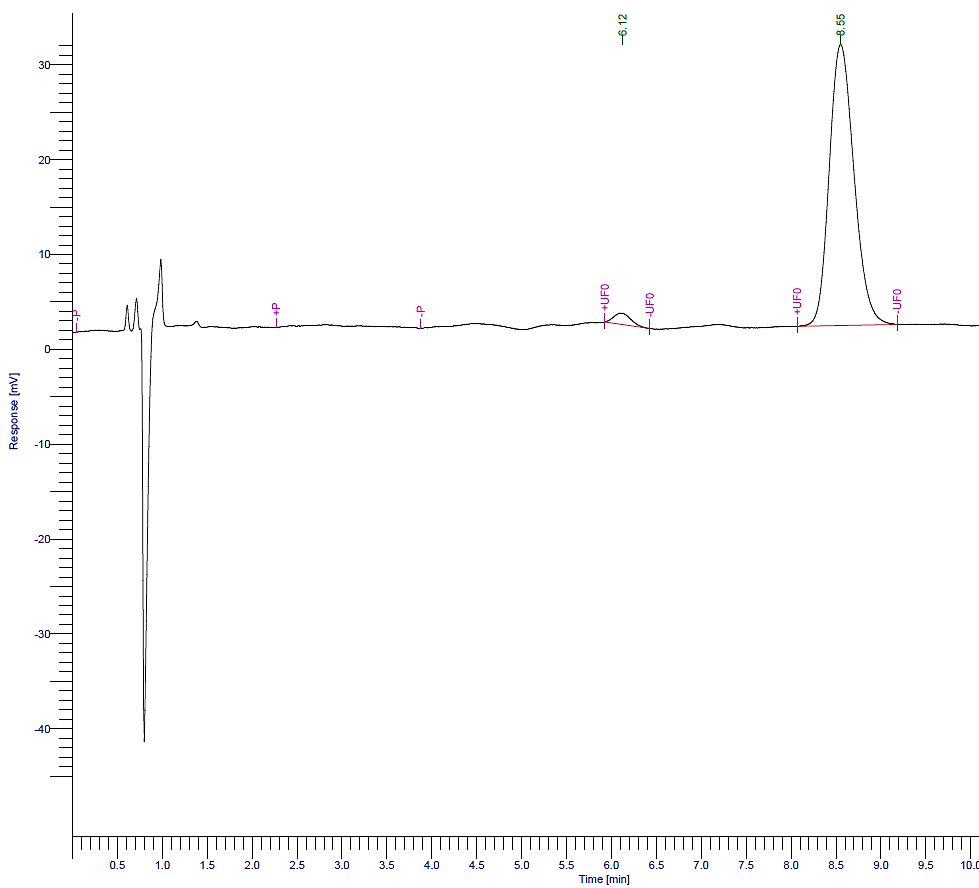


**Characterisation Figure C40.** RP-HPLC trace for UICP11 showing a main peak at retention time 8.55 min.


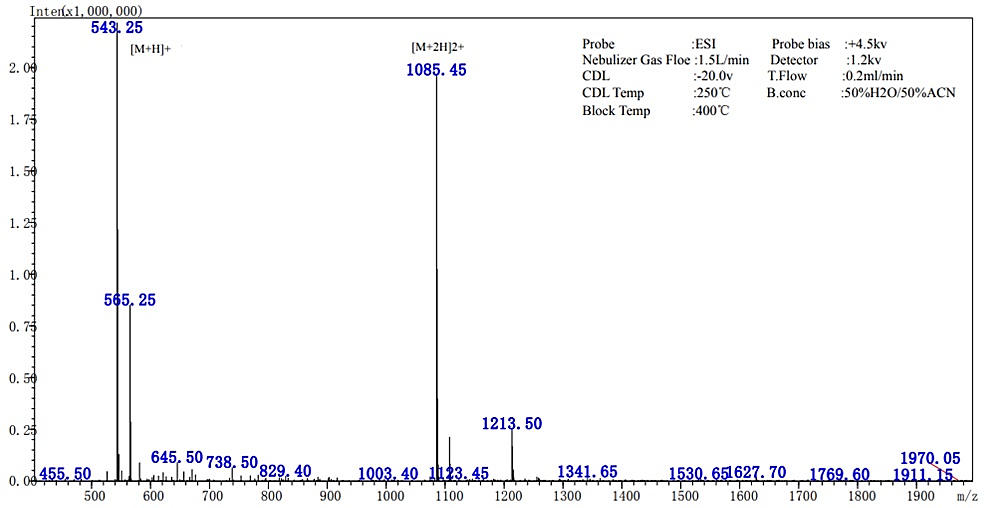


**Characterisation Figure C41.** ESI-MS spectrum for UICP11 showing a main peak of m/z 543.25 [M+H]^+^.

**1672 cm^-1^ C=O stretching**

**1672 cm^-1^ C=O stretching**

**1554 cm^-1^**

**C-N stretching & N-H bending**

**2889 cm^-1^ C-H aliphatic stretching**

**2980 cm^-1^ C-H aromatic stretching**

**3028-3600 cm^-1^ O-H & N-H stretching**

**1251 cm^-1^**

**C-N stretching & N-H bending**

**Characterisation Figure C42.** ATR-FTIR spectrum for UICP11 showing the characteristic functional group peaks.


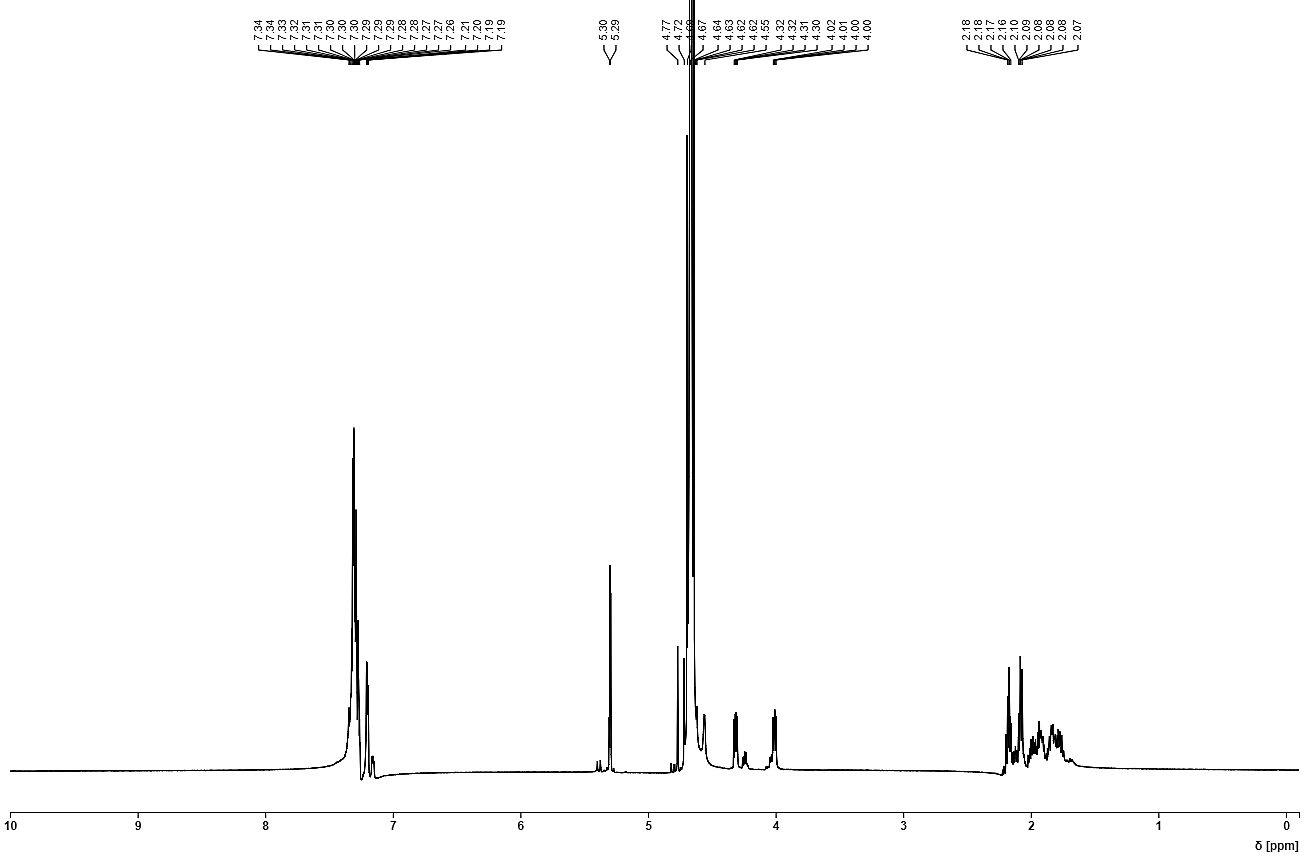


**Characterisation Figure C43.** ^1^H NMR spectrum for UICP11 in D_2_O showing the different proton peaks.


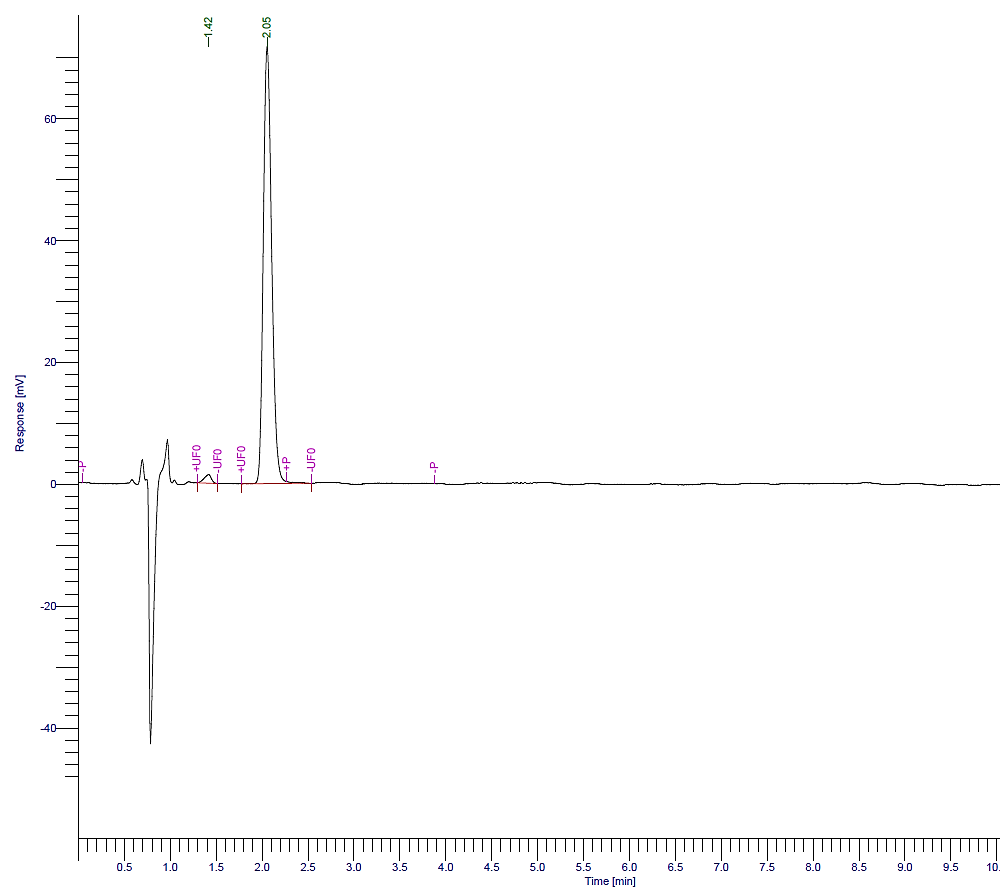


**Characterisation Figure C44.** RP-HPLC trace for UICP12 showing a main peak at retention time 2.05 min.


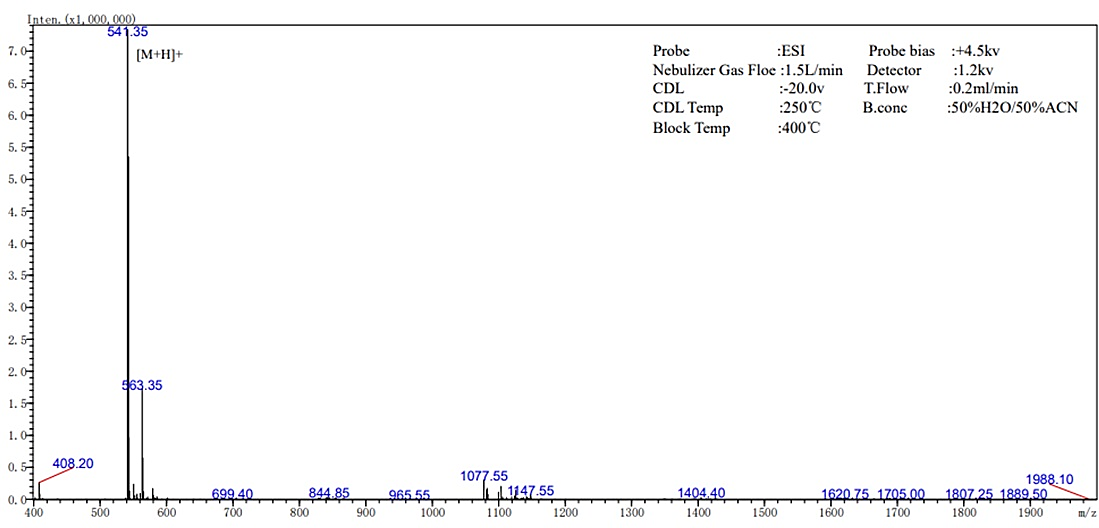


**Characterisation Figure C45.** ESI-MS spectrum for UICP12 showing a main peak of m/z 541.35 [M+H]^+^.

**1646 cm^-1^**

**C=O stretching**

**2873 cm^-1^ C-H aliphatic stretching**

**3346 cm^-1^ O-H stretching**

**3250 cm^-1^ N-H stretching**

**1520 & 1539 cm^-1^**

**CN stretching & NH bending**

**Characterisation Figure C46.** ATR-FTIR spectrum for UICP12 showing the characteristic functional group peaks.


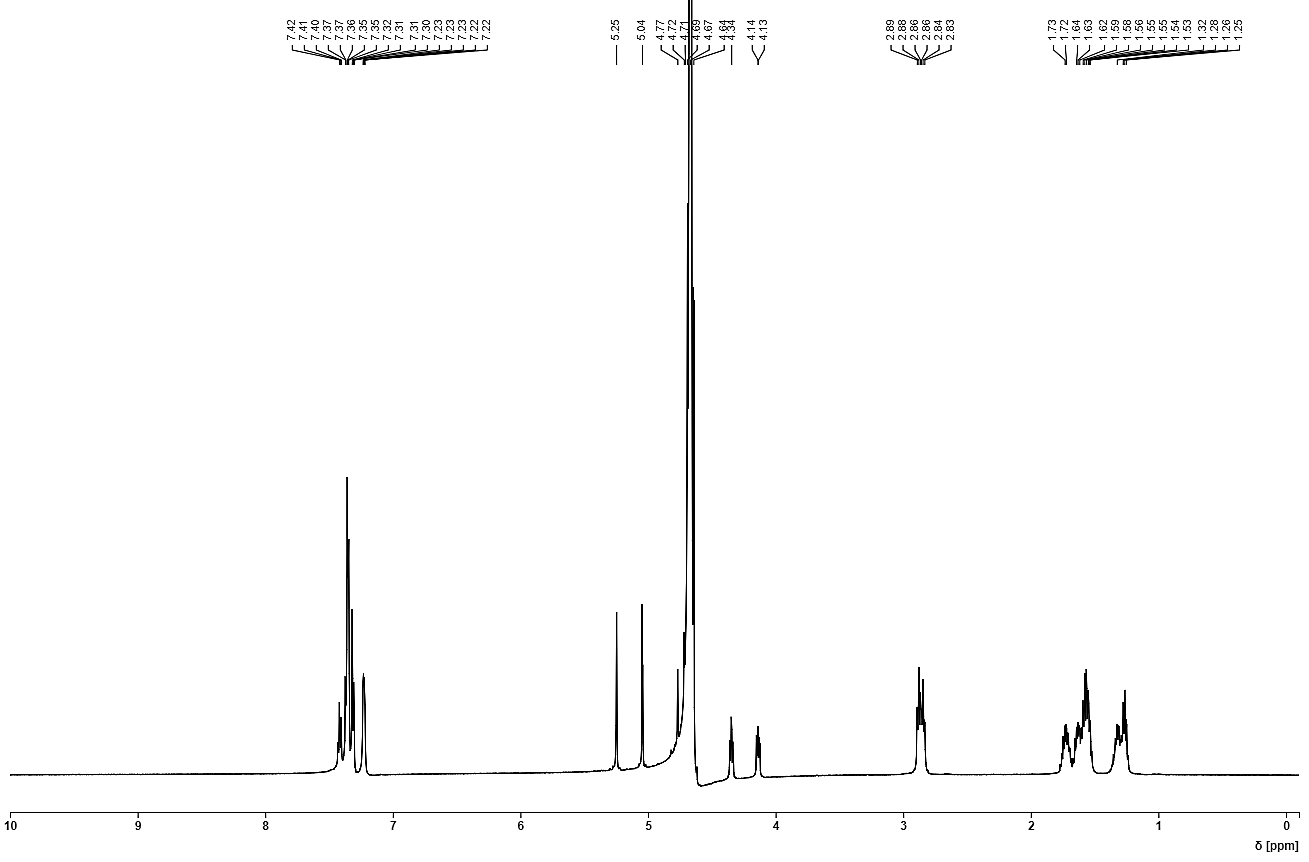


**Characterisation Figure C47.** ^1^H NMR spectrum for UICP12 in D_2_O showing the different proton peaks.


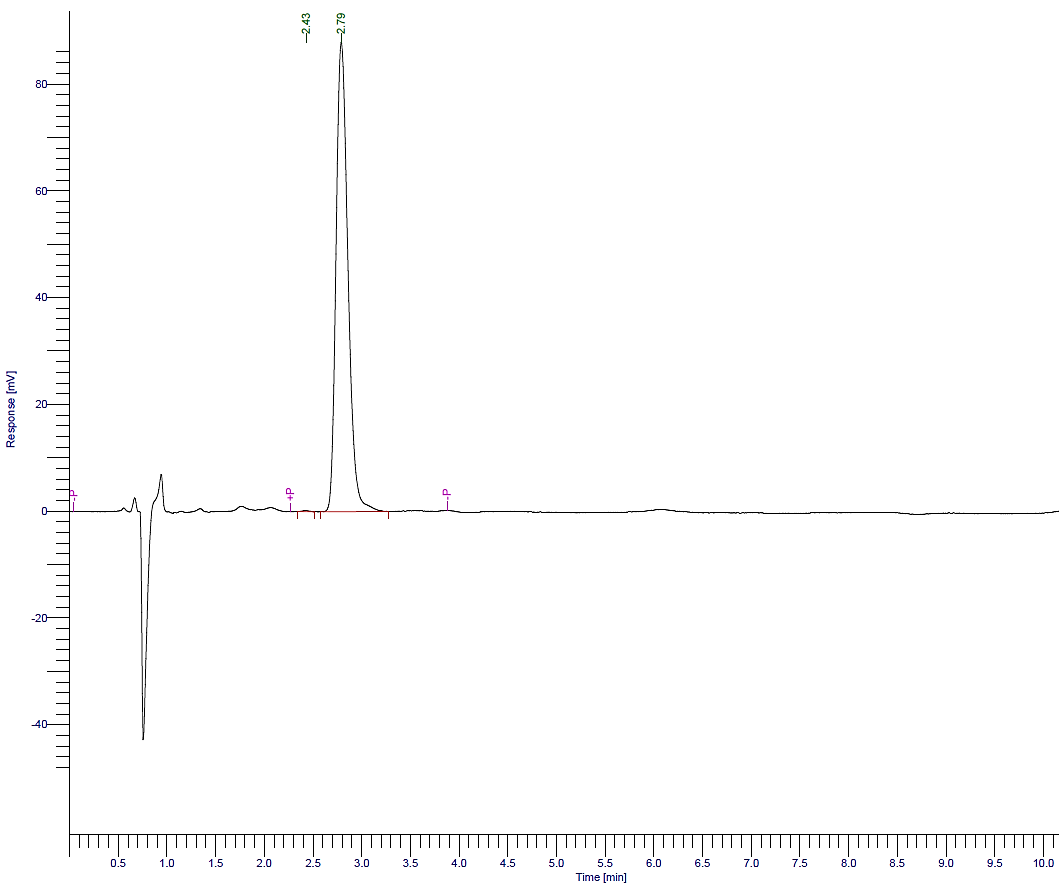


**Characterisation Figure C48.** RP-HPLC trace for UICP13 showing a main peak at retention time 2.79 min.


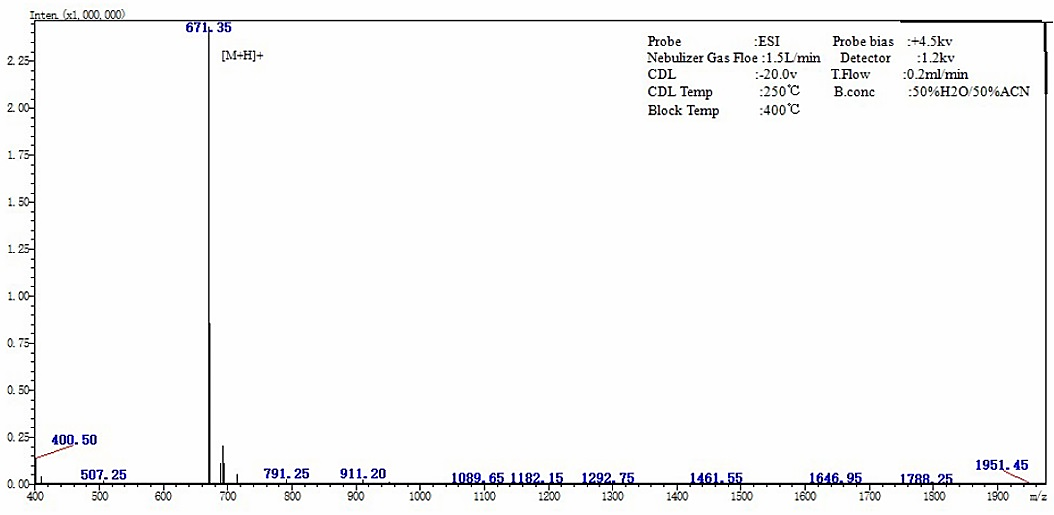


**Characterisation Figure C49.** ESI-MS spectrum for UICP13 showing a main peak of m/z 671.35 [M+H]^+^.

**1251 cm^-1^**

**C-N stretching & NH bending**

**1538 cm^-1^**

**C-N stretching & NH bending**

**1634 cm^-1^ C=O stretching**

**1690 cm^-1^ C=O stretching**

**2976 cm^-1^ C-H aliphatic stretching**

**3050 cm^-1^ C-H aromatic stretching**

**3275 cm^-1^ N-H stretching**

**3307 cm^-1^ O-H stretching**

**Characterisation Figure C50.** ATR-FTIR spectrum for UICP13 showing the characteristic functional group peaks.


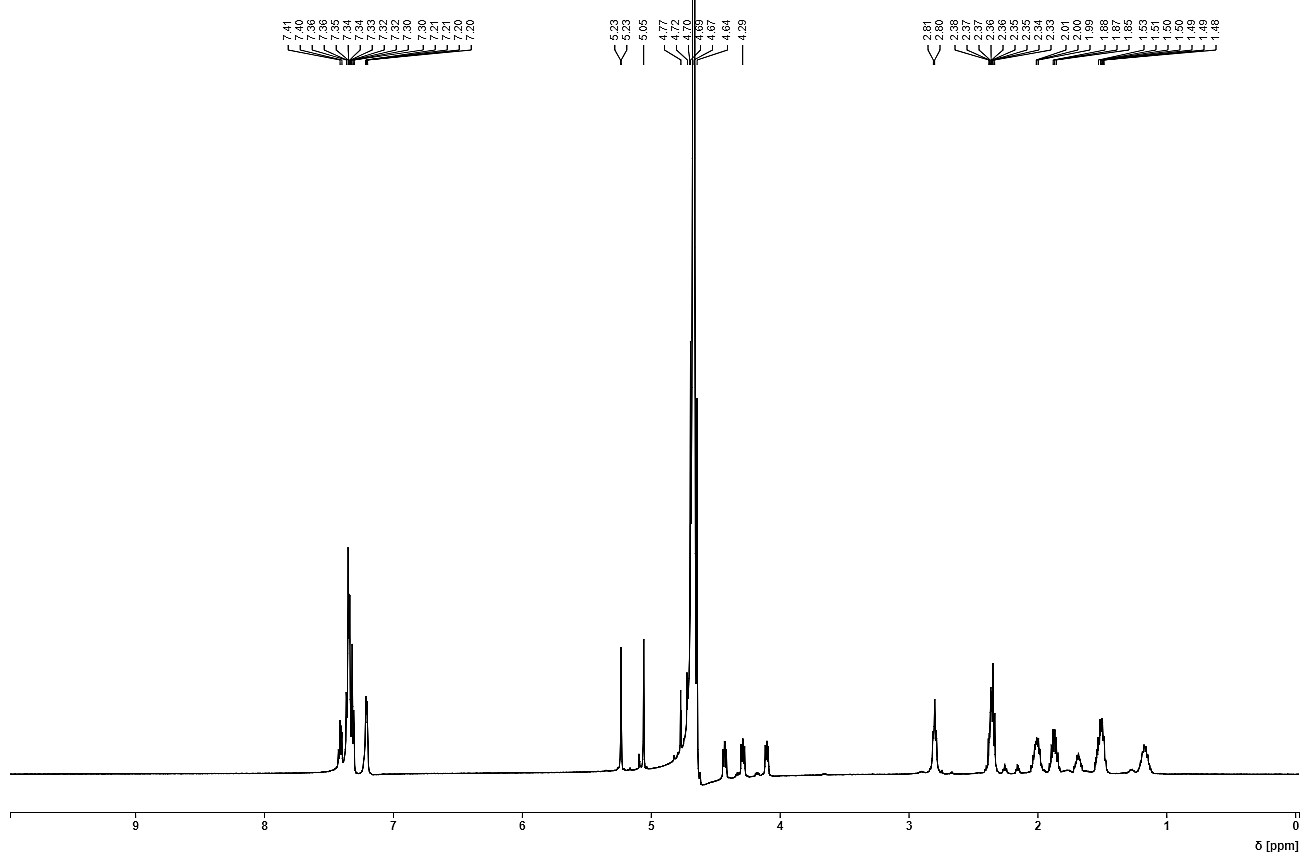


**Characterisation Figure C51.** ^1^H NMR spectrum for UICP13 in D_2_O showing the different proton peaks.


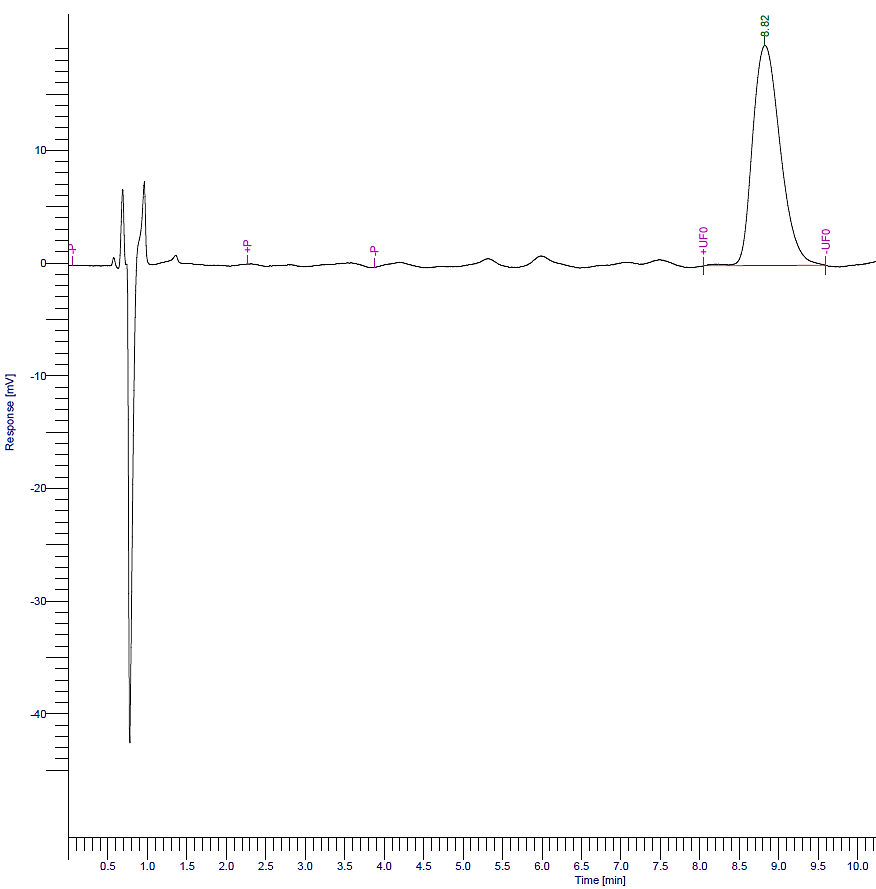


**Characterisation Figure C52.** RP-HPLC trace for UICP14 showing a main peak at retention time 8.82 min.


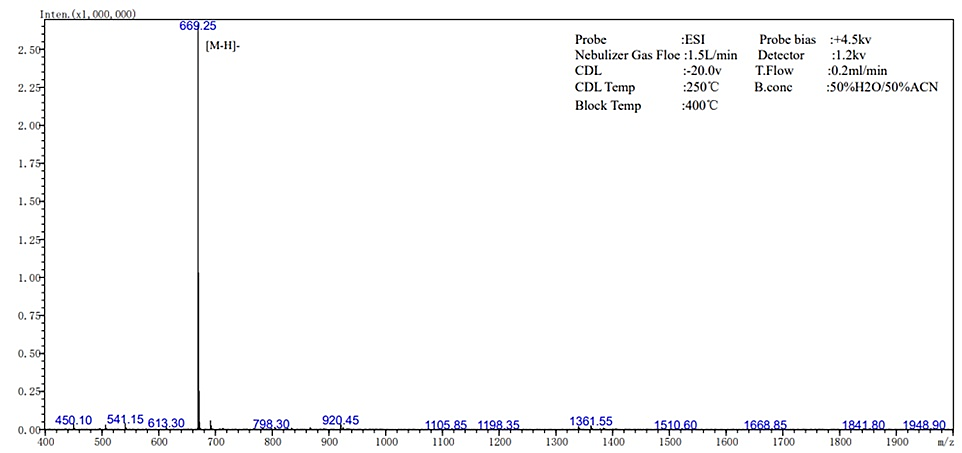


**Characterisation Figure C53.** ESI-MS spectrum for UICP14 showing a main peak of m/z 669.25 [M-H]^-^.

**1250 cm^-1^**

**C-N stretching & N-H bending**

**1530 cm^-1^**

**C-N stretching & N-H bending**

**1624 cm^-1^ C=O stretching**

**1695 cm^-1^ C=O stretching**

**2937 cm^-1^ C-H aliphatic stretching**

**3260 cm^-1^ N-H stretching**

**3055 cm^-1^ C-H aromatic stretching**

**3350-3650 cm^-1^**

**O-H stretching**

**Characterisation Figure C54.** ATR-FTIR spectrum for UICP14 showing the characteristic functional group peaks.


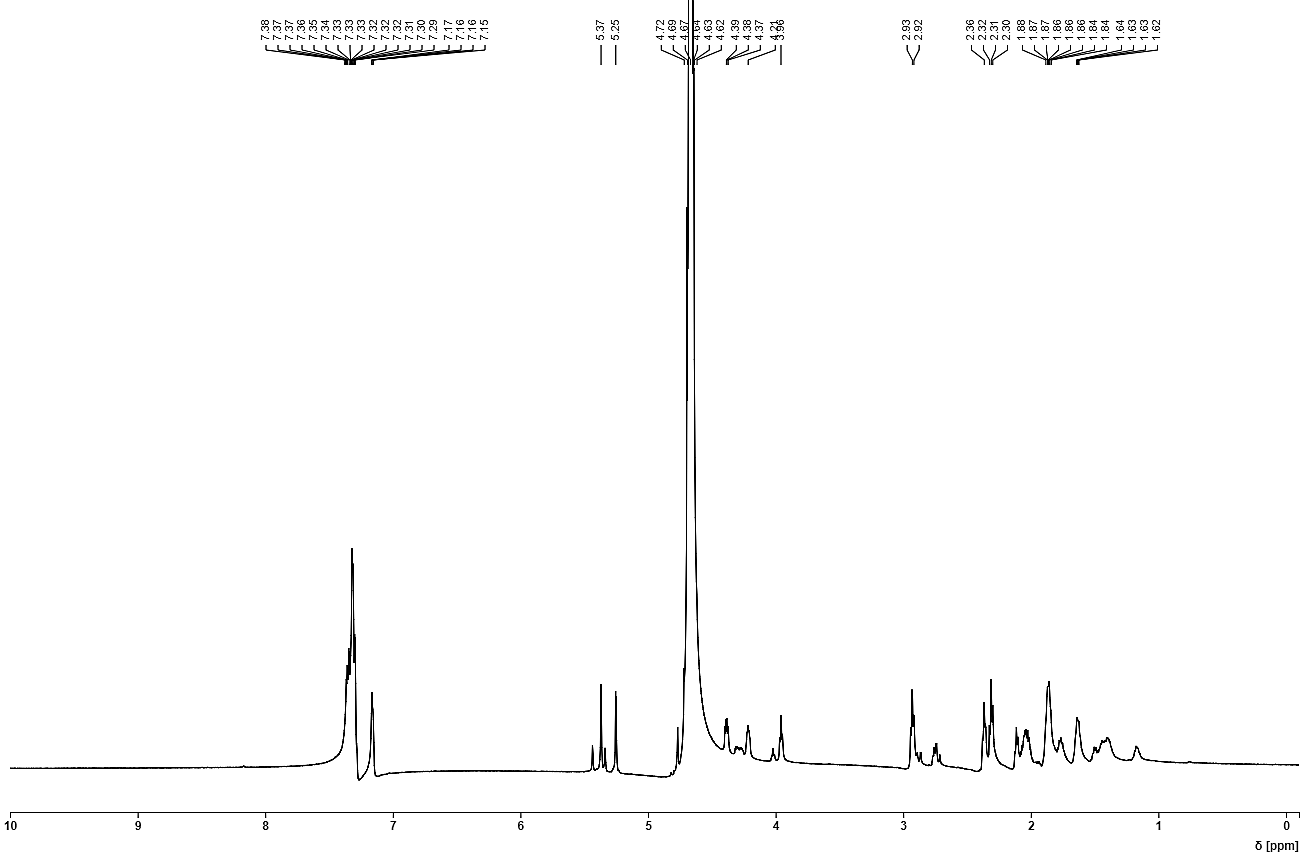


**Characterisation Figure C55.** ^1^H NMR spectrum for UICP14 in D_2_O showing the different proton peaks.


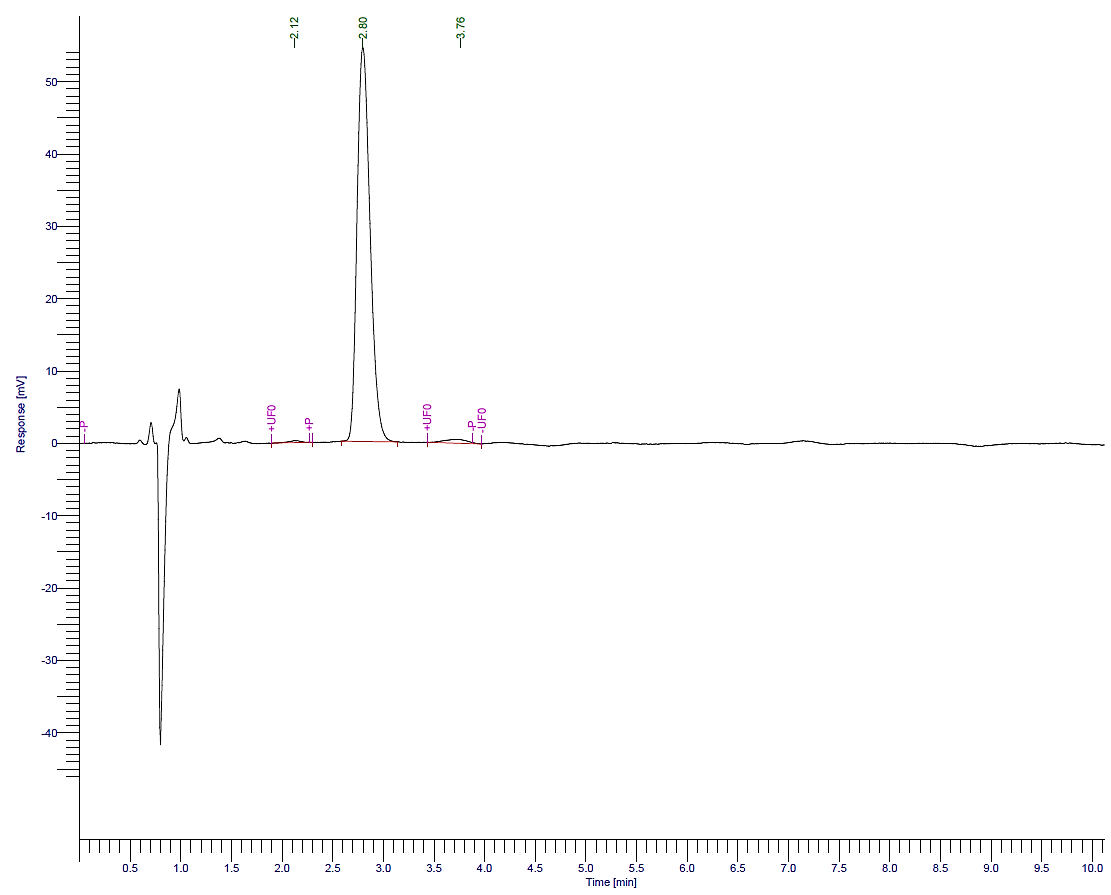


**Characterisation Figure C56.** RP-HPLC trace for UICP15 showing a main peak at retention time 2.80 min.


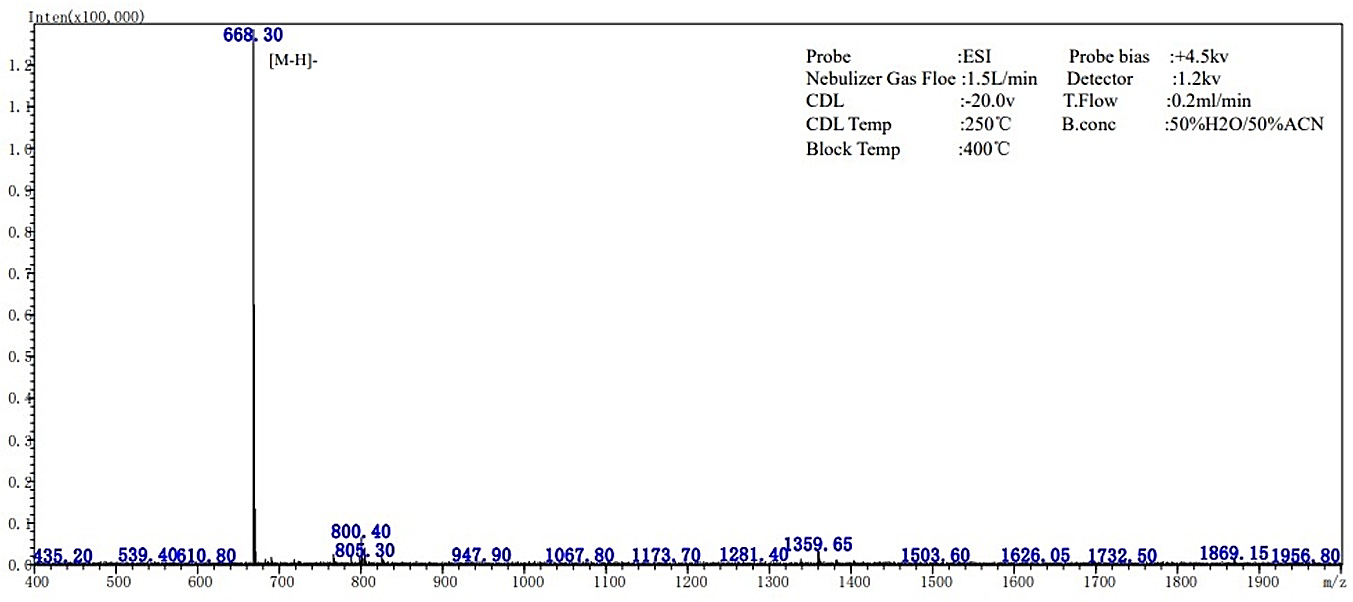


**Characterisation Figure C57.** ESI-MS spectrum for UICP15 showing a main peak of m/z 668.30 [M-H]^-^.

**1251 cm^-1^**

**C-N stretching & N-H bending**

**1541 cm^-1^**

**C-N stretching & N-H bending**

**1678 cm^-1^ C=O stretching**

**2980 cm^-1^ C-H aliphatic stretching**

**3080 cm^-1^ C-H aromatic stretching**

**3660 cm^-1^ O-H & N-H stretching**

**Characterisation Figure C58.** ATR-FTIR spectrum for UICP15 showing the characteristic functional group peaks.


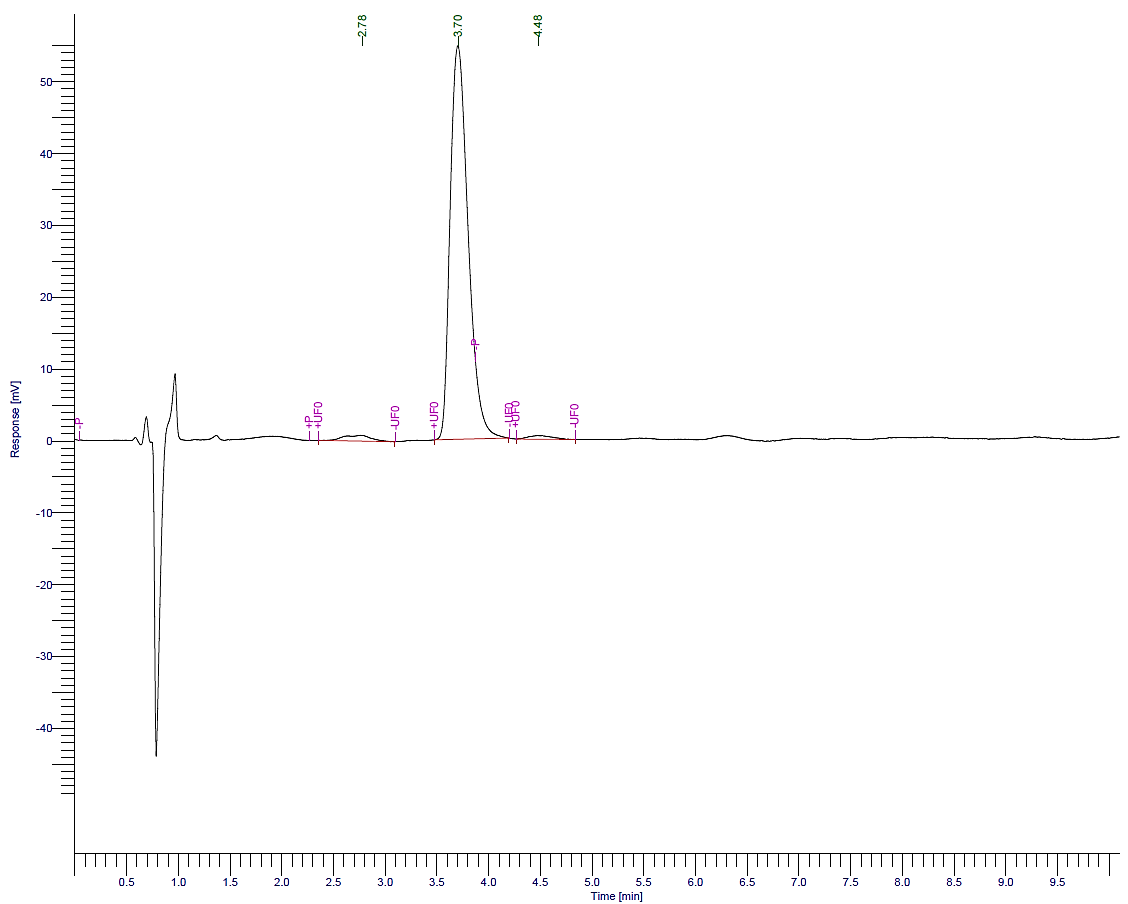


**Characterisation Figure C59.** RP-HPLC trace for UICP16 showing a main peak at retention time 3.70 min.


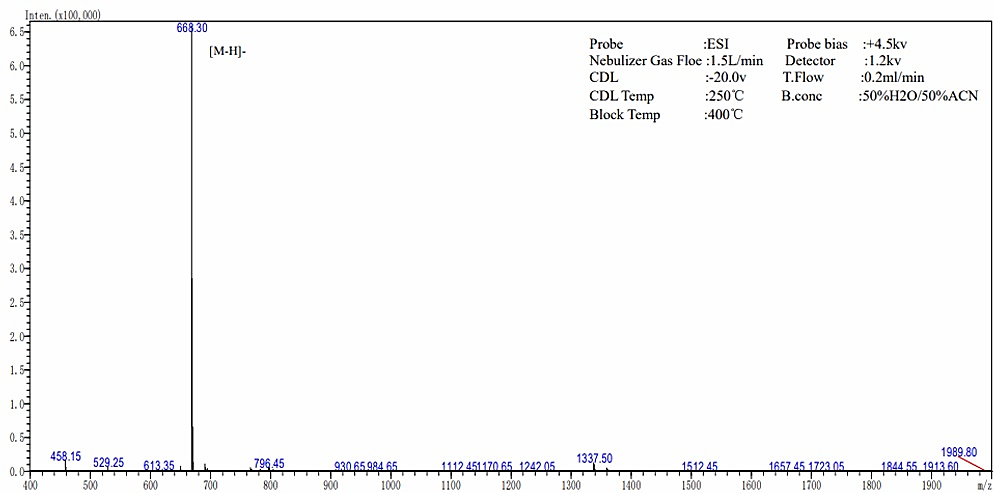


**Characterisation Figure C60.** ESI-MS spectrum for UICP16 showing a main peak of m/z 668.30 [M-H]^-^.

**1248 cm^-1^**

**C-N stretching & N-H bending**

**1559 cm^-1^**

**C-N stretching & N-H bending**

**1678 cm^-1^ C=O stretching**

**2943 cm^-1^ C-H aliphatic stretching**

**3274 cm^-1^ N-H stretching**

**3060 cm^-1^ C-H aromatic stretching**

**3322-4000 cm^-1^ O-H stretching**

**1624 cm^-1^ C=O stretching**

**Characterisation Figure C61.** ATR-FTIR spectrum for UICP16 showing the characteristic functional group peaks.


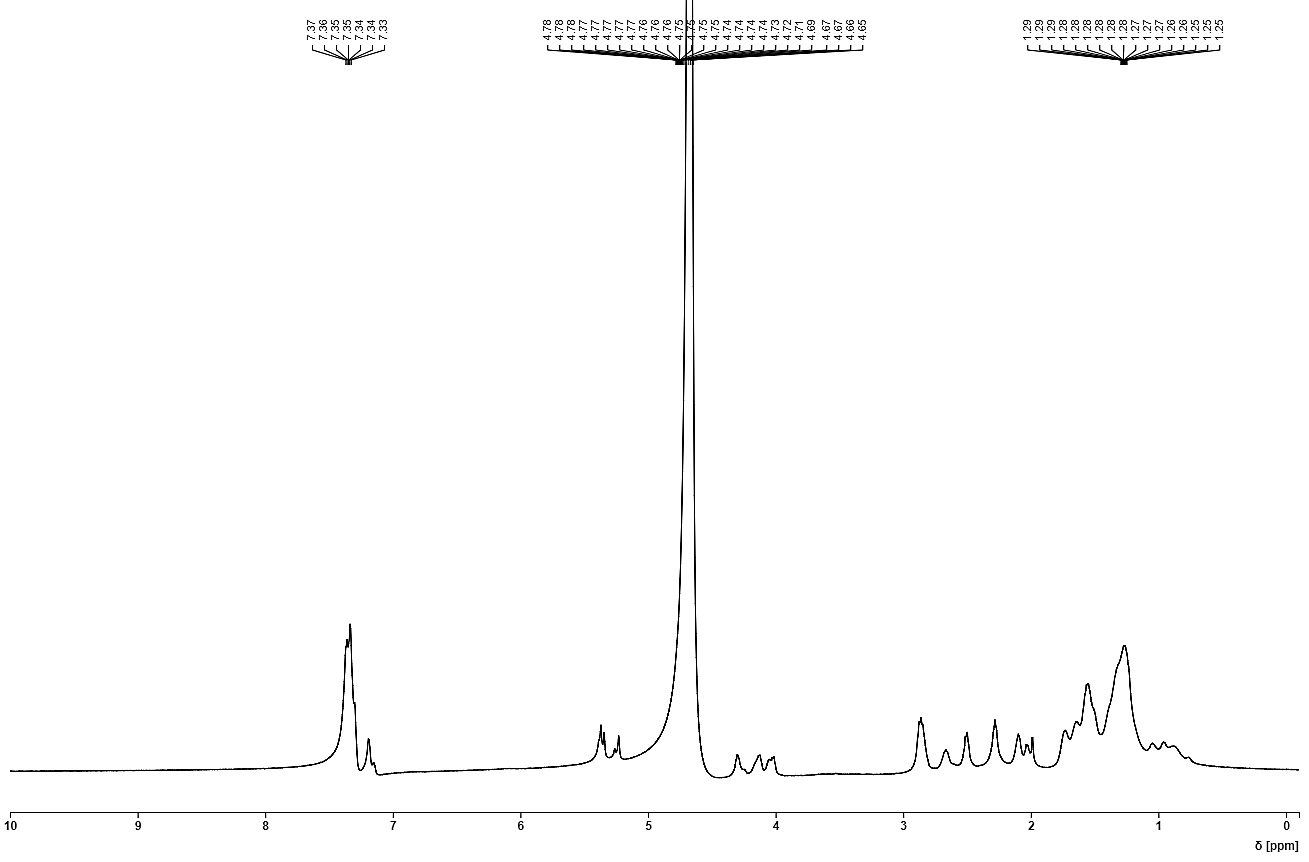


**Characterisation Figure C62.** ^1^H NMR spectrum for UICP16 in D_2_O showing the different proton peaks.
